# IL-6 Mediated Transcriptional Programming of Naïve CD4+ T Cells in Early Rheumatoid Arthritis Drives Dysregulated Effector Function

**DOI:** 10.3389/fimmu.2019.01535

**Published:** 2019-07-03

**Authors:** Laura A. Ridgley, Amy E. Anderson, Nicola J. Maney, Najib Naamane, Andrew J. Skelton, Catherine A. Lawson, Paul Emery, John D. Isaacs, Ruaidhrí J. Carmody, Arthur G. Pratt

**Affiliations:** ^1^Institute of Cellular Medicine (Musculoskeletal Research Group), Newcastle University, Newcastle upon Tyne, United Kingdom; ^2^Leeds Institute of Rheumatic and Musculoskeletal Medicine, Chapel Allerton Hospital, University of Leeds, Leeds, United Kingdom; ^3^Leeds NIHR Biomedical Research Centre, The Leeds Teaching Hospitals Trust, Leeds, United Kingdom; ^4^Directorate of Musculoskeletal Services, Newcastle upon Tyne Hospitals NHS Foundation Trust, Newcastle upon Tyne, United Kingdom; ^5^Institute of Infection, Immunity and Inflammation, University of Glasgow, Glasgow, United Kingdom

**Keywords:** interleukin-6, CD4+ T cell, early rheumatoid arthritis, pathogenesis, transcriptional programming

## Abstract

**Objective:** We have previously shown that increased circulating interleukin-6 (IL-6) results in enhanced CD4+ T cell signaling via signal transduction and activator of transcription-3 (STAT3) in early rheumatoid arthritis (RA). We tested the hypothesis that transcriptional “imprinting” of T-cells by this mechanism skews downstream effector responses, reinforcing immune dysregulation at a critical, but targetable, disease phase.

**Methods:** We modeled naïve CD4+ T cell exposure to pathophysiological concentrations of IL-6 *in vitro*, assessing the dynamic transcriptional and functional consequences for downstream effector cells utilizing microarray and flow cytometry. Fresh blood from treatment-naïve early arthritis patients was phenotyped in parallel for comparison.

**Results:** T cell sensitivity to IL-6 was most marked in the naïve subset, and related to gp130 rather than IL-6R expression. Exposure of healthy naïve CD4+ T cells to IL-6 induced the same STAT3 target genes as previously seen to discriminate RA patients from disease controls. After TCR stimulation IL-6 pre-exposed cells exhibited enhanced proliferative capacity, activation, and a propensity toward Th1 differentiation, compared to non-exposed cells. An entirely analogous phenotype was observed in early RA compared to control CD4+ T cells.

**Conclusions:** Sustained IL-6 exposure at a critical point in the natural history of RA “primes” the adaptive immune system to respond aberrantly to TCR stimulation, potentiating disease induction with implications for the optimal timing of targeted therapy.

## Introduction

Rheumatoid arthritis (RA) is an inflammatory arthropathy characterized by dysregulated adaptive immune responses, in which cytokine networks play an increasingly appreciated orchestrating role during disease initiation (1). We have reported and extensively validated a gene expression signature in circulating CD4+ T cells that discriminates early RA patients from disease controls, and is enriched for signal transduction and activator of transcription-3 (STAT3) regulated genes (2–4). These data, since independently corroborated by others (5, 6), highlight a transcriptional programme of enhanced IL-6 signaling during the earliest clinical stage of RA. Of interest, this pathway's activation is observed most prominently amongst anti-citrullinated peptide autoantibody (ACPA) negative patients, amongst whom evidence for classical autoimmunity as a basis for disease induction has remained elusive. Determining whether our findings reflect an antigen-independent mechanism of disease potentiation—for example through cytokine “priming” of CD4+ T cells—rather than a mere consequence of systemic inflammation, could yield critical insights into pathogenesis and novel strategies for treatment and prevention.

IL-6 signals via gp130, a ubiquitously expressed membrane bound β receptor subunit shared by other cytokines of the IL-6 family, coupled with an α receptor subunit IL-6R (CD126). Surface expression of IL-6R is restricted to certain leukocyte subsets and hepatocytes where it facilitates classical signaling, with other cell types dependent upon a soluble form (sIL-6R; trans-signaling) (7). Biologically divergent consequences of classical and *trans* signaling are now being dissected, along with their respective dominance in driving regulatory vs. inflammatory responses (8). IL-6 has long been known to be important in the induction of experimental models of disease, including collagen-induced arthritis (CIA) and autoimmune inflammatory arthritis (AIA), with mutations in gp130 also resulting in autoimmune arthritis (9–12). Indeed, the efficacy of therapeutically targeting IL-6 signaling in RA emphasizes the relevance of this cytokine to human disease progression (12, 13). However, IL-6 drives aberrant T cell effector function by complex and varied means and those pertinent to the pathogenesis of early RA have yet to be characterized (14, 15).

Here, we sought a deeper understanding of the consequences of IL-6 mediated STAT3 signaling in CD4+ T cells of early RA patients. In particular, we hypothesized that non-specific, chronic exposure of these cells to the cytokine might, through transcriptional imprinting, programme aberrant effector responses following T cell receptor (TCR) activation, and so contribute to tolerance loss and disease progression.

## Materials and Methods

### Subjects

Peripheral blood was obtained from patients recruited from the Northeast Early Arthritis Cohort (NEAC) (3) prior to commencement of immunomodulatory treatment. Clinical characteristics of patients recruited in addition to those previously described from these cohorts are provided in Table 1. Healthy donor blood for *in vitro* culture and dynamic transcriptional profiling was drawn from volunteers into citrate vacuette tubes (Greiner Bio-One, Kremsmünster, Austria). For functional experiments leukocyte reduction system (LRS) cones from platelet donations were used. In relation to *ex vivo* peripheral blood CD4+ T cell Ki67 measurements only, data were similarly available from 25 drug-naïve RA patients recruited from the Leeds Early Arthritis Clinic (LEAC), together with 48 age- and sex-matched healthy donors enrolled as previously outlined (16). Diagnoses of RA were assigned according to contemporaneous classification criteria (17, 18). All donors gave written informed consent for inclusion in the study and ethical approval was obtained from local ethics committees.

**Table 1:** Clinical characteristics of early arthritis patients.

	**Early arthritis**	**Early RA**	
	**“Cohort A”**	**“Cohort B”**	**“Cohort C”**
Number of patients	26	20	14
Age; years^a^	51 (20–82)	66.5 (33–87)	65 (21–89)
Female, *n* (%)	15 (58)	7 (70%)	9 (64)
ESR, mm/second^a^	9 (2–84)	26 (9–122)	21 (9–122)
CRP, g/L^a^	5 (<5–65)	26.5 (4–278)	8.5 (4–150)
ACPA+, *n* (%)	8 (31)	55	57
RF+, *n* (%)	10 (39)	55	57
DAS28 (RA only)^1^	4.8 (1.33–8.46)	5.12 (2.49–7.83)	3.68 (2.12–6.29)
Diagnosis, *n* (%):			
RA:	10 (38%)	20 (all)	14 (all)
Inflammatory, non-RA:	6 (24%)	–	–
OA/non-inflammatory:	10 (38%)	–	–

*Distinct cohorts were recruited for measuring circulating CD4+ T-cell parameters in distinct experiments as indicated in the text. Surface IL-6R and gp130 expression was measured in Cohort A, CD25 expression in Cohort B and intracellular interferon-γ and IL-17 expression following in vitro stimulation in Cohort C*.

a *Median and range are presented; ESR, erythrocyte sedimentation rate; CRP, C-reactive protein; ACPA, anti-citrullinated peptide autoantibody; RF, rheumatoid factor; DAS28, disease activity score, 28 joints; RA, rheumatoid arthritis; OA, osteoarthritis*.

### Serum IL-6 Measurement

IL-6 in patient serum at baseline was measured as previously described (3), using electro-chemiluminescence immunosorbance detection system (Meso Scale Discovery, Gaithersberg, Maryland, USA) according to manufacturer's instructions.

### Cell Subset Isolation

Naïve and memory CD4+ T cells were isolated using a RosetteSep Human CD4+ T cell Enrichment Cocktail (Stemcell Technologies) followed by CD45RO MicroBeads (Miltenyi Biotech), achieving consistent median purities of 82.4 and 78.7%, respectively. For whole CD4+ T cell experiments cells were isolated using monocyte depletion by immuno-rosetting followed by automated magnetic bead-based positive selection (median 99.1% purity; Stemcell Technologies). Peripheral blood mononuclear cells (PBMCs) were isolated by density centrifugation on Lymphoprep (Axis-Shield Diagnostics, Dundee, UK).

### Culture of CD4+ T-Cells

Culture of 1 × 10^6^ cells/ml freshly isolated CD4+ T-cells was in serum-free medium alone (TexMACS, Miltenyi Biotech) or with indicated final concentrations of IL-6 and equimolar sIL-6R (both PeproTech EC) in a 1 ml total volume (24-well plate) or 0.5 ml volume (48-well plate) for 72 h at 37°C with 5% CO_2_. Naïve CD4+ T cells were labeled with 0.5 μM CFSE (eBioscience). Cells were harvested and washed twice, to remove IL-6/sIL-6R, before stimulation for 3–6 days with plate bound anti-CD3 (0.2 or 0.5 μg/ml; eBioscience) and 1 μg/ml soluble anti-CD28 (BioLegend). IL-6 in patient serum at baseline was measured as previously described (3).

### Th1 and Th17 Differentiation

Naïve and memory CD4+ T cells were differentiated toward T-helper 1 (Th1) or T-helper 17 (Th17) cells, respectively, according to a previously developed protocol (19, 20). For Th1 cell differentiation experiments, following incubation with serum-free medium alone vs. in the presence of IL-6/sIL-6R naïve CD4+ T cells were cultured in a total volume of 1 ml in a 24-well plate at 1 × 10^6^ cells/ml in Iscove's modified Dulbecco medium (IMDM) supplemented with 10% FCS. These cells were then cultured with 10 IU/ml IL-2 (Proleukin, Roche, Basel, Switzerland), 1 ng/ml IL-12 (PeproTech), 10 μg/ml anti-IL-4 (BioLegend), and stimulated with anti-CD3/anti-CD28 coated Dynabeads (Invitrogen, Carsbad, California, USA) at 1 bead: 10 cells ratio. For Th17 differentiation, memory CD4+ T cells were cultured in a total volume of 1 ml in a 24 well plate at 1 × 10^6^ cells/ml in IMDM supplemented with 10% serum replacement (Invitrogen). Cells were cultured with 10 ng/ml IL-1β (PeproTech), 10 ng/ml IL-23 (PeproTech), 10 ng/ml TGF-β (PeproTech), and stimulated with anti-CD3/anti-CD28 coated Dynabeads (Invitrogen) at 1 bead: 50 cells ratio. Cells were cultured 37°C with 5% CO_2_ and split or refreshed as required. On day six beads were removed using EasySep magnet and cells were rested in IL-2 (10 IU/ml) for 4 days.

### Multiparameter Flow Cytometry

All antibodies used in this paper were mouse anti-human. Phosflow was performed on unstimulated whole blood as previously described (3), employing the following antibodies: anti-CD3-Pacific Blue (UCHT1), anti-Stat3 (pY705)-Alexa Fluor 647 (4/P-STAT3; all BD Biosciences, Oxford, UK), CD45RA-PerCP-Cy5.5 (HI100; all BD Biosciences, Oxford, UK); CD62L-PECy7 (DREG-56; both BioLegend); anti-CD4-APC-eFluor 780 (SK3; eBioscience, Hatfield, UK); CD62L-PECy7 (DREG-56; BioLegend). BD Phosflow Lyse/Fix and BD Phosflow Perm Buffer III (both BD Biosciences) were used as per the manufacturers' instructions, data collected on a BD FACSCanto II (BD Biosciences) and analyzed using FlowJo (Treestar, Ashland, Oregon, USA) using fluorescence-minus-one (FMO) gating.

To measure constitutive IL-6 receptor expression in CD4+ T-cell subsets, whole blood was stained using CD3-BV510 (UCHT1), CD62L-PECy7 (DREG-56), gp130-PE (2E1B02; all BioLegend), CD4-APCef780 (SK3), IL-6R-PerCPeF710 (47.797.1F2; eBioscience), and CD45RA-FITC (HI100; BD Biosciences) before red blood cell lysis and white blood cell fixation using BD FACS Lysing solution (BD Biosciences), as per manufacturer's instructions. A gating strategy, as depicted in Supplementary Figure 1, was used to determine positive IL-6R or gp130 staining. In brief, debris was excluded by SSC-A vs. SSC-W and lymphocytes were gated based on SSC-A vs. FSC-A. A plot of CD4 vs. either IL-6R or gp130 containing all CD3 positive cells was used to determine IL-6R or gp130 positivity. This positive gate was then copied onto the relevant cell population.

Cultured cells were stained for surface markers in FACS buffer (PBS containing 0.5% BSA, 0.1% sodium azide and 2 mM EDTA) in the presence of 4 μg/ml human IgG for 30 min at 4°C; the following antibodies were used: CD4-APCeF780 (SK3; eBioscience), CD3-BV510 (UCHT1), CD25-PECy7 (BC96), CD40L-PE (24-31; BioLegend). Cells were fixed with 1% formaldehyde prior to acquisition. Prior to intracellular cytokine staining cells were cultured for 1 h in the presence of 10 ng/ml PMA and 1 μg/ml ionomycin, before the addition of 1 mg/ml Brefeldin-A (BFA) (all Sigma Aldrich) for 4 h. BFA-exposed cells cultured in the absence of PMA/ionomycin were stained in parallel and used as negative controls for gating. After staining with Zombie Aqua viability dye (BioLegend), surface antibodies were CD3-PB or CD3-BUV395 (UCHT1) and CD4-PerCP or CD4-BV786 (SK3; BD Biosciences); fixation and permeabilization was with the FoxP3/transcription factor staining buffer (eBioscience) according to manufacturer's instructions, and intracellular staining was with IFN-γ-FITC(4S.B3; eBioscience) and IL-17-APC-Cy7 (BL168; BioLegend).

Constitutive intracellular levels of Ki67 were measured in unstimulated early RA/control PBMC by staining with Zombie Aqua viability dye (BioLegend) and then CD4-PerCP (SK3; BD Biosciences) before fixation/permeabilization and staining with Ki67-PE (B56; BD Biosciences) or isotype control (MOPC-21; BD Biosciences), again using FoxP3/transcription factor staining buffer. For measurement of constitutive surface CD25 expression, staining was with CD25 PECy7 (BC96, BioLegend), CD3-BUV395 (UCHT1, BioLegend), and CD4-FITC (RPA-T4, BioLegend), gated using CD3 positive, CD4 negative cells. Cells stained for intracellular proteins were collected on a FACS Canto II or Fortessa X20 (BD Biosciences) and analyzed using FlowJo (Treestar, Ashland, Oregon, USA), as before. Debris was excluded by SSC-A vs. SSC-W and live cells were gated based on exclusion of the viability dye. CD4+ T cells were gated as CD3+CD4+ and further examined, with further gating against negative control cells. Proliferation was calculated using the proliferation tool on FlowJo and reported as division index, the average number of cell divisions a cell in the original population has undergone.

### Gene Expression of Naïve and Memory CD4+ T Cells

RNA was extracted from naïve and memory CD4+ T-cells at indicated experimental time-points using RNeasy Mini kit (Qiagen), as per manufacturer's instructions. RNA was quantified using a Nanodrop 1,000 UV Spectrophotometer (Thermo Fisher Scientific) prior to generation/hybridization of cRNA to the Illumina Human HT-12 v4.0 microarray (Illumina, San Diego, USA). Raw data processing and further data analysis was performed in R software version 3.3.0, using the Bioconductor packages. Variance-stabilizing transformation (VST) and Robust Spline Normalization (RSN) were carried out in the lumi package and differentially expressed genes were identified using the moderated paired *t*-test implemented in the limma package, with a fold-change of 1.5 and *p* < 0.05 after correction for multiple testing using the Benjamini-Hochberg method. Pathway analysis of differentially expressed gene lists was carried out using Ingenuity Pathway Analysis (IPA; Qiagen). Hypergeometric testing was carried out in order to determine the probability of genes differentially expressed between early RA and disease controls occurring in IL-6 exposed naïve CD4+ T cells. Expression data used in this study are available in the Gene Expression Omnibus database (accession number GSE131866; https://www.ncbi.nlm.nih.gov/geo).

### Statistics

Additional statistical analysis was performed using GraphPad prism 5.03 (GraphPad Software Inc). Non-parametric analysis of variance (Friedman's test; Dunn's *post hoc* pairwise analyses), Mann-Whitney *U*-tests and Wilcoxon matched pairs tests were used for comparisons of multiple paired groups, two unpaired groups or two paired groups, respectively. Spearman's rank was used for correlation analyses. *P*-values of < 0.05 were considered significant.

## Results

### Amongst Circulating CD4+ T Cells of Early Arthritis Patients, True Naïve Cells Are Maximally Sensitive to IL-6, Reflecting Their Increased Membrane Gp130 Expression

**Figure 1:**
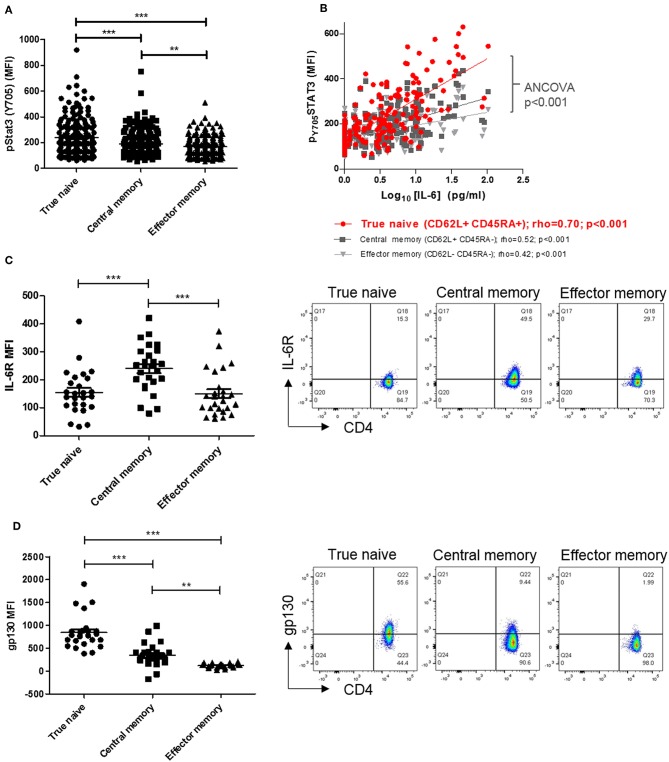
**(A)** pSTAT-3 expression was determined in true naïve (TN; CD62L+ CD45RA+), central memory (CM; CD62L+ CD45RA-), and effector memory (EM; CD62L- CD45RA-) CD4+ T-cells in peripheral blood of a previously described early arthritis patient cohort (3) using flow cytometry. **(B)** In the same cohort, the relationship between pSTAT-3 and paired circulating interleukin (IL)-6, measured by MSD immunoassay, was assessed by Spearman correlation coefficients (rho); gradients of best-fit lines differ significantly (analyses of covariance *p* < 0.001). Amongst newly-enrolled early arthritis patients (cohort A; see Table 1), surface IL-6R expression **(C)** and surface gp130 expression **(D)** was determined in TN, CM and EM CD4+ T-cells in the peripheral blood using flow cytometry [left panels; *n* = 26; ^**^p < 0.001, ^***^p < 0.0001, non-parametric analysis of variance (Friedman's) with Dunn's *post hoc* pairwise analyses; associated *p*-value are depicted]. Exemplar FACS plots are also shown (right panels).

Previous investigation of circulating lymphocytes from early arthritis patients revealed constitutive pSTAT3 levels to be higher, and correlate with paired serum IL-6 concentration more strongly, in CD4+ T cells than in CD8+ T-cells or B-cells (3). To extend these findings and identify a CD4+ T cell subset most “sensitive” to circulating cytokine, we first compared constitutive pSTAT3 levels in true naïve (TN), central memory (CM), and effector memory (EM) CD4+ T cells [denoted CD45RA+ CD62L+, CD45RA- CD62L+ and CD45RA- CD62L-, respectively (21)] in the same cohort of early arthritis patients (3). We observed constitutive pSTAT3 levels to be highest, and to correlate most strikingly with paired serum IL-6, in the TN CD4+ T cell sub-population (Figures 1A,B). Cellular sensitivity to IL-6 could be governed by the availability of membrane-expressed α and/or β receptors, which may in turn reflect cellular development (22, 23). Therefore, in fresh blood from a newly recruited cohort of early arthritis patients (described in Table 1, Cohort A) we compared receptor expression amongst TN, CM, and EM CD4+ T cells. Maximal IL-6R expression was observed on central memory cells, whereas expression of gp130 was highest on the true naïve population (median fluorescence intensity, MFI; Figures 1C,D; analogous % positive data and gating strategy presented in Supplementary Figures 1, 2). These results suggest cell surface availability of gp130, rather than IL-6R, restricts CD4+ T cell sensitivity to circulating IL-6 in untreated early arthritis.

### *In vitro* Exposure of Naïve CD4+ T Cells to IL-6 Prior to TCR Stimulation Establishes a Distinctive Dynamic Transcriptional Programme

**Figure 2:**
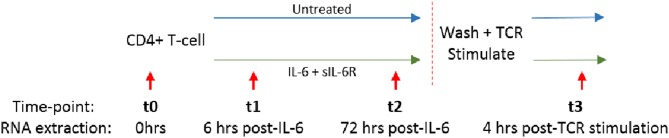
Naïve or memory CD4+ T cells were isolated from 3 healthy donors and cultured with 0.5 ng/ml IL-6 and equimolar sIL-6R for 72 h prior to washing and stimulation with 0.2 μg/ml anti-CD3 and 1 μg/ml anti-CD28 for 4 h. RNA was extracted at baseline (t0), after 6 or 72 h exposure to IL-6 (t1, t2) and 4 h post-TCR stimulation (t3) as depicted by the red arrows.

We and others have shown that increased circulating IL-6 levels are present in the serum of early RA patients compared to disease controls, and are even detectable in sera from ACPA positive individuals yet to develop the disease (2, 3, 24, 25). We hypothesized that sustained exposure of naïve CD4+ T cells to IL-6 might imprint them with a distinct transcriptional programme, whose molecular and functional consequences following subsequent TCR ligation was relevant to disease development. Naïve or memory CD4+ T cells freshly isolated from healthy donors (*n* = 3) were cultured in serum-free medium for 72 h with or without a pathophysiologically relevant (0.5 ng/ml), (2, 3), final concentration of IL6 and equimolar sIL-6R (engaging both classic and *trans*-signaling pathways), before cytokine removal by washing, and then stimulation with anti-CD3/anti-CD28 antibodies. RNA was isolated for global gene expression analysis after 6 and 72 h of culture (time-points *t1* and *t2*, respectively), and again 4 h after subsequent TCR stimulation (*t3*); this experimental set-up is summarized in Figure 2.

**Figure 3:**
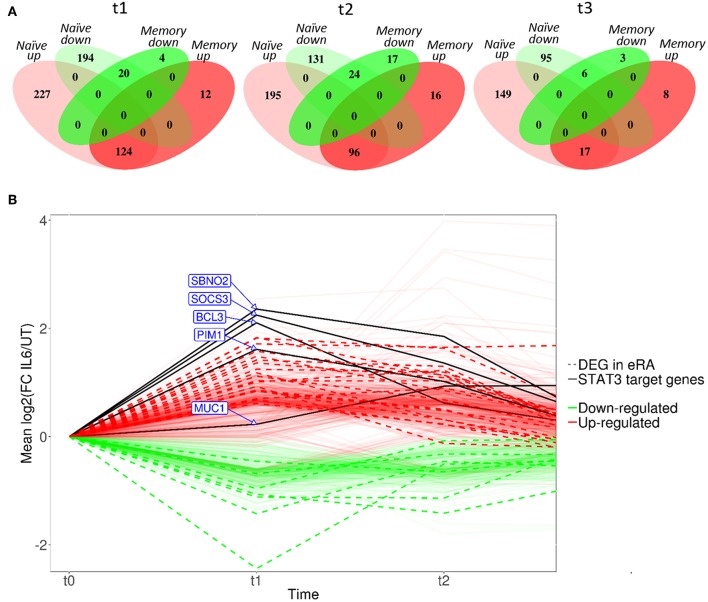
**(A)** Venn diagrams showing the number of differentially expressed genes between untreated and IL-6 pre-exposed naïve and memory CD4+ T cells isolated from 3 healthy donors at t1–t3 using a moderated paired *t*-test with fold change of 1.5 and Benjamini-Hochberg adjusted *p*-value of <0.05. **(B)** Dynamic profiles of genes significantly dysregulated at indicated time-points amongst naïve CD4+ T-cells. Fold- expression in IL-6 exposed cells is depicted relative to that in non-exposed cells at each time-point (FC > 1.5, corrected *p* < 0.05 for inclusion). Genes previously observed to be differentially expressed (DE) in early RA (eRA) are indicated (heavy lines), and specific STAT-3 targets labeled (solid lines), FC, fold-change in expression, see text.

Compared with culture in medium alone, exposure of naïve CD4+ T cells to IL-6/sIL-6R altered the expression of 565 genes by ≥1.5-fold at 6 h (*t1*; adjusted *p* < 0.05), with only 160 genes similarly modulated amongst their memory CD4+ T-cell counterparts (Figure 3A; gene lists provided in Supplementary Tables 1, 2). A similar pattern was seen at 72 h (*t2*; 446 and 153 genes in naïve and memory CD4+ T cells, respectively; Supplementary Tables 3, 4), as well as at 4 h following subsequent removal of IL-6 and TCR stimulation (*t3*; 267 vs. 34 genes; Supplementary Tables 5, 6). As further depicted in Figure 3A, transcripts impacted in memory cells corresponded for the most part to a subset of those impacted in naïve cells. These data confirmed at the transcriptional level that naïve CD4+ T cells are markedly more sensitive to sustained IL-6 exposure, and suggested the persistence of a unique expression programme that was maintained over time, even after removal of the cytokine and subsequent TCR stimulation. Our remaining experiments modeling IL-6 exposure therefore focused on naïve cells alone.

### IL-6 Transcriptional Programme Mirrors Previously Described Early RA CD4+ T Cell Signature and Suggests Dysregulated Proliferative Capacity and Survival Pathways

Scrutiny of the genes observed to be up-regulated in naïve CD4+ T cells after 6 h of IL-6 exposure (*t1*) compared with untreated cells confirmed the presence of a substantial component of our previously described 12-gene signature that discriminates early RA patients from disease controls, including genes known to be regulated by STAT-3 (2, 4) (Figure 3B). Induction of these STAT-3-regulated genes, including BCL3, PIM1, SOCS3, SBNO2, and MUC1, was achieved or maintained after 72 h of IL-6 exposure (*t2*; Figure 3B). Indeed, following this sustained *in vitro* IL-6 exposure, dysregulated genes overlapped more than could be expected by chance with an extended list of 96 genes differentially expressed between unstimulated cells of early RA patients and controls [Pratt et al. (2)]. Supplementary Gene List 1; *p* = 3.42 × 10^−14^ at *t2*. These data strongly support the biological relevance of the transcriptional programme imprinted in naïve CD4+ T cells by IL-6 exposure in our *in vitro* model.

We next used pathway analysis to examine the potential molecular consequences of sustained naïve CD4+ T cell IL-6 exposure on effector function following subsequent TCR-stimulation. We focused on the list of 267 genes differentially expressed between untreated and IL-6 pre-exposed cells 4 h after cytokine removal and TCR stimulation, (*t3*; Supplementary Table 5). An over-representation of genes involved in biological functions associated with increased cell growth and proliferation, cell movement, cell development, and cell death and survival was seen. This is consistent with the capacity of IL-6 to imprint a molecular programme amongst naïve CD4+ T cells that mediates hyper-proliferative effector function.

### Hyper-Proliferative, Activated Effector Phenotype Is a Confirmed Consequence of Prior Naïve CD4+ T Cell IL-6 Exposure, Along With Th1 Differentiation Propensity

**Figure 4:**
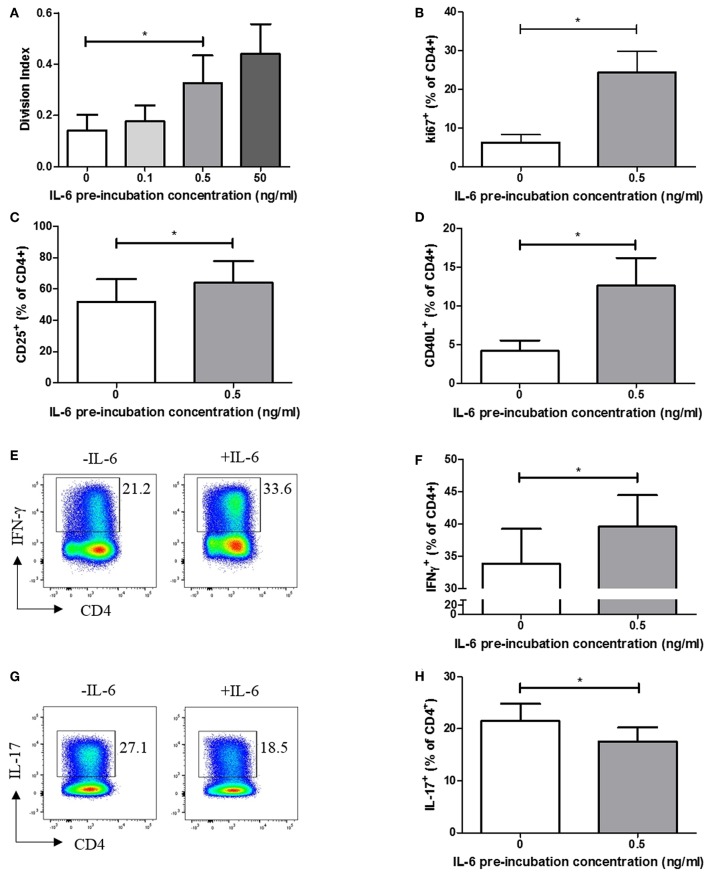
**(A)** Naïve CD4+ T-cells isolated from 6 healthy donors display increased division index after 6 days anti-CD3/CD28 stimulation following prior exposure to increasing concentrations of IL-6. **(B)** CD4+ T cells isolated from healthy donors display increased Ki67 expression after 3 days anti-CD3/CD28 stimulation following prior exposure to 0.5ng/ml IL-6. Expression of CD25 **(C)** and CD40L **(D)** is increased in naïve CD4+ T-cells after 6 days anti-CD3/CD28 stimulation following prior exposure to 0.5 ng/ml compared to naïve CD4+ T-cells with no prior exposure to IL-6. **(E,F)** Increased level of IFN-γ producing cells from naïve CD4+ T cells differentiated toward Th1 following exposure to 0.5 ng/ml IL-6 and equimolar sIL-6R prior to differentiation. **(G,H)** Decreased level of IL-17 producing cells from memory CD4+ T cells differentiated toward Th17 following exposure to 0.5 ng/ml IL-6 and equimolar sIL-6R prior to differentiation. *N* = 6; *p < 0.05, non-parametric analysis of variance (Friedman's) with Dunn's *post hoc* pairwise analyses **(A)** and Wilcoxon matched-pairs signed rank test **(B–D,F,H)**; associated *p*-values are depicted.

We carried out functional studies to investigate whether the effector phenotype of stimulated CD4+ T cells differed following exposure to IL-6, and whether it could be explained by the dynamic transcriptional programme described above. CFSE labeling experiments, in which naïve CD4+ T cells were exposed to a range of IL-6 concentrations in our *in vitro* model, before being washed to remove cytokines and stimulated via the TCR, demonstrated a dose-dependent increase in proliferative capacity at 6 days (Figure 4A). This increase in proliferation is significant at the pathophysiologically relevant 0.5 ng/ml IL-6 pre-exposure concentration used in the expression-profiling experiments, being recapitulated when using intracellular Ki67 as an indicator of the proportion of cycling cells (Figure 4B). Further phenotyping of stimulated cells showed prior exposure to IL-6 and subsequent cytokine removal/TCR stimulation also enhanced expression of the activation markers CD25 and CD40L (Figures 4C,D). In the light of reported roles for IL-6 (14), we anticipated that sustained, prior exposure to it would result in altered T-helper cell differentiation even if the cytokine was absent during TCR stimulation. This was shown to be the case when naive CD4+ T cells were stimulated under Th1-skewing conditions following prior exposure to IL-6 and eqimolar sIL-6R, with increased IFN-γ production seen (Figures 4E,F). In contrast, when IL-6/sIL-6R pre-exposed memory CD4+ T cells were stimulated under Th17-skewing conditions reduced IL-17 production was observed (Figures 4G,H).

These findings suggest that pre-exposure of naïve CD4+ T cells to IL-6 results in an activated, hyper-proliferative effector cell phenotype with a propensity for Th1-skewing.

### Effector Phenotype of IL-6 Primed CD4+ T Cells *in vitro* Is Recapitulated in Early RA

**Figure 5:**
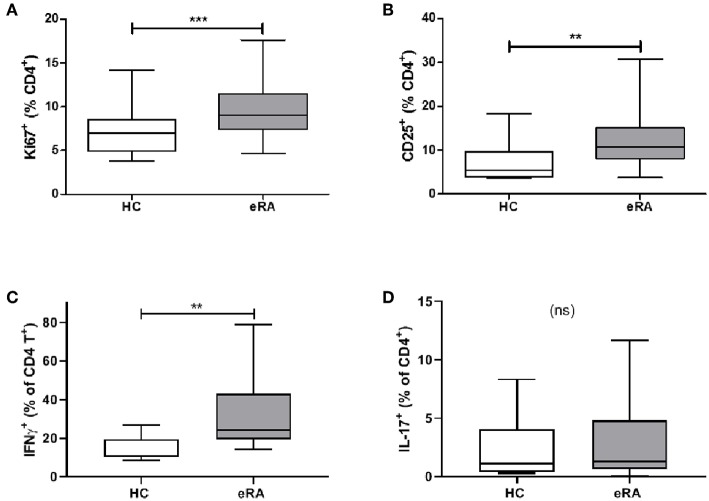
**(A)** PBMC were isolated form peripheral blood of healthy controls (HC) and early drug-naïve RA patients (eRA). Expression of Ki67 in CD4+ T cells was assessed in a previously described cohort (16) by flow cytometry. **(B)** Expression of CD25 in CD4+ T cells from 16 healthy controls and 20 eRA patients in cohort B was assessed by flow cytometry. **(C,D)** CD4+ T cells were isolated from 9 healthy controls and 14 eRA patients in cohort C, and stimulated for 6 days with 0.5 μg/ml anti-CD3 and 1 μg/ml anti-CD28; IFN-γ **(C)** or IL-17 **(D)** were assessed by flow cytometry. **p < 0.001, ***p < 0.0001; Mann Whitney test.

We sought parallels in effector phenotype between “IL-6 primed” effector CD4+ T cells, assessed in our *in vitro* model of prior IL-6 exposure, and *ex vivo* CD4+ T cells from drug-naïve early RA patients. Used as a marker for proliferative capacity, Ki67 was found to be significantly up-regulated in CD4+ T cells of a previously described early RA patient cohort (16) compared with healthy donors (Figure 5A). Moreover, a higher proportion of CD4+ T cells expressed CD25 amongst newly recruited early RA patients (*n* = 20; characteristics in Table 1; Cohort B) compared with 16 healthy donors, Figure 5B. Finally, compared with nine healthy donors a higher proportion of early RA patient CD4+ T cells produced IFN-γ following *ex vivo* TCR stimulation (Figure 5C), with no comparable increase in IL-17 production (Figure 5D; *n* = 14; patient characteristics in Table 1; Cohort C). Clear parallels may therefore be drawn between the functional profile of *ex vivo* CD4+ T cells of early RA patients and those of healthy donors subjected to sustained IL-6 exposure prior to stimulation.

## Discussion

In the current investigation we have extended and built upon our previous findings (2–4) highlighting a potential mechanism via which IL-6 mediated transcriptional imprinting of CD4+ T cells in the earliest stages of RA may programme effector responses of pathophysiological relevance. Several observations of interest arise.

First, we showed that naive CD4+ T cells are more sensitive than memory cells to circulating IL-6 at the level of STAT-3 phosphorylation and its downstream transcriptional consequences. We demonstrated that this was particularly the case for the CD45RA+CD62L+ TN sub-population (18), within which surface expression of the cytokine's β-receptor subunit gp130 (but not it's α-receptor counterpart IL-6R) is also maximal. By contrast, CD45RA-CD62L+ CM CD4+ T cells (whose relative surface-expression of receptor subunits was inverted) displayed diminished sensitivity to circulating IL-6. On one level, these observations indicate that the expression of IL-6 α- and β-receptor subunits is reciprocally linked to the CD45 isoform-defined “antigen experience” of T-cells that express lymph node-homing receptors—adding important nuance to previous descriptions in human populations (22, 26). On another, having previously observed that circulating sIL-6R is always present at molar concentrations well in excess of corresponding IL-6 in early arthritis sera (median 60 ng/ml) (3), we interpret the fact that surface gp130 apparently acts as a “gate-keeper” with respect to STAT-3 signaling on CD4+ T cells to implicate *trans* (rather than classical) signaling as its preferential mediator. Our data thereby support emerging evidence for the importance of *trans* IL-6 signaling in the promotion of immune-mediated inflammatory pathology (23), including during the earliest stages of RA.

Next, using a model *ex vivo* system to explore the consequences of human naïve CD4+ T cell exposure to sustained IL-6 signaling, we observed an induced transcriptional programme with striking similarity to a previously described molecular signature discriminatory for early RA (2, 4). An enrichment of transcripts functionally associated with cell survival and proliferation could be readily discerned amongst genes dynamically regulated as a consequence of IL-6/sIL-6R pre-exposure at pathophysiological concentrations, and a correspondingly enhanced downstream proliferative capacity was confirmed, as well as an increased capacity for Th1 differentiation. In our model, active removal of cytokine prior to polyclonal TCR activation tested the possibility that this transcriptional imprint—rather than the ongoing presence of IL-6 during stimulation itself—influenced downstream effector function. Our findings indicate that chronic cytokine “priming” of naïve CD4+ T cells in the circulation, which frequently precedes the clinical onset of RA (24), could indeed programme increased proliferative capacity and propensity for Th1-skewing upon subsequent antigen encounter, even when this occurs in tissue where IL-6 may no longer dominate. The parallel observation of increased cell-cycle-commitment amongst circulating CD4+ T cells of untreated RA patients, together with their enhanced IFN-γ production in response to *ex vivo* polyclonal stimulation, appears consistent with our model of cytokine-priming. Contrasting reported roles of IL-6 in guiding CD4+ T cell differentiation—including in relation to Th1/Th17 balance (27, 28), —may instead depend upon continuous availability of the cytokine during TCR stimulation and the presence of other mediators such as TGF-β (29), neither of which were applicable in our “reductionist” experiments.

Approaches for targeting IL-6 that form current RA management guidelines (30, 31) draw primarily upon its long-established credentials as a pro-inflammatory mediator of established disease (32). An increasingly sophisticated appreciation of its complex biology (7), together with growing evidence for its uniquely important role in early disease (1, 33) now warrants a reappraisal of therapeutic strategy. Our work may inform this process. For example, notwithstanding any putative evolutionary advantage conferred by cytokine “priming” of naïve CD4+ T cells in the context of infection (34), even temporary IL-6 signal-blockade during the preclinical phase of RA—conceivably tailored to target the *trans* pathway specifically (35), —could reverse transcriptional imprinting in a time-critical manner and favorably augment subsequent disease progression in an identifiable subgroup of patients. Whilst speculative, this hypothesis is already testable in the clinic, with potential benefits for patients and health economies alike.

## Ethics Statement

The study was approved by the Newcastle and North Tyneside 2 Research Ethics Committee (reference 12/NE/0251) and all patients provided full written informed consent prior to participating in the research.

## Author Contributions

LR conducted the laboratory work and analysis and drafted the manuscript. NM and CL contributed to laboratory work and analysis. AA contributed to laboratory work, analysis, and study design. NN and AS contributed to the analysis. PE and JI contributed to the study's conception and design. RC and AP conceived and designed the study. All authors contributed to, and approved the final version of the manuscript.

### Conflict of Interest Statement

AP, JI, and AA are recipients of investigator-initiated research grants awarded by Pfizer and administered by Newcastle University. The remaining authors declare that the research was conducted in the absence of any commercial or financial relationships that could be construed as a potential conflict of interest.
