# Supplementary material for: IL-6 Mediated Transcriptional Programming of Naïve CD4+ T Cells in Early Rheumatoid Arthritis Drives Dysregulated Effector Function
Source: Front Immunol. 2019 Jul 3;10:1535. doi: 10.3389/fimmu.2019.01535 (PMC6618050; doi:10.3389/fimmu.2019.01535)
Supplement: Supplementary file 1 [file Table_1.DOCX]

Ridgley et al

**IL-6 mediated transcriptional programming of naïve CD4+ T cells in early rheumatoid arthritis drives dysregulated effector function**.

**SUPPLEMENTARY FIGURES**

***Supplementary Figure 1****. Gating strategy for IL-6R and gp130 expression. Doublets were excluded by SSC-A versus SSC-W and lymphocytes gated based on SSC-A versus FSC-A. CD3 positive cells were gated and from this population CD4 versus either gp130 or IL-6R was examined. gp130 or IL-6R positivity was determined by quadrant gates set based on the expression within CD3 positive, CD4 negative cells using a contour plot with level 5%. From CD3 positive cells CD4 positive cells were identified and the CD4+ T-cell sub-populations subsequently gated based on CD45RA versus CD62L with true naïve CD4+ T-cells CD45RA+ CD62L+, central memory CD4+ T-cells CD45RA- CD62L+ and effector memory cells CD45RA- CD62L-The positive IL-6R and gp130 quadrant gates were then copied onto the relevant CD4+ T-cell sub-populations.*

***Supplementary Figure 2.*** *Surface IL-6R expression (****A****) and surface gp130 expression (****B****) was determined in true naïve, central memory and effector memory CD4+ T-cells in the peripheral blood of patients with early arthritis using flow cytometry. N=26. Non-parametric analysis of variance (Friedman’s) with Dunn’s posthoc pairwise analyses, *p<0.01, **p<0.001, ***p<0.0001.*

**SUPPLEMENTARY TABLES**

***Supplementary Table 1*.** *List of genes differentially expressed in naïve CD4+ T-cells 6 hours following exposure to 0.5ng/ml IL-6 and equimolar sIL-6R compared to 6 hours untreated naïve CD4+ T-cells. Differentially expressed genes were identified post multiple test correction by cross-sectional analysis of untreated and IL-6 exposed naïve CD4+ T-cells following 6 hours exposure to 0.5ng/ml IL-6 using moderated paired t-test with fold change over 1.5 and corrected p value cut off of <0.05.* *N=3.*

| Illumina ID | Ensembl ID | Gene | logFC | P.Value | adj.P.Value |
| --- | --- | --- | --- | --- | --- |
| ILMN_1737314 | ENSG00000113916 | BCL6 | 2.55 | 1.52E-15 | 1.56E-11 |
| ILMN_2302757 | ENSG00000090920 | FCGBP | -2.44 | 5.58E-12 | 5.75E-09 |
| ILMN_1808811 | ENSG00000064932 | SBNO2 | 2.36 | 1.28E-16 | 2.64E-12 |
| ILMN_1781001 | ENSG00000184557 | SOCS3 | 2.25 | 3.84E-09 | 1.18E-06 |
| ILMN_1694432 | ENSG00000182809 | CRIP2 | 2.20 | 9.34E-15 | 4.71E-11 |
| ILMN_1710514 | ENSG00000069399 | BCL3 | 2.10 | 2.18E-13 | 5.00E-10 |
| ILMN_1793990 | ENSG00000115738 | ID2 | 2.02 | 1.20E-10 | 6.84E-08 |
| ILMN_2078592 | ENSG00000111863 | C6orf105 | 1.94 | 1.50E-10 | 8.12E-08 |
| ILMN_1721626 | ENSG00000150347 | ARID5B | 1.83 | 6.73E-11 | 4.33E-08 |
| ILMN_1712431 | ENSG00000179715 | FAM113B | 1.82 | 1.55E-12 | 2.28E-09 |
| ILMN_2086095 | ENSG00000115738 | ID2 | 1.81 | 3.50E-10 | 1.68E-07 |
| ILMN_1710124 | ENSG00000170293 | CMTM8 | 1.73 | 1.14E-14 | 4.71E-11 |
| ILMN_1864900 |  | MIAT | 1.71 | 1.43E-08 | 3.38E-06 |
| ILMN_1741133 | ENSG00000239672 | NME1 | 1.71 | 3.93E-10 | 1.84E-07 |
| ILMN_2359287 | ENSG00000091409 | ITGA6 | 1.63 | 5.04E-12 | 5.75E-09 |
| ILMN_1815023 | ENSG00000137193 | PIM1 | 1.61 | 2.77E-12 | 3.35E-09 |
| ILMN_1772521 | ENSG00000120254 | MTHFD1L | 1.60 | 4.77E-14 | 1.40E-10 |
| ILMN_1731224 | ENSG00000138496 | PARP9 | 1.58 | 3.69E-09 | 1.15E-06 |
| ILMN_2367753 | ENSG00000058668 | ATP2B4 | 1.57 | 6.96E-15 | 4.71E-11 |
| ILMN_1780349 | ENSG00000116690 | PRG4 | 1.54 | 2.90E-13 | 5.96E-10 |
| ILMN_1680579 | ENSG00000058668 | ATP2B4 | 1.54 | 1.87E-14 | 6.43E-11 |
| ILMN_2405521 | ENSG00000065911 | MTHFD2 | 1.53 | 4.19E-10 | 1.92E-07 |
| ILMN_1696699 | ENSG00000111879 | FAM184A | 1.53 | 8.34E-10 | 3.18E-07 |
| ILMN_1668822 | ENSG00000156127 | BATF | 1.48 | 1.00E-10 | 6.07E-08 |
| ILMN_1784256 | ENSG00000166503 | HDGFRP3 | 1.44 | 1.28E-07 | 1.85E-05 |
| ILMN_1710075 | ENSG00000182118 | FAM89A | 1.44 | 1.77E-12 | 2.28E-09 |
| ILMN_1718984 | ENSG00000090920 | FCGBP | -1.43 | 1.96E-08 | 4.34E-06 |
| ILMN_2110908 | ENSG00000136997 | MYC | 1.42 | 2.30E-08 | 4.93E-06 |
| ILMN_1779353 | ENSG00000091127 | PUS7 | 1.41 | 3.59E-09 | 1.14E-06 |
| ILMN_2285817 | ENSG00000182118 | FAM89A | 1.40 | 1.00E-11 | 8.62E-09 |
| ILMN_1784709 | ENSG00000113552 | GNPDA1 | 1.40 | 4.45E-11 | 3.06E-08 |
| ILMN_3306997 | ENSG00000037897 | METTL1 | 1.38 | 1.69E-09 | 6.23E-07 |
| ILMN_2053527 | ENSG00000138496 | PARP9 | 1.36 | 1.50E-08 | 3.47E-06 |
| ILMN_2048591 | ENSG00000173114 | LRRN3 | -1.35 | 1.74E-12 | 2.28E-09 |
| ILMN_1773760 | ENSG00000128050 | PAICS | 1.35 | 7.45E-11 | 4.65E-08 |
| ILMN_1725417 | ENSG00000184613 | NELL2 | -1.33 | 2.81E-10 | 1.41E-07 |
| ILMN_3249748 | ENSG00000134333 | LDHA | 1.32 | 1.65E-12 | 2.28E-09 |
| ILMN_2392546 | ENSG00000128050 | PAICS | 1.32 | 1.83E-11 | 1.34E-08 |
| ILMN_1754538 | ENSG00000122378 | C10orf58 | 1.30 | 1.08E-11 | 8.87E-09 |
| ILMN_2402640 | ENSG00000134765 | DSC1 | -1.29 | 3.22E-08 | 6.31E-06 |
| ILMN_1833858 |  | AF038185 | 1.29 | 8.45E-12 | 7.91E-09 |
| ILMN_1806023 | ENSG00000177606 | JUN | 1.28 | 1.76E-05 | 1.01E-03 |
| ILMN_1815190 | ENSG00000037897 | METTL1 | 1.28 | 6.49E-08 | 1.12E-05 |
| ILMN_1652677 | ENSG00000182118 | FAM89A | 1.26 | 2.23E-09 | 7.79E-07 |
| ILMN_1658628 | ENSG00000258512 | C14orf72 | 1.26 | 6.21E-12 | 6.09E-09 |
| ILMN_2376403 | ENSG00000157514 | TSC22D3 | -1.25 | 4.55E-07 | 5.36E-05 |
| ILMN_1680618 | ENSG00000136997 | MYC | 1.24 | 7.31E-08 | 1.22E-05 |
| ILMN_1664265 | ENSG00000146904 | EPHA1 | -1.23 | 5.51E-09 | 1.49E-06 |
| ILMN_1674706 | ENSG00000065911 | MTHFD2 | 1.23 | 5.08E-08 | 9.35E-06 |
| ILMN_1773650 | ENSG00000173114 | LRRN3 | -1.23 | 3.50E-05 | 1.73E-03 |
| ILMN_1807106 | ENSG00000134333 | LDHA | 1.22 | 2.15E-13 | 5.00E-10 |
| ILMN_1748124 | ENSG00000157514 | TSC22D3 | -1.22 | 6.03E-07 | 6.61E-05 |
| ILMN_1724533 | ENSG00000154589 | LY96 | 1.19 | 1.39E-10 | 7.72E-08 |
| ILMN_1682792 | ENSG00000112578 | BYSL | 1.19 | 2.15E-07 | 2.90E-05 |
| ILMN_1811592 | ENSG00000107863 | ARHGAP21 | -1.18 | 9.70E-07 | 9.74E-05 |
| ILMN_1801307 | ENSG00000121858 | TNFSF10 | 1.18 | 5.36E-09 | 1.47E-06 |
| ILMN_1663035 | ENSG00000072310 | SREBF1 | 1.18 | 3.26E-09 | 1.05E-06 |
| ILMN_1655611 | ENSG00000182463 | TSHZ2 | 1.16 | 1.87E-08 | 4.22E-06 |
| ILMN_2255579 | ENSG00000172794 | RAB37 | -1.16 | 1.90E-08 | 4.26E-06 |
| ILMN_1783806 | ENSG00000047579 | DTNBP1 | 1.15 | 3.83E-08 | 7.38E-06 |
| ILMN_1771949 | ENSG00000141384 | TAF4B | 1.15 | 1.10E-06 | 1.09E-04 |
| ILMN_2338197 | ENSG00000140471 | LINS | 1.14 | 1.59E-09 | 5.94E-07 |
| ILMN_2299045 | ENSG00000138756 | BMP2K | 1.14 | 6.03E-10 | 2.48E-07 |
| ILMN_1749009 | ENSG00000076043 | REXO2 | 1.13 | 6.62E-13 | 1.14E-09 |
| ILMN_1767253 | ENSG00000052749 | RRP12 | 1.13 | 1.16E-06 | 1.13E-04 |
| ILMN_1783285 | ENSG00000171793 | CTPS | 1.13 | 5.06E-09 | 1.43E-06 |
| ILMN_1742230 | ENSG00000198604 | BAZ1A | 1.13 | 5.42E-12 | 5.75E-09 |
| ILMN_1676515 | ENSG00000106348 | IMPDH1 | 1.13 | 5.61E-08 | 1.01E-05 |
| ILMN_1707748 | ENSG00000198355 | PIM3 | 1.13 | 4.65E-09 | 1.33E-06 |
| ILMN_1901616 |  |  | 1.13 | 1.28E-07 | 1.85E-05 |
| ILMN_2391419 | ENSG00000054654 | SYNE2 | 1.13 | 6.79E-10 | 2.74E-07 |
| ILMN_1719695 | ENSG00000144802 | NFKBIZ | 1.12 | 6.76E-06 | 4.61E-04 |
| ILMN_1659077 | ENSG00000102245 | CD40LG | -1.12 | 1.12E-06 | 1.10E-04 |
| ILMN_1713384 | ENSG00000227164 | TSHZ2 | 1.12 | 7.27E-08 | 1.22E-05 |
| ILMN_1655077 | ENSG00000057657 | PRDM1 | 1.12 | 4.39E-05 | 2.07E-03 |
| ILMN_3240586 | ENSG00000179598 | PLD6 | 1.11 | 4.79E-10 | 2.10E-07 |
| ILMN_1710756 | ENSG00000074800 | ENO1 | 1.11 | 1.63E-10 | 8.61E-08 |
| ILMN_1653856 | ENSG00000154127 | UBASH3B | 1.10 | 2.42E-10 | 1.25E-07 |
| ILMN_1778723 | ENSG00000160593 | AMICA1 | -1.10 | 7.14E-10 | 2.83E-07 |
| ILMN_1689329 | ENSG00000099194 | SCD | 1.10 | 1.15E-05 | 7.11E-04 |
| ILMN_3266606 | ENSG00000236044 | FABP5 | 1.09 | 9.77E-06 | 6.27E-04 |
| ILMN_1748591 | ENSG00000115758 | ODC1 | 1.09 | 1.13E-06 | 1.11E-04 |
| ILMN_1658327 | ENSG00000198604 | BAZ1A | 1.09 | 1.74E-03 | 3.81E-02 |
| ILMN_2415144 | ENSG00000135899 | SP110 | 1.08 | 9.01E-12 | 8.07E-09 |
| ILMN_1702691 | ENSG00000118503 | TNFAIP3 | -1.08 | 2.62E-06 | 2.13E-04 |
| ILMN_1789338 | ENSG00000120896 | SORBS3 | -1.07 | 4.64E-09 | 1.33E-06 |
| ILMN_1682799 | ENSG00000138134 | STAMBPL1 | 1.07 | 1.33E-11 | 1.06E-08 |
| ILMN_1732296 | ENSG00000117318 | ID3 | -1.07 | 1.92E-06 | 1.69E-04 |
| ILMN_1666453 | ENSG00000104375 | STK3 | 1.07 | 1.22E-06 | 1.16E-04 |
| ILMN_1765326 | ENSG00000077044 | DGKD | -1.06 | 2.97E-08 | 5.89E-06 |
| ILMN_1735461 | ENSG00000165732 | DDX21 | 1.06 | 4.38E-07 | 5.19E-05 |
| ILMN_1801246 | ENSG00000185885 | IFITM1 | 1.06 | 3.51E-13 | 6.57E-10 |
| ILMN_1813455 | ENSG00000135899 | SP110 | 1.06 | 4.70E-10 | 2.10E-07 |
| ILMN_2234873 | ENSG00000011052 | NME1 | 1.05 | 9.02E-08 | 1.42E-05 |
| ILMN_2371700 | ENSG00000213397 | HAUS7 | 1.05 | 2.95E-10 | 1.45E-07 |
| ILMN_1726547 | ENSG00000197442 | MAP3K5 | 1.05 | 6.74E-07 | 7.31E-05 |
| ILMN_1821352 | ENSG00000256862 | BX096603 | -1.05 | 6.80E-07 | 7.34E-05 |
| ILMN_1787815 | ENSG00000101255 | TRIB3 | 1.05 | 8.59E-08 | 1.37E-05 |
| ILMN_1810992 | ENSG00000084774 | CAD | 1.04 | 4.87E-06 | 3.54E-04 |
| ILMN_1767556 | ENSG00000165507 | C10orf10 | 1.04 | 7.65E-07 | 7.95E-05 |
| ILMN_1803941 | ENSG00000121749 | TBC1D15 | 1.04 | 4.82E-11 | 3.20E-08 |
| ILMN_2184184 | ENSG00000135046 | ANXA1 | -1.03 | 3.33E-08 | 6.47E-06 |
| ILMN_1756071 | ENSG00000140545 | MFGE8 | -1.03 | 1.20E-06 | 1.15E-04 |
| ILMN_1748258 |  | LINS1 | 1.03 | 4.89E-08 | 9.16E-06 |
| ILMN_1651574 | ENSG00000165272 | AQP3 | -1.03 | 5.99E-06 | 4.25E-04 |
| ILMN_2168217 | ENSG00000169508 | GPR183 | -1.03 | 4.39E-09 | 1.29E-06 |
| ILMN_1813338 | ENSG00000089692 | LAG3 | 1.03 | 6.97E-07 | 7.47E-05 |
| ILMN_1704554 | ENSG00000235505 | LOC643733 | 1.02 | 2.13E-08 | 4.62E-06 |
| ILMN_1770692 | ENSG00000138442 | WDR12 | 1.02 | 2.27E-07 | 3.02E-05 |
| ILMN_1661439 | ENSG00000223654 | FLOT1 | 1.02 | 1.93E-06 | 1.69E-04 |
| ILMN_3249435 | ENSG00000154127 | UBASH3B | 1.01 | 6.77E-08 | 1.16E-05 |
| ILMN_3299520 | ENSG00000166501 | PRKCB | -1.01 | 3.14E-07 | 3.97E-05 |
| ILMN_1748283 | ENSG00000102096 | PIM2 | 1.01 | 9.16E-07 | 9.31E-05 |
| ILMN_1755024 | ENSG00000143466 | IKBKE | -1.00 | 9.47E-07 | 9.56E-05 |
| ILMN_1752478 | ENSG00000162496 | DHRS3 | -1.00 | 2.07E-06 | 1.76E-04 |
| ILMN_2086077 | ENSG00000171223 | JUNB | 0.99 | 5.45E-07 | 6.24E-05 |
| ILMN_1659888 | ENSG00000173457 | PPP1R14B | 0.99 | 1.05E-07 | 1.62E-05 |
| ILMN_1798204 | ENSG00000103522 | IL21R | 0.99 | 1.54E-05 | 9.03E-04 |
| ILMN_3245907 | ENSG00000256586 | FAM160B2 | 0.99 | 8.19E-08 | 1.33E-05 |
| ILMN_1800602 | ENSG00000115271 | GCA | 0.98 | 1.86E-09 | 6.71E-07 |
| ILMN_1750401 | ENSG00000178927 | C17orf62 | 0.98 | 2.04E-07 | 2.78E-05 |
| ILMN_1737298 | ENSG00000168906 | MAT2A | 0.98 | 5.63E-08 | 1.01E-05 |
| ILMN_2053415 | ENSG00000130164 | LDLR | 0.98 | 2.08E-05 | 1.16E-03 |
| ILMN_1754894 | ENSG00000143110 | C1orf162 | -0.98 | 2.90E-08 | 5.79E-06 |
| ILMN_1798172 | ENSG00000196497 | IPO4 | 0.98 | 3.16E-06 | 2.50E-04 |
| ILMN_1673352 | ENSG00000185201 | IFITM2 | 0.98 | 5.43E-10 | 2.28E-07 |
| ILMN_1701875 | ENSG00000159840 | ZYX | -0.98 | 5.18E-09 | 1.44E-06 |
| ILMN_1777519 | ENSG00000139626 | ITGB7 | -0.97 | 4.15E-09 | 1.24E-06 |
| ILMN_1708004 |  | AY262164 | -0.97 | 7.55E-07 | 7.89E-05 |
| ILMN_1714567 | ENSG00000124942 | AHNAK | -0.97 | 1.39E-07 | 1.98E-05 |
| ILMN_1798706 | ENSG00000169508 | GPR183 | -0.97 | 2.63E-09 | 8.89E-07 |
| ILMN_2132809 | ENSG00000104728 | ARHGEF10 | 0.96 | 1.62E-08 | 3.70E-06 |
| ILMN_2410986 | ENSG00000168610 | STAT3 | 0.96 | 7.99E-07 | 8.26E-05 |
| ILMN_1812278 | ENSG00000122224 | LY9 | -0.96 | 6.76E-06 | 4.61E-04 |
| ILMN_2093343 | ENSG00000145287 | PLAC8 | 0.96 | 2.62E-09 | 8.89E-07 |
| ILMN_1670256 | ENSG00000152256 | PDK1 | 0.95 | 3.75E-06 | 2.89E-04 |
| ILMN_1705570 | ENSG00000099284 | H2AFY2 | -0.94 | 9.92E-07 | 9.92E-05 |
| ILMN_2045729 | ENSG00000138442 | WDR12 | 0.94 | 3.95E-05 | 1.90E-03 |
| ILMN_1743397 | ENSG00000184886 | PIGW | 0.93 | 9.76E-09 | 2.49E-06 |
| ILMN_1695763 | ENSG00000065485 | PDIA5 | 0.93 | 2.38E-08 | 5.05E-06 |
| ILMN_1659725 | ENSG00000077348 | EXOSC5 | 0.93 | 7.27E-09 | 1.92E-06 |
| ILMN_1812995 | ENSG00000135047 | CTSL1 | -0.93 | 2.13E-06 | 1.81E-04 |
| ILMN_2199389 | ENSG00000114812 | VIPR1 | -0.93 | 1.34E-06 | 1.25E-04 |
| ILMN_3247723 | ENSG00000048162 | NOP16 | 0.93 | 1.44E-06 | 1.32E-04 |
| ILMN_2390609 | ENSG00000151150 | ANK3 | 0.93 | 7.32E-07 | 7.79E-05 |
| ILMN_1789596 |  | ETV6 | 0.93 | 4.24E-08 | 8.08E-06 |
| ILMN_1776435 | ENSG00000185477 | GPRIN3 | 0.93 | 5.35E-04 | 1.51E-02 |
| ILMN_1715603 | ENSG00000110944 | IL23A | -0.93 | 7.34E-07 | 7.79E-05 |
| ILMN_1811148 | ENSG00000138756 | BMP2K | 0.93 | 8.05E-08 | 1.32E-05 |
| ILMN_1704055 | ENSG00000048162 | NOP16 | 0.92 | 1.86E-06 | 1.66E-04 |
| ILMN_1803988 | ENSG00000143384 | MCL1 | 0.92 | 8.10E-10 | 3.15E-07 |
| ILMN_3221432 | ENSG00000116690 | PRG4 | 0.92 | 1.10E-07 | 1.69E-05 |
| ILMN_2379560 | ENSG00000081377 | CDC14B | -0.92 | 2.15E-09 | 7.65E-07 |
| ILMN_1673991 | ENSG00000138363 | ATIC | 0.92 | 3.33E-11 | 2.36E-08 |
| ILMN_2106902 | ENSG00000053254 | FOXN3 | -0.92 | 1.04E-10 | 6.15E-08 |
| ILMN_1785202 | ENSG00000138378 | STAT4 | -0.92 | 1.06E-08 | 2.65E-06 |
| ILMN_1739428 | ENSG00000119922 | IFIT2 | -0.92 | 1.86E-07 | 2.58E-05 |
| ILMN_1757074 | ENSG00000242616 | GNG10 | 0.92 | 3.88E-06 | 2.98E-04 |
| ILMN_1731418 | ENSG00000135899 | SP110 | 0.91 | 1.68E-11 | 1.28E-08 |
| ILMN_1716815 | ENSG00000079385 | CEACAM1 | 0.91 | 2.27E-03 | 4.66E-02 |
| ILMN_2381899 | ENSG00000123240 | OPTN | -0.91 | 1.71E-06 | 1.55E-04 |
| ILMN_2115669 | ENSG00000168758 | SEMA4C | -0.91 | 4.09E-06 | 3.12E-04 |
| ILMN_2374036 | ENSG00000135047 | CTSL1 | -0.91 | 3.61E-04 | 1.11E-02 |
| ILMN_1730284 | ENSG00000134765 | DSC1 | -0.91 | 4.61E-06 | 3.43E-04 |
| ILMN_1663113 | ENSG00000100304 | TTLL12 | 0.91 | 5.62E-07 | 6.33E-05 |
| ILMN_1724422 | ENSG00000188404 | SELL | 0.91 | 3.32E-07 | 4.15E-05 |
| ILMN_1709683 | ENSG00000101265 | RASSF2 | -0.91 | 7.01E-08 | 1.19E-05 |
| ILMN_1673252 | ENSG00000106305 | AIMP2 | 0.91 | 5.03E-08 | 9.34E-06 |
| ILMN_1806908 | ENSG00000166501 | PRKCB | -0.90 | 2.68E-06 | 2.16E-04 |
| ILMN_1771800 |  | PRKCA | -0.90 | 3.22E-09 | 1.05E-06 |
| ILMN_1759232 | ENSG00000169047 | IRS1 | 0.90 | 1.96E-04 | 6.95E-03 |
| ILMN_1755862 | ENSG00000178921 | PFAS | 0.90 | 1.04E-06 | 1.03E-04 |
| ILMN_1662905 | ENSG00000243678 | NME1 | 0.90 | 1.47E-08 | 3.44E-06 |
| ILMN_1770641 | ENSG00000146021 | KLHL3 | -0.89 | 2.68E-08 | 5.46E-06 |
| ILMN_1728298 | ENSG00000188322 | SBK1 | -0.89 | 4.39E-06 | 3.32E-04 |
| ILMN_1695962 | ENSG00000146828 | SLC12A9 | 0.89 | 2.59E-07 | 3.37E-05 |
| ILMN_1692517 | ENSG00000140263 | SORD | 0.88 | 7.66E-08 | 1.27E-05 |
| ILMN_2267488 | ENSG00000091409 | ITGA6 | 0.88 | 1.44E-06 | 1.32E-04 |
| ILMN_1718672 | ENSG00000145912 | NHP2 | 0.88 | 5.26E-10 | 2.26E-07 |
| ILMN_1664167 | ENSG00000197498 | RPF2 | 0.88 | 2.16E-06 | 1.81E-04 |
| ILMN_1686555 | ENSG00000010810 | FYN | -0.88 | 1.29E-08 | 3.12E-06 |
| ILMN_1707695 | ENSG00000185745 | IFIT1 | -0.88 | 2.42E-04 | 8.17E-03 |
| ILMN_1731518 | ENSG00000179598 | PLD6 | 0.88 | 2.43E-08 | 5.05E-06 |
| ILMN_1782938 | ENSG00000112394 | SLC16A10 | -0.88 | 8.33E-08 | 1.34E-05 |
| ILMN_1677765 | ENSG00000157193 | LRP8 | 0.87 | 1.84E-07 | 2.56E-05 |
| ILMN_1763129 | ENSG00000179958 | DCTPP1 | 0.87 | 1.16E-07 | 1.75E-05 |
| ILMN_2306540 | ENSG00000160191 | PDE9A | -0.87 | 1.67E-06 | 1.52E-04 |
| ILMN_1784780 | ENSG00000079432 | CIC | 0.87 | 4.27E-07 | 5.08E-05 |
| ILMN_1664859 | ENSG00000111728 | ST8SIA1 | 0.87 | 1.09E-08 | 2.70E-06 |
| ILMN_2173004 | ENSG00000166128 | RAB8B | -0.87 | 3.21E-09 | 1.05E-06 |
| ILMN_2222234 | ENSG00000123131 | PRDX4 | 0.87 | 3.53E-06 | 2.74E-04 |
| ILMN_1672022 | ENSG00000116106 | EPHA4 | -0.87 | 3.94E-07 | 4.77E-05 |
| ILMN_1655311 | ENSG00000189227 | C15orf61 | 0.86 | 5.66E-09 | 1.51E-06 |
| ILMN_2401978 | ENSG00000168610 | STAT3 | 0.86 | 5.31E-05 | 2.41E-03 |
| ILMN_1748883 | ENSG00000129355 | CDKN2D | 0.86 | 2.80E-06 | 2.24E-04 |
| ILMN_1727617 | ENSG00000088930 | XRN2 | 0.86 | 1.13E-07 | 1.73E-05 |
| ILMN_1669624 | ENSG00000140471 | LINS | 0.86 | 2.02E-04 | 7.08E-03 |
| ILMN_2336781 | ENSG00000112096 | SOD2 | 0.86 | 2.64E-07 | 3.42E-05 |
| ILMN_1746408 | ENSG00000167470 | MIDN | 0.86 | 4.37E-04 | 1.29E-02 |
| ILMN_1660754 |  | C13orf25 v_1 | 0.85 | 2.06E-06 | 1.76E-04 |
| ILMN_3236694 | ENSG00000182021 | LOC100133920 | 0.85 | 3.49E-07 | 4.31E-05 |
| ILMN_1736940 | ENSG00000165704 | HPRT1 | 0.85 | 3.03E-07 | 3.85E-05 |
| ILMN_1710937 | ENSG00000163565 | IFI16 | 0.85 | 7.94E-06 | 5.31E-04 |
| ILMN_1770768 | ENSG00000117090 | SLAMF1 | -0.85 | 8.01E-04 | 2.07E-02 |
| ILMN_1651496 | ENSG00000158373 | HIST1H2BD | -0.85 | 2.53E-04 | 8.49E-03 |
| ILMN_1802971 | ENSG00000256586 | FAM160B2 | 0.85 | 2.42E-05 | 1.29E-03 |
| ILMN_2415157 | ENSG00000196843 | ARID5A | 0.85 | 5.82E-07 | 6.48E-05 |
| ILMN_1737611 | ENSG00000139190 | VAMP1 | -0.85 | 4.16E-09 | 1.24E-06 |
| ILMN_1774077 | ENSG00000162645 | GBP2 | 0.85 | 3.22E-06 | 2.53E-04 |
| ILMN_1663916 | ENSG00000123329 | ARHGAP9 | -0.85 | 4.77E-08 | 9.01E-06 |
| ILMN_1683859 | ENSG00000139514 | SLC7A1 | 0.85 | 5.84E-07 | 6.48E-05 |
| ILMN_1692295 | ENSG00000136286 | MYO1G | -0.84 | 9.69E-06 | 6.27E-04 |
| ILMN_1653026 | ENSG00000145287 | PLAC8 | 0.84 | 5.79E-08 | 1.03E-05 |
| ILMN_3238058 | ENSG00000152127 | LOC151162 | -0.84 | 2.51E-08 | 5.16E-06 |
| ILMN_1790461 | ENSG00000137288 | C6orf125 | 0.84 | 5.85E-07 | 6.48E-05 |
| ILMN_1657810 | ENSG00000164088 | PPM1M | -0.84 | 2.53E-07 | 3.32E-05 |
| ILMN_1777881 | ENSG00000048140 | TSPAN17 | 0.84 | 3.21E-07 | 4.03E-05 |
| ILMN_2056975 | ENSG00000165704 | HPRT1 | 0.84 | 2.10E-08 | 4.61E-06 |
| ILMN_1720771 | ENSG00000135604 | STX11 | 0.84 | 6.49E-05 | 2.84E-03 |
| ILMN_1766010 | ENSG00000134684 | YARS | 0.84 | 2.15E-06 | 1.81E-04 |
| ILMN_1814917 | ENSG00000065717 | TLE2 | -0.84 | 5.11E-07 | 5.88E-05 |
| ILMN_2344373 | ENSG00000013364 | MVP | 0.84 | 8.51E-07 | 8.72E-05 |
| ILMN_1656378 | ENSG00000152465 | NMT2 | -0.83 | 3.78E-05 | 1.84E-03 |
| ILMN_2154115 | ENSG00000125637 | PSD4 | -0.83 | 8.06E-06 | 5.35E-04 |
| ILMN_2154052 | ENSG00000119537 | KDSR | 0.83 | 9.81E-09 | 2.49E-06 |
| ILMN_2208903 | ENSG00000169442 | CD52 | -0.83 | 7.92E-08 | 1.31E-05 |
| ILMN_2365544 | ENSG00000145912 | NHP2 | 0.83 | 8.73E-09 | 2.28E-06 |
| ILMN_2156172 | ENSG00000159399 | HK2 | 0.83 | 2.17E-04 | 7.49E-03 |
| ILMN_1729453 | ENSG00000011105 | TSPAN9 | 0.83 | 1.25E-07 | 1.84E-05 |
| ILMN_1898124 | ENSG00000232956 | C7orf40 | 0.83 | 5.14E-08 | 9.36E-06 |
| ILMN_3237981 | ENSG00000172508 | CARNS1 | -0.83 | 4.59E-06 | 3.43E-04 |
| ILMN_2383305 | ENSG00000160818 | GPATCH4 | 0.83 | 2.04E-06 | 1.75E-04 |
| ILMN_2328986 | ENSG00000072310 | SREBF1 | 0.82 | 7.55E-07 | 7.89E-05 |
| ILMN_2403247 | ENSG00000153551 | CMTM7 | 0.82 | 1.14E-06 | 1.11E-04 |
| ILMN_1733956 | ENSG00000196305 | IARS | 0.82 | 1.77E-07 | 2.48E-05 |
| ILMN_1796210 | ENSG00000148840 | PPRC1 | 0.82 | 6.72E-06 | 4.61E-04 |
| ILMN_1693452 | ENSG00000197093 | GAL3ST4 | -0.82 | 1.28E-05 | 7.81E-04 |
| ILMN_2300664 | ENSG00000100346 | CACNA1I | -0.82 | 2.53E-04 | 8.49E-03 |
| ILMN_1815168 | ENSG00000122986 | HVCN1 | -0.82 | 1.89E-05 | 1.07E-03 |
| ILMN_1694589 | ENSG00000170915 | PAQR8 | -0.81 | 4.61E-06 | 3.43E-04 |
| ILMN_1788283 | ENSG00000103187 | COTL1 | 0.81 | 4.78E-04 | 1.38E-02 |
| ILMN_3289508 |  | CR604709 | -0.81 | 1.17E-07 | 1.75E-05 |
| ILMN_1767658 | ENSG00000147576 | RRS1 | 0.81 | 4.89E-06 | 3.55E-04 |
| ILMN_1802151 | ENSG00000021762 | OSBPL5 | 0.81 | 1.45E-03 | 3.27E-02 |
| ILMN_1705116 | ENSG00000137266 | SLC22A23 | -0.80 | 2.41E-05 | 1.29E-03 |
| ILMN_2258816 | ENSG00000164483 | SAMD3 | -0.80 | 3.34E-07 | 4.15E-05 |
| ILMN_1723971 | ENSG00000112759 | SLC29A1 | 0.80 | 3.39E-05 | 1.69E-03 |
| ILMN_1725992 | ENSG00000217128 | RAPGEF6 | 0.80 | 4.71E-06 | 3.46E-04 |
| ILMN_1713744 | ENSG00000227051 | DKFZp761F2014 | -0.80 | 8.73E-05 | 3.65E-03 |
| ILMN_2249920 | ENSG00000010810 | FYN | -0.80 | 2.56E-06 | 2.08E-04 |
| ILMN_1676822 | ENSG00000119147 | C2orf40 | -0.79 | 5.57E-07 | 6.30E-05 |
| ILMN_3237627 | ENSG00000253882 | FAM115C | 0.79 | 4.97E-05 | 2.29E-03 |
| ILMN_1665559 | ENSG00000123374 | CDK2 | 0.79 | 2.66E-06 | 2.15E-04 |
| ILMN_1672661 | ENSG00000135899 | SP110 | 0.79 | 1.83E-06 | 1.64E-04 |
| ILMN_1802646 | ENSG00000106123 | EPHB6 | -0.79 | 1.41E-04 | 5.31E-03 |
| ILMN_1692938 | ENSG00000135069 | PSAT1 | 0.79 | 6.25E-04 | 1.72E-02 |
| ILMN_1740487 | ENSG00000153551 | CMTM7 | 0.78 | 6.78E-05 | 2.94E-03 |
| ILMN_2232177 | ENSG00000072110 | ACTN1 | -0.78 | 5.99E-08 | 1.05E-05 |
| ILMN_2399016 | ENSG00000129270 | MMP28 | -0.78 | 2.48E-05 | 1.31E-03 |
| ILMN_1682930 | ENSG00000213445 | SIPA1 | -0.78 | 1.88E-07 | 2.58E-05 |
| ILMN_3249406 | ENSG00000135763 | URB2 | 0.78 | 4.70E-06 | 3.46E-04 |
| ILMN_1805902 | ENSG00000139610 | CELA1 | -0.78 | 1.19E-06 | 1.14E-04 |
| ILMN_1792681 | ENSG00000110104 | CCDC86 | 0.78 | 6.20E-04 | 1.71E-02 |
| ILMN_1723486 | ENSG00000159399 | HK2 | 0.78 | 2.66E-05 | 1.39E-03 |
| ILMN_1786612 | ENSG00000248988 | PSME2 | 0.77 | 1.00E-05 | 6.36E-04 |
| ILMN_1695422 | ENSG00000115053 | NCL | 0.77 | 3.86E-05 | 1.87E-03 |
| ILMN_3231390 | ENSG00000145912 | NHP2 | 0.77 | 1.58E-07 | 2.22E-05 |
| ILMN_1766657 | ENSG00000148175 | STOM | 0.77 | 4.99E-06 | 3.59E-04 |
| ILMN_1726928 | ENSG00000204219 | TCEA3 | -0.77 | 4.62E-05 | 2.17E-03 |
| ILMN_2324056 | ENSG00000163938 | GNL3 | 0.77 | 7.81E-05 | 3.30E-03 |
| ILMN_1785095 | ENSG00000171130 | ATP6V0E2 | -0.77 | 1.30E-04 | 4.95E-03 |
| ILMN_1696601 | ENSG00000231116 | VARS | 0.77 | 3.30E-06 | 2.58E-04 |
| ILMN_2060115 | ENSG00000137642 | SORL1 | -0.76 | 1.45E-05 | 8.59E-04 |
| ILMN_3247781 | ENSG00000229450 | AK097701 | 0.76 | 4.82E-06 | 3.53E-04 |
| ILMN_1719286 | ENSG00000064601 | CTSA | 0.76 | 2.98E-04 | 9.60E-03 |
| ILMN_1806818 | ENSG00000112118 | MCM3 | 0.76 | 2.31E-06 | 1.91E-04 |
| ILMN_1762262 | ENSG00000171033 | PKIA | -0.76 | 3.81E-07 | 4.65E-05 |
| ILMN_1812926 | ENSG00000163297 | ANTXR2 | -0.76 | 5.72E-06 | 4.10E-04 |
| ILMN_3240321 | ENSG00000181026 | AEN | 0.76 | 3.96E-07 | 4.77E-05 |
| ILMN_3247998 | ENSG00000035720 | STAP1 | -0.76 | 1.67E-05 | 9.66E-04 |
| ILMN_1760449 | ENSG00000064201 | TSPAN32 | -0.75 | 3.04E-04 | 9.74E-03 |
| ILMN_2368530 | ENSG00000008517 | IL32 | 0.75 | 2.33E-04 | 7.93E-03 |
| ILMN_3226807 | ENSG00000145912 | NHP2 | 0.75 | 3.82E-07 | 4.65E-05 |
| ILMN_1656593 | ENSG00000154027 | AK5 | -0.75 | 1.19E-04 | 4.62E-03 |
| ILMN_1671800 |  | CRNDE | 0.75 | 8.79E-04 | 2.24E-02 |
| ILMN_2098446 | ENSG00000141682 | PMAIP1 | 0.75 | 1.98E-04 | 6.98E-03 |
| ILMN_1806106 | ENSG00000163938 | GNL3 | 0.75 | 6.43E-06 | 4.48E-04 |
| ILMN_2320888 | ENSG00000121966 | CXCR4 | -0.75 | 8.17E-04 | 2.11E-02 |
| ILMN_3229467 | ENSG00000123179 | EBPL | 0.75 | 3.50E-06 | 2.73E-04 |
| ILMN_1757370 | ENSG00000166311 | SMPD1 | -0.75 | 1.71E-05 | 9.84E-04 |
| ILMN_2403889 | ENSG00000100462 | PRMT5 | 0.75 | 8.61E-06 | 5.67E-04 |
| ILMN_1674302 | ENSG00000128059 | PPAT | 0.74 | 3.81E-05 | 1.85E-03 |
| ILMN_1716563 | ENSG00000166501 | PRKCB | -0.74 | 2.37E-05 | 1.28E-03 |
| ILMN_1754579 | ENSG00000054654 | SYNE2 | 0.74 | 1.10E-05 | 6.84E-04 |
| ILMN_2070896 | ENSG00000204217 | BMPR2 | -0.74 | 8.85E-05 | 3.67E-03 |
| ILMN_1658399 | ENSG00000139187 | KLRG1 | -0.74 | 3.88E-04 | 1.17E-02 |
| ILMN_1665601 | ENSG00000176946 | THAP4 | 0.74 | 5.95E-07 | 6.55E-05 |
| ILMN_1733690 | ENSG00000118507 | AKAP7 | -0.74 | 5.02E-07 | 5.84E-05 |
| ILMN_1796508 | ENSG00000184428 | TOP1MT | 0.74 | 2.81E-04 | 9.13E-03 |
| ILMN_2352303 | ENSG00000101265 | RASSF2 | -0.74 | 6.70E-05 | 2.92E-03 |
| ILMN_1661337 | ENSG00000116649 | SRM | 0.74 | 6.07E-06 | 4.28E-04 |
| ILMN_1807074 | ENSG00000240972 | MIF | 0.74 | 1.10E-08 | 2.70E-06 |
| ILMN_1751656 | ENSG00000172059 | KLF11 | 0.74 | 4.38E-05 | 2.07E-03 |
| ILMN_1703471 | ENSG00000118217 | ATF6 | 0.74 | 3.20E-05 | 1.62E-03 |
| ILMN_3305304 | ENSG00000106628 | POLD2 | 0.74 | 8.02E-07 | 8.26E-05 |
| ILMN_1759097 | ENSG00000213190 | MLLT11 | -0.73 | 2.09E-07 | 2.84E-05 |
| ILMN_1674402 | ENSG00000165071 | TMEM71 | -0.73 | 2.80E-07 | 3.60E-05 |
| ILMN_2203891 | ENSG00000101665 | SMAD7 | -0.73 | 8.05E-05 | 3.38E-03 |
| ILMN_2387712 | ENSG00000154027 | AK5 | -0.73 | 1.26E-07 | 1.84E-05 |
| ILMN_1698934 | ENSG00000153551 | CMTM7 | 0.73 | 2.70E-05 | 1.40E-03 |
| ILMN_1665483 | ENSG00000080608 | KIAA0020 | 0.73 | 1.65E-05 | 9.57E-04 |
| ILMN_2203896 | ENSG00000101665 | SMAD7 | -0.73 | 2.12E-04 | 7.36E-03 |
| ILMN_1768127 | ENSG00000117395 | EBNA1BP2 | 0.73 | 5.14E-05 | 2.35E-03 |
| ILMN_1659158 | ENSG00000164483 | SAMD3 | -0.73 | 2.07E-05 | 1.15E-03 |
| ILMN_2308849 | ENSG00000179820 | MYADM | -0.73 | 1.25E-07 | 1.84E-05 |
| ILMN_1729161 | ENSG00000148400 | NOTCH1 | 0.73 | 9.36E-04 | 2.36E-02 |
| ILMN_1703327 | ENSG00000140368 | PSTPIP1 | -0.73 | 2.29E-05 | 1.24E-03 |
| ILMN_2129015 | ENSG00000172493 | AFF1 | 0.73 | 6.69E-06 | 4.61E-04 |
| ILMN_1713124 | ENSG00000196139 | AKR1C3 | -0.73 | 4.41E-04 | 1.30E-02 |
| ILMN_1803423 | ENSG00000129675 | ARHGEF6 | -0.73 | 1.30E-06 | 1.21E-04 |
| ILMN_2184640 | ENSG00000166197 | NOLC1 | 0.73 | 3.46E-04 | 1.08E-02 |
| ILMN_1689001 | ENSG00000135446 | CDK4 | 0.72 | 5.55E-07 | 6.30E-05 |
| ILMN_1713756 | ENSG00000148672 | GLUD1 | 0.72 | 1.41E-08 | 3.38E-06 |
| ILMN_2392274 | ENSG00000085117 | CD82 | -0.72 | 1.43E-04 | 5.36E-03 |
| ILMN_1673357 | ENSG00000101082 | SLA2 | -0.72 | 3.05E-05 | 1.55E-03 |
| ILMN_3243142 | ENSG00000114166 | KAT2B | -0.72 | 4.32E-06 | 3.28E-04 |
| ILMN_1714364 | ENSG00000169398 | PTK2 | -0.72 | 2.40E-03 | 4.82E-02 |
| ILMN_1712530 | ENSG00000121057 | AKAP1 | 0.72 | 7.28E-05 | 3.11E-03 |
| ILMN_2414399 | ENSG00000239672 | NME1 | 0.72 | 9.34E-05 | 3.80E-03 |
| ILMN_1664034 | ENSG00000198298 | ZNF485 | 0.72 | 8.65E-06 | 5.67E-04 |
| ILMN_1763452 | ENSG00000185862 | EVI2B | -0.72 | 2.72E-08 | 5.50E-06 |
| ILMN_2243912 | ENSG00000172794 | RAB37 | -0.72 | 7.14E-06 | 4.82E-04 |
| ILMN_1744795 | ENSG00000101849 | TBL1X | -0.71 | 4.08E-06 | 3.12E-04 |
| ILMN_2253732 |  | ST8SIA4 | 0.71 | 2.69E-05 | 1.40E-03 |
| ILMN_2382657 | ENSG00000123329 | ARHGAP9 | -0.71 | 3.16E-04 | 1.00E-02 |
| ILMN_1805922 | ENSG00000123179 | EBPL | 0.71 | 2.44E-06 | 2.00E-04 |
| ILMN_3246608 | ENSG00000166582 | CENPV | 0.71 | 9.18E-07 | 9.31E-05 |
| ILMN_2226271 | ENSG00000248099 | INSL3 | 0.71 | 6.40E-05 | 2.81E-03 |
| ILMN_1701243 | ENSG00000107815 | C10orf2 | 0.71 | 1.73E-06 | 1.56E-04 |
| ILMN_1662658 | ENSG00000177192 | PUS1 | 0.71 | 1.22E-07 | 1.82E-05 |
| ILMN_1662578 | ENSG00000106392 | C1GALT1 | -0.71 | 2.34E-05 | 1.26E-03 |
| ILMN_1771627 | ENSG00000108175 | ZMIZ1 | -0.71 | 1.97E-06 | 1.71E-04 |
| ILMN_3304072 | ENSG00000116690 | PRG4 | 0.71 | 1.49E-05 | 8.78E-04 |
| ILMN_1691341 | ENSG00000168685 | IL7R | -0.71 | 5.96E-06 | 4.25E-04 |
| ILMN_1774733 | ENSG00000185338 | SOCS1 | 0.71 | 4.49E-04 | 1.32E-02 |
| ILMN_1688959 | ENSG00000139193 | CD27 | -0.71 | 7.49E-07 | 7.89E-05 |
| ILMN_1798360 | ENSG00000144476 | CXCR7 | -0.71 | 2.53E-05 | 1.33E-03 |
| ILMN_1775734 | ENSG00000095370 | SH2D3C | -0.71 | 1.19E-04 | 4.62E-03 |
| ILMN_1703441 | ENSG00000142684 | ZNF593 | 0.70 | 1.98E-04 | 6.98E-03 |
| ILMN_1676611 | ENSG00000054148 | PHPT1 | 0.70 | 3.40E-05 | 1.69E-03 |
| ILMN_1775708 | ENSG00000059804 | SLC2A3 | 0.70 | 3.90E-04 | 1.17E-02 |
| ILMN_1722634 | ENSG00000104805 | NUCB1 | 0.70 | 2.38E-06 | 1.96E-04 |
| ILMN_1739751 | ENSG00000181045 | SLC26A11 | -0.70 | 3.32E-04 | 1.05E-02 |
| ILMN_1689518 |  | PECAM1 | -0.70 | 5.27E-05 | 2.40E-03 |
| ILMN_1694147 | ENSG00000110060 | PUS3 | 0.70 | 5.33E-05 | 2.42E-03 |
| ILMN_1748093 | ENSG00000079462 | PAFAH1B3 | 0.70 | 1.37E-05 | 8.22E-04 |
| ILMN_2073289 | ENSG00000170873 | MTSS1 | -0.70 | 1.94E-06 | 1.70E-04 |
| ILMN_2058251 | ENSG00000026025 | VIM | -0.70 | 8.20E-04 | 2.11E-02 |
| ILMN_1711608 | ENSG00000145687 | SSBP2 | -0.70 | 2.04E-05 | 1.14E-03 |
| ILMN_1772951 | ENSG00000070526 | ST6GALNAC1 | 0.70 | 1.63E-04 | 6.01E-03 |
| ILMN_1810423 | ENSG00000124787 | RPP40 | 0.70 | 5.55E-05 | 2.50E-03 |
| ILMN_1673711 | ENSG00000096384 | HSP90AB1 | 0.70 | 6.94E-06 | 4.70E-04 |
| ILMN_3306977 | ENSG00000106348 | IMPDH1 | 0.70 | 2.13E-04 | 7.38E-03 |
| ILMN_3282768 | ENSG00000173457 | PPP1R14B | 0.69 | 1.38E-04 | 5.21E-03 |
| ILMN_1795429 | ENSG00000035403 | VCL | -0.69 | 4.48E-04 | 1.31E-02 |
| ILMN_1783149 | ENSG00000107736 | CDH23 | -0.69 | 1.72E-03 | 3.77E-02 |
| ILMN_1736130 | ENSG00000101546 | RBFA | 0.69 | 2.46E-06 | 2.01E-04 |
| ILMN_1787628 | ENSG00000101361 | NOP56 | 0.69 | 3.83E-04 | 1.16E-02 |
| ILMN_1803277 | ENSG00000013364 | MVP | 0.69 | 1.19E-04 | 4.62E-03 |
| ILMN_1800787 | ENSG00000131378 | RFTN1 | 0.69 | 2.43E-08 | 5.05E-06 |
| ILMN_1814282 | ENSG00000181026 | AEN | 0.69 | 5.76E-05 | 2.56E-03 |
| ILMN_1882354 | ENSG00000244459 | AX748388 | -0.69 | 2.76E-04 | 9.02E-03 |
| ILMN_1657446 | ENSG00000135778 | NTPCR | 0.69 | 1.10E-05 | 6.86E-04 |
| ILMN_1742031 | ENSG00000214706 | IFRD2 | 0.69 | 2.12E-04 | 7.36E-03 |
| ILMN_1744113 | ENSG00000163154 | SCNM1 | -0.69 | 1.78E-04 | 6.41E-03 |
| ILMN_2334693 | ENSG00000141562 | NARF | 0.69 | 2.30E-04 | 7.86E-03 |
| ILMN_2411559 | ENSG00000177192 | PUS1 | 0.69 | 2.13E-05 | 1.17E-03 |
| ILMN_2298511 | ENSG00000183336 | BOLA2 | 0.69 | 2.44E-05 | 1.30E-03 |
| ILMN_3248218 |  | SNORD104 | 0.68 | 8.67E-06 | 5.67E-04 |
| ILMN_1764769 | ENSG00000110002 | VWA5A | 0.68 | 9.98E-06 | 6.36E-04 |
| ILMN_1658494 | ENSG00000102760 | C13orf15 | -0.68 | 8.99E-08 | 1.42E-05 |
| ILMN_1705753 | ENSG00000184220 | C3orf26 | 0.68 | 2.76E-05 | 1.42E-03 |
| ILMN_1679483 | ENSG00000104613 | INTS10 | 0.68 | 1.38E-07 | 1.98E-05 |
| ILMN_1787345 | ENSG00000255863 | FKBP11 | 0.68 | 2.41E-07 | 3.18E-05 |
| ILMN_2385278 | ENSG00000119599 | DCAF4 | 0.68 | 2.76E-06 | 2.21E-04 |
| ILMN_1722981 | ENSG00000187554 | TLR5 | 0.68 | 5.02E-05 | 2.31E-03 |
| ILMN_1804419 | ENSG00000118308 | LRMP | -0.68 | 9.10E-05 | 3.74E-03 |
| ILMN_1775498 | ENSG00000185262 | FAM100B | 0.68 | 2.11E-05 | 1.17E-03 |
| ILMN_2387285 | ENSG00000005302 | MSL3 | 0.68 | 6.11E-07 | 6.66E-05 |
| ILMN_1692539 | ENSG00000100092 | SH3BP1 | 0.68 | 6.75E-04 | 1.82E-02 |
| ILMN_2170814 | ENSG00000078081 | LAMP3 | 0.68 | 4.76E-04 | 1.38E-02 |
| ILMN_2330267 | ENSG00000164163 | ABCE1 | 0.68 | 3.19E-06 | 2.52E-04 |
| ILMN_1709237 | ENSG00000120915 | EPHX2 | -0.68 | 9.15E-05 | 3.75E-03 |
| ILMN_3238369 | ENSG00000006459 | JHDM1D | -0.67 | 1.03E-04 | 4.12E-03 |
| ILMN_1712075 | ENSG00000182253 | KIAA0353 | 0.67 | 2.73E-04 | 8.97E-03 |
| ILMN_2052871 | ENSG00000198270 | TMEM116 | -0.67 | 1.24E-06 | 1.17E-04 |
| ILMN_2053103 | ENSG00000138449 | SLC40A1 | -0.67 | 4.07E-05 | 1.95E-03 |
| ILMN_1668996 | ENSG00000108561 | C1QBP | 0.67 | 1.38E-06 | 1.28E-04 |
| ILMN_1806149 | ENSG00000154102 | C16orf74 | -0.67 | 5.34E-04 | 1.51E-02 |
| ILMN_1798459 | ENSG00000243207 | P2RY11 | 0.67 | 3.95E-04 | 1.18E-02 |
| ILMN_1655051 | ENSG00000145002 | FAM86B1 | 0.67 | 2.47E-05 | 1.31E-03 |
| ILMN_1746686 | ENSG00000171453 | POLR1C | 0.67 | 1.27E-06 | 1.20E-04 |
| ILMN_1708619 | ENSG00000085415 | SEH1L | 0.67 | 8.61E-06 | 5.67E-04 |
| ILMN_1723626 | ENSG00000034053 | APBA2 | 0.67 | 9.78E-05 | 3.93E-03 |
| ILMN_2067682 |  | LOC401431 | -0.67 | 6.93E-05 | 2.99E-03 |
| ILMN_2335718 | ENSG00000197451 | HNRNPAB | 0.67 | 2.51E-05 | 1.32E-03 |
| ILMN_1789775 | ENSG00000133316 | WDR74 | 0.67 | 5.93E-06 | 4.24E-04 |
| ILMN_1784352 | ENSG00000136280 | CCM2 | -0.67 | 1.66E-04 | 6.07E-03 |
| ILMN_1763852 | ENSG00000076555 | ACACB | -0.67 | 8.93E-05 | 3.69E-03 |
| ILMN_1802096 | ENSG00000114626 | ABTB1 | -0.66 | 1.05E-03 | 2.56E-02 |
| ILMN_1730999 | ENSG00000047410 | TPR | 0.66 | 1.66E-04 | 6.07E-03 |
| ILMN_1786433 | ENSG00000107949 | BCCIP | 0.66 | 4.63E-05 | 2.17E-03 |
| ILMN_1702806 | ENSG00000115539 | PDCL3 | 0.66 | 2.30E-06 | 1.91E-04 |
| ILMN_1711087 | ENSG00000227184 | EPPK1 | -0.66 | 1.96E-03 | 4.20E-02 |
| ILMN_1710434 | ENSG00000175463 | TBC1D10C | -0.66 | 1.02E-04 | 4.10E-03 |
| ILMN_3306742 | ENSG00000147955 | SIGMAR1 | 0.66 | 8.16E-06 | 5.40E-04 |
| ILMN_1760280 | ENSG00000132661 | NXT1 | 0.66 | 4.78E-07 | 5.60E-05 |
| ILMN_1878885 | ENSG00000211750 | TCRB | -0.66 | 1.50E-04 | 5.58E-03 |
| ILMN_1702501 | ENSG00000071242 | RPS6KA2 | -0.66 | 9.47E-05 | 3.84E-03 |
| ILMN_1695491 | ENSG00000156795 | WDYHV1 | 0.66 | 2.72E-05 | 1.41E-03 |
| ILMN_1793269 | ENSG00000172794 | RAB37 | -0.66 | 1.14E-04 | 4.49E-03 |
| ILMN_1723962 | ENSG00000079257 | LXN | -0.66 | 2.04E-06 | 1.75E-04 |
| ILMN_1663618 | ENSG00000168610 | STAT3 | 0.66 | 4.88E-04 | 1.40E-02 |
| ILMN_1803312 | ENSG00000086189 | DIMT1L | 0.66 | 1.56E-06 | 1.43E-04 |
| ILMN_1691466 | ENSG00000176105 | YES1 | -0.66 | 1.49E-04 | 5.57E-03 |
| ILMN_1782633 | ENSG00000183336 | BOLA2B | 0.66 | 1.90E-05 | 1.07E-03 |
| ILMN_1803824 | ENSG00000188706 | ZDHHC9 | 0.66 | 2.02E-05 | 1.14E-03 |
| ILMN_1797875 | ENSG00000132965 | ALOX5AP | -0.66 | 9.32E-05 | 3.80E-03 |
| ILMN_2154053 | ENSG00000119537 | KDSR | 0.66 | 1.21E-06 | 1.15E-04 |
| ILMN_1805973 | ENSG00000183150 | GPR19 | 0.66 | 1.83E-05 | 1.04E-03 |
| ILMN_1748427 | ENSG00000196793 | ZNF239 | 0.66 | 3.27E-05 | 1.64E-03 |
| ILMN_1698996 | ENSG00000173638 | SLC19A1 | 0.66 | 1.70E-03 | 3.73E-02 |
| ILMN_1699737 | ENSG00000126602 | TRAP1 | 0.65 | 1.77E-04 | 6.38E-03 |
| ILMN_1768470 | ENSG00000114867 | EIF4G1 | 0.65 | 4.96E-06 | 3.58E-04 |
| ILMN_2062620 |  | NMT2 | -0.65 | 1.40E-05 | 8.40E-04 |
| ILMN_1795089 | ENSG00000105122 | RASAL3 | -0.65 | 2.15E-03 | 4.46E-02 |
| ILMN_1733443 | ENSG00000173597 | SULT1B1 | 0.65 | 1.79E-04 | 6.43E-03 |
| ILMN_2264011 | ENSG00000154016 | GRAP | -0.65 | 1.96E-04 | 6.95E-03 |
| ILMN_1683883 | ENSG00000243989 | ACY1 | 0.65 | 3.60E-05 | 1.77E-03 |
| ILMN_1762095 | ENSG00000125247 | TMTC4 | 0.65 | 1.64E-05 | 9.53E-04 |
| ILMN_3219455 | ENSG00000257576 | HSPD1 | 0.65 | 5.56E-05 | 2.50E-03 |
| ILMN_2331636 | ENSG00000132142 | ACACA | 0.65 | 2.24E-06 | 1.87E-04 |
| ILMN_2315979 | ENSG00000256261 | LBH | -0.65 | 2.87E-07 | 3.67E-05 |
| ILMN_2060105 | ENSG00000243207 | P2RY11 | 0.65 | 4.83E-05 | 2.24E-03 |
| ILMN_1676846 | ENSG00000164163 | ABCE1 | 0.65 | 3.50E-05 | 1.73E-03 |
| ILMN_1770719 | ENSG00000132361 | KIAA0664 | 0.65 | 2.03E-05 | 1.14E-03 |
| ILMN_1684306 | ENSG00000196154 | S100A4 | -0.65 | 6.00E-04 | 1.66E-02 |
| ILMN_1658437 | ENSG00000183605 | SFXN4 | 0.65 | 5.11E-07 | 5.88E-05 |
| ILMN_1671257 | ENSG00000130826 | DKC1 | 0.65 | 4.63E-06 | 3.43E-04 |
| ILMN_2205050 | ENSG00000183943 | PRKX | 0.65 | 1.47E-05 | 8.71E-04 |
| ILMN_1733579 | ENSG00000126860 | EVI2A | -0.65 | 4.59E-06 | 3.43E-04 |
| ILMN_1695079 | ENSG00000181896 | ZNF101 | 0.65 | 9.81E-06 | 6.27E-04 |
| ILMN_1800276 | ENSG00000049449 | RCN1 | 0.65 | 3.71E-05 | 1.81E-03 |
| ILMN_1753249 | ENSG00000178105 | DDX10 | 0.64 | 8.93E-06 | 5.82E-04 |
| ILMN_1652082 | ENSG00000102034 | ELF4 | -0.64 | 2.64E-05 | 1.38E-03 |
| ILMN_1807894 | ENSG00000092068 | SLC7A8 | -0.64 | 1.47E-03 | 3.30E-02 |
| ILMN_3290136 | ENSG00000235505 | LOC643733 | 0.64 | 6.88E-04 | 1.85E-02 |
| ILMN_2227195 | ENSG00000139537 | CCDC65 | -0.64 | 1.05E-04 | 4.17E-03 |
| ILMN_1724480 | ENSG00000168646 | AXIN2 | -0.64 | 2.16E-05 | 1.19E-03 |
| ILMN_1749006 | ENSG00000198771 | RCSD1 | -0.64 | 3.20E-05 | 1.62E-03 |
| ILMN_1767219 | ENSG00000171453 | POLR1C | 0.64 | 2.02E-05 | 1.14E-03 |
| ILMN_1763842 | ENSG00000187024 | PTRH1 | 0.64 | 4.15E-05 | 1.98E-03 |
| ILMN_1789095 | ENSG00000204217 | BMPR2 | -0.64 | 2.26E-05 | 1.23E-03 |
| ILMN_1733538 | ENSG00000148908 | RGS10 | -0.64 | 6.11E-08 | 1.07E-05 |
| ILMN_1793410 | ENSG00000172164 | SNTB1 | 0.64 | 6.28E-06 | 4.40E-04 |
| ILMN_2075189 | ENSG00000110660 | SLC35F2 | 0.64 | 1.04E-03 | 2.54E-02 |
| ILMN_1764362 | ENSG00000145220 | LYAR | 0.64 | 2.53E-04 | 8.49E-03 |
| ILMN_1676548 | ENSG00000136261 | BZW2 | 0.64 | 7.00E-04 | 1.87E-02 |
| ILMN_1798657 | ENSG00000177565 | TBL1XR1 | 0.64 | 1.01E-03 | 2.48E-02 |
| ILMN_1657862 | ENSG00000101444 | AHCY | 0.64 | 2.26E-07 | 3.02E-05 |
| ILMN_1774272 | ENSG00000173153 | ESRRA | 0.64 | 7.33E-05 | 3.12E-03 |
| ILMN_1672807 | ENSG00000169239 | CA5B | -0.64 | 1.15E-04 | 4.51E-03 |
| ILMN_1651628 | ENSG00000138190 | EXOC6 | 0.64 | 1.16E-06 | 1.13E-04 |
| ILMN_1662964 | ENSG00000185238 | PRMT3 | 0.64 | 4.64E-06 | 3.43E-04 |
| ILMN_1769158 | ENSG00000063241 | ISOC2 | 0.64 | 1.69E-04 | 6.14E-03 |
| ILMN_2380801 | ENSG00000010810 | FYN | -0.64 | 4.85E-06 | 3.54E-04 |
| ILMN_2347234 | ENSG00000126457 | PRMT1 | 0.63 | 2.24E-05 | 1.22E-03 |
| ILMN_2337974 | ENSG00000171033 | PKIA | -0.63 | 1.92E-06 | 1.69E-04 |
| ILMN_1714433 | ENSG00000175130 | MARCKSL1 | -0.63 | 1.19E-05 | 7.34E-04 |
| ILMN_1798256 | ENSG00000183696 | UPP1 | 0.63 | 1.68E-04 | 6.11E-03 |
| ILMN_1697827 | ENSG00000074370 | ATP2A3 | -0.63 | 9.39E-04 | 2.36E-02 |
| ILMN_2369018 | ENSG00000126860 | EVI2A | -0.63 | 3.66E-06 | 2.84E-04 |
| ILMN_2364272 | ENSG00000139793 | MBNL2 | -0.63 | 2.27E-04 | 7.78E-03 |
| ILMN_2374383 | ENSG00000048140 | TSPAN17 | 0.63 | 2.87E-04 | 9.31E-03 |
| ILMN_2183687 | ENSG00000203896 | LIME1 | -0.63 | 6.27E-05 | 2.77E-03 |
| ILMN_1761138 | ENSG00000148362 | C9orf142 | 0.63 | 1.09E-05 | 6.84E-04 |
| ILMN_1705302 | ENSG00000104870 | FCGRT | -0.63 | 3.54E-04 | 1.09E-02 |
| ILMN_1684040 | ENSG00000172673 | THEMIS | 0.63 | 2.90E-06 | 2.31E-04 |
| ILMN_1794677 | ENSG00000141524 | TMC6 | -0.63 | 1.25E-03 | 2.92E-02 |
| ILMN_1804851 | ENSG00000239789 | MRPS17 | 0.63 | 8.75E-05 | 3.65E-03 |
| ILMN_1784333 | ENSG00000138593 | SECISBP2L | -0.62 | 7.99E-06 | 5.33E-04 |
| ILMN_2103720 | ENSG00000137547 | MRPL15 | 0.62 | 9.71E-06 | 6.27E-04 |
| ILMN_1782459 | ENSG00000091039 | OSBPL8 | -0.62 | 1.11E-05 | 6.89E-04 |
| ILMN_1724708 | ENSG00000134594 | RAB33A | -0.62 | 7.17E-04 | 1.90E-02 |
| ILMN_1716382 | ENSG00000235162 | C12orf75 | -0.62 | 2.76E-04 | 9.01E-03 |
| ILMN_1654398 | ENSG00000143344 | RGL1 | -0.62 | 1.68E-03 | 3.68E-02 |
| ILMN_1680314 | ENSG00000136810 | TXN | 0.62 | 6.40E-05 | 2.81E-03 |
| ILMN_1771801 | ENSG00000089012 | SIRPG | -0.62 | 5.66E-05 | 2.53E-03 |
| ILMN_1733412 |  | NCRNA00260 | 0.62 | 5.94E-05 | 2.62E-03 |
| ILMN_1723235 | ENSG00000141994 | DUS3L | 0.62 | 6.02E-06 | 4.26E-04 |
| ILMN_1761159 | ENSG00000139641 | ESYT1 | -0.62 | 3.48E-05 | 1.73E-03 |
| ILMN_2110252 | ENSG00000107833 | NPM3 | 0.62 | 2.84E-05 | 1.46E-03 |
| ILMN_1739985 | ENSG00000164691 | TAGAP | -0.62 | 2.49E-03 | 4.95E-02 |
| ILMN_1705390 | ENSG00000099910 | KLHL22 | -0.62 | 1.18E-03 | 2.79E-02 |
| ILMN_1792076 | ENSG00000124496 | TRERF1 | -0.62 | 4.66E-05 | 2.18E-03 |
| ILMN_1724789 |  | CD59 | 0.62 | 3.65E-05 | 1.79E-03 |
| ILMN_2366041 | ENSG00000135916 | ITM2C | -0.62 | 5.10E-05 | 2.34E-03 |
| ILMN_2376204 | ENSG00000231314 | LTB | -0.62 | 4.36E-05 | 2.07E-03 |
| ILMN_1776047 | ENSG00000253564 | HSP90AB3P | 0.62 | 9.11E-05 | 3.74E-03 |
| ILMN_1721457 | ENSG00000099901 | RANBP1 | 0.61 | 7.93E-04 | 2.06E-02 |
| ILMN_1689700 | ENSG00000196843 | ARID5A | 0.61 | 1.07E-04 | 4.25E-03 |
| ILMN_1672503 | ENSG00000092964 | DPYSL2 | -0.61 | 9.81E-06 | 6.27E-04 |
| ILMN_1676980 | ENSG00000170873 | MTSS1 | -0.61 | 6.59E-05 | 2.88E-03 |
| ILMN_1781085 | ENSG00000035720 | STAP1 | -0.61 | 1.28E-04 | 4.90E-03 |
| ILMN_3207933 | ENSG00000164163 | ABCE1 | 0.61 | 1.15E-04 | 4.49E-03 |
| ILMN_1660099 | ENSG00000237706 | LOC399940 | -0.61 | 2.66E-04 | 8.80E-03 |
| ILMN_1695576 | ENSG00000143314 | MRPL24 | 0.61 | 1.01E-07 | 1.57E-05 |
| ILMN_2381397 | ENSG00000144381 | HSPD1 | 0.61 | 1.52E-05 | 8.96E-04 |
| ILMN_2350823 | ENSG00000115041 | KCNIP3 | -0.61 | 2.17E-04 | 7.49E-03 |
| ILMN_1810274 | ENSG00000173917 | HOXB2 | -0.61 | 9.27E-05 | 3.79E-03 |
| ILMN_2107184 | ENSG00000163788 | SNRK | -0.61 | 1.30E-05 | 7.86E-04 |
| ILMN_1672120 | ENSG00000186049 | KRT73 | -0.61 | 2.09E-03 | 4.37E-02 |
| ILMN_1792679 | ENSG00000161638 | ITGA5 | -0.61 | 4.81E-04 | 1.39E-02 |
| ILMN_1695432 | ENSG00000128294 | TPST2 | 0.61 | 2.23E-06 | 1.87E-04 |
| ILMN_3229733 | ENSG00000147955 | SIGMAR1 | 0.60 | 3.64E-05 | 1.79E-03 |
| ILMN_2179837 | ENSG00000175334 | BANF1 | 0.60 | 3.12E-05 | 1.58E-03 |
| ILMN_1725105 | ENSG00000124802 | EEF1E1 | 0.60 | 6.83E-06 | 4.64E-04 |
| ILMN_2044832 | ENSG00000101361 | NOP56 | 0.60 | 4.17E-07 | 5.00E-05 |
| ILMN_1710962 | ENSG00000109084 | TMEM97 | 0.60 | 2.28E-03 | 4.66E-02 |
| ILMN_1671854 |  | DKFZp666C094 | -0.60 | 3.62E-04 | 1.11E-02 |
| ILMN_1782538 | ENSG00000026025 | VIM | -0.60 | 1.44E-03 | 3.27E-02 |
| ILMN_3239103 | ENSG00000254813 | LOC340357 | -0.60 | 2.46E-03 | 4.91E-02 |
| ILMN_1735548 | ENSG00000095951 | HIVEP1 | 0.60 | 1.85E-03 | 4.00E-02 |
| ILMN_1750785 | ENSG00000142765 | SYTL1 | -0.60 | 3.13E-05 | 1.59E-03 |
| ILMN_1674009 | ENSG00000007350 | TKTL1 | -0.60 | 1.60E-03 | 3.55E-02 |
| ILMN_2124386 | ENSG00000237441 | RGL2 | -0.60 | 9.76E-05 | 3.93E-03 |
| ILMN_1746252 | ENSG00000106628 | POLD2 | 0.60 | 1.25E-05 | 7.62E-04 |
| ILMN_1682727 | ENSG00000153814 | JAZF1 | -0.60 | 2.75E-04 | 9.01E-03 |
| ILMN_1796235 | ENSG00000141076 | CIRH1A | 0.60 | 8.52E-05 | 3.57E-03 |
| ILMN_1804629 | ENSG00000196511 | TPK1 | -0.60 | 4.32E-05 | 2.06E-03 |
| ILMN_1816244 | ENSG00000152127 | LOC151162 | -0.60 | 1.08E-04 | 4.28E-03 |
| ILMN_2041190 | ENSG00000164251 | F2RL1 | 0.60 | 3.29E-05 | 1.65E-03 |
| ILMN_2405305 | ENSG00000133794 | ARNTL | -0.60 | 1.41E-05 | 8.40E-04 |
| ILMN_1779015 | ENSG00000181444 | ZNF467 | -0.60 | 1.44E-03 | 3.27E-02 |
| ILMN_2383306 | ENSG00000160818 | GPATCH4 | 0.59 | 7.28E-04 | 1.92E-02 |
| ILMN_1656287 | ENSG00000107742 | SPOCK2 | -0.59 | 7.43E-06 | 4.99E-04 |
| ILMN_1748651 | ENSG00000108294 | PSMB3 | 0.59 | 3.95E-05 | 1.90E-03 |
| ILMN_1759818 | ENSG00000137642 | SORL1 | -0.59 | 8.09E-04 | 2.09E-02 |
| ILMN_1654319 | ENSG00000140511 | HAPLN3 | 0.59 | 5.06E-04 | 1.45E-02 |
| ILMN_1680453 | ENSG00000135916 | ITM2C | -0.59 | 1.53E-04 | 5.67E-03 |
| ILMN_1740430 |  | SLC2A4RG | -0.59 | 6.07E-04 | 1.68E-02 |
| ILMN_1725787 | ENSG00000132005 | RFX1 | -0.59 | 2.19E-05 | 1.20E-03 |
| ILMN_2103761 | ENSG00000106829 | TLE4 | -0.59 | 2.45E-05 | 1.30E-03 |
| ILMN_1724139 | ENSG00000152558 | TMEM123 | 0.59 | 1.60E-04 | 5.91E-03 |
| ILMN_1806198 | ENSG00000101842 | VSIG1 | -0.59 | 1.13E-03 | 2.71E-02 |
| ILMN_3193796 |  | LOC100132024 | -0.59 | 5.87E-04 | 1.64E-02 |
| ILMN_3245458 |  | SNORA61 | 0.59 | 6.44E-06 | 4.48E-04 |
| ILMN_3235514 | ENSG00000169508 | GPR183 | -0.59 | 1.89E-04 | 6.75E-03 |
| ILMN_2109156 | ENSG00000099901 | RANBP1 | 0.59 | 9.21E-06 | 5.98E-04 |
| ILMN_1651262 | ENSG00000197451 | HNRNPAB | 0.59 | 4.88E-05 | 2.26E-03 |
| ILMN_1788531 | ENSG00000137078 | SIT1 | -0.59 | 7.70E-04 | 2.01E-02 |
| ILMN_1746618 | ENSG00000182749 | PAQR7 | 0.59 | 3.63E-04 | 1.11E-02 |
| ILMN_1737308 | ENSG00000173221 | GLRX | 0.59 | 2.92E-04 | 9.43E-03 |
| ILMN_2173451 | ENSG00000105220 | GPI | 0.59 | 1.43E-03 | 3.26E-02 |
| ILMN_1774336 | ENSG00000100479 | POLE2 | 0.59 | 3.68E-04 | 1.12E-02 |

***Supplementary Table 2*.** *List of genes differentially expressed in memory CD4+ T-cells 6 hours following exposure to 0.5ng/ml IL-6 and equimolar sIL-6R compared to 6 hours untreated memory CD4+ T-cells. Differentially expressed genes were identified post multiple test correction by cross-sectional analysis of untreated and IL-6 exposed memory CD4+ T-cells following 6 hours exposure to 0.5ng/ml IL-6 using moderated paired t-test with fold change over 1.5 and corrected p value cut off of <0.05.* *N=3.*

| Illumina ID | Ensembl ID | Gene | logFC | P.Value | adj.P.Value |
| --- | --- | --- | --- | --- | --- |
| ILMN_1710514 | ENSG00000069399 | BCL3 | 1.82 | 1.07E-11 | 3.16E-08 |
| ILMN_1808811 | ENSG00000064932 | SBNO2 | 1.79 | 3.65E-13 | 2.90E-09 |
| ILMN_2302757 | ENSG00000090920 | FCGBP | -1.78 | 1.07E-08 | 8.84E-06 |
| ILMN_1737314 | ENSG00000113916 | BCL6 | 1.77 | 3.51E-11 | 9.03E-08 |
| ILMN_1658628 | ENSG00000258512 | C14orf72 | 1.75 | 5.70E-16 | 1.17E-11 |
| ILMN_2078592 | ENSG00000111863 | C6orf105 | 1.61 | 1.07E-08 | 8.84E-06 |
| ILMN_1781001 | ENSG00000184557 | SOCS3 | 1.58 | 3.93E-06 | 1.19E-03 |
| ILMN_1731224 | ENSG00000138496 | PARP9 | 1.55 | 5.22E-09 | 5.97E-06 |
| ILMN_2053527 | ENSG00000138496 | PARP9 | 1.49 | 2.05E-09 | 2.82E-06 |
| ILMN_1773650 | ENSG00000173114 | LRRN3 | -1.38 | 5.94E-06 | 1.57E-03 |
| ILMN_1710124 | ENSG00000170293 | CMTM8 | 1.37 | 7.63E-12 | 2.87E-08 |
| ILMN_1780349 | ENSG00000116690 | PRG4 | 1.36 | 7.11E-12 | 2.87E-08 |
| ILMN_2285817 | ENSG00000182118 | FAM89A | 1.32 | 5.23E-11 | 1.20E-07 |
| ILMN_1741133 | ENSG00000239672 | NME1 | 1.30 | 1.58E-07 | 8.54E-05 |
| ILMN_2110908 | ENSG00000136997 | MYC | 1.29 | 1.46E-07 | 8.18E-05 |
| ILMN_2048591 | ENSG00000173114 | LRRN3 | -1.28 | 8.35E-12 | 2.87E-08 |
| ILMN_2359287 | ENSG00000091409 | ITGA6 | 1.23 | 5.66E-09 | 6.11E-06 |
| ILMN_1663035 | ENSG00000072310 | SREBF1 | 1.22 | 1.43E-09 | 2.26E-06 |
| ILMN_1694432 | ENSG00000182809 | CRIP2 | 1.20 | 4.26E-08 | 3.25E-05 |
| ILMN_2086095 | ENSG00000115738 | ID2 | 1.20 | 1.81E-06 | 6.42E-04 |
| ILMN_1680618 | ENSG00000136997 | MYC | 1.15 | 3.66E-07 | 1.72E-04 |
| ILMN_1772521 | ENSG00000120254 | MTHFD1L | 1.14 | 4.06E-10 | 8.35E-07 |
| ILMN_1710075 | ENSG00000182118 | FAM89A | 1.13 | 8.82E-10 | 1.51E-06 |
| ILMN_3306997 | ENSG00000037897 | METTL1 | 1.12 | 1.47E-07 | 8.18E-05 |
| ILMN_1748283 | ENSG00000102096 | PIM2 | 1.12 | 1.39E-07 | 8.18E-05 |
| ILMN_1813338 | ENSG00000089692 | LAG3 | 1.10 | 1.94E-07 | 1.02E-04 |
| ILMN_1801246 | ENSG00000185885 | IFITM1 | 1.05 | 4.22E-13 | 2.90E-09 |
| ILMN_1652677 | ENSG00000182118 | FAM89A | 1.05 | 1.17E-07 | 7.29E-05 |
| ILMN_1696699 | ENSG00000111879 | FAM184A | 1.04 | 2.24E-06 | 7.57E-04 |
| ILMN_1864900 |  | MIAT | 1.02 | 9.97E-05 | 1.34E-02 |
| ILMN_1756992 | ENSG00000185499 | MUC1 | 1.02 | 1.01E-08 | 8.84E-06 |
| ILMN_1712431 | ENSG00000179715 | FAM113B | 1.02 | 8.69E-07 | 3.58E-04 |
| ILMN_1682792 | ENSG00000112578 | BYSL | 1.02 | 3.58E-06 | 1.10E-03 |
| ILMN_1668822 | ENSG00000156127 | BATF | 1.01 | 4.14E-07 | 1.90E-04 |
| ILMN_1773760 | ENSG00000128050 | PAICS | 1.01 | 5.42E-08 | 3.99E-05 |
| ILMN_1724533 | ENSG00000154589 | LY96 | 1.00 | 7.81E-09 | 7.31E-06 |
| ILMN_1718984 | ENSG00000090920 | FCGBP | -0.99 | 1.50E-05 | 3.28E-03 |
| ILMN_1793990 | ENSG00000115738 | ID2 | 0.99 | 9.71E-05 | 1.32E-02 |
| ILMN_1653856 | ENSG00000154127 | UBASH3B | 0.96 | 5.94E-09 | 6.11E-06 |
| ILMN_1815190 | ENSG00000037897 | METTL1 | 0.95 | 1.33E-05 | 3.08E-03 |
| ILMN_1655611 | ENSG00000182463 | TSHZ2 | 0.94 | 1.23E-06 | 4.77E-04 |
| ILMN_2392546 | ENSG00000128050 | PAICS | 0.92 | 6.77E-08 | 4.65E-05 |
| ILMN_1664859 | ENSG00000111728 | ST8SIA1 | 0.92 | 3.01E-09 | 3.78E-06 |
| ILMN_1673352 | ENSG00000185201 | IFITM2 | 0.91 | 3.12E-09 | 3.78E-06 |
| ILMN_1803988 | ENSG00000143384 | MCL1 | 0.89 | 1.68E-09 | 2.47E-06 |
| ILMN_1674706 | ENSG00000065911 | MTHFD2 | 0.89 | 1.59E-05 | 3.43E-03 |
| ILMN_2226271 | ENSG00000248099 | INSL3 | 0.89 | 2.21E-06 | 7.57E-04 |
| ILMN_1676515 | ENSG00000106348 | IMPDH1 | 0.88 | 5.02E-06 | 1.46E-03 |
| ILMN_1725417 | ENSG00000184613 | NELL2 | -0.88 | 1.71E-06 | 6.23E-04 |
| ILMN_1772951 | ENSG00000070526 | ST6GALNAC1 | 0.86 | 9.53E-06 | 2.34E-03 |
| ILMN_1805973 | ENSG00000183150 | GPR19 | 0.85 | 2.25E-07 | 1.13E-04 |
| ILMN_1783285 | ENSG00000171793 | CTPS | 0.84 | 1.72E-06 | 6.23E-04 |
| ILMN_2405521 | ENSG00000065911 | MTHFD2 | 0.84 | 4.03E-05 | 7.03E-03 |
| ILMN_1721626 | ENSG00000150347 | ARID5B | 0.84 | 1.60E-04 | 1.90E-02 |
| ILMN_3249435 | ENSG00000154127 | UBASH3B | 0.83 | 2.62E-06 | 8.36E-04 |
| ILMN_3266606 | ENSG00000236044 | FABP5 | 0.83 | 3.90E-04 | 3.54E-02 |
| ILMN_1664265 | ENSG00000146904 | EPHA1 | -0.82 | 1.15E-05 | 2.74E-03 |
| ILMN_1729453 | ENSG00000011105 | TSPAN9 | 0.81 | 2.09E-07 | 1.08E-04 |
| ILMN_1779353 | ENSG00000091127 | PUS7 | 0.80 | 8.14E-05 | 1.21E-02 |
| ILMN_2328986 | ENSG00000072310 | SREBF1 | 0.80 | 1.27E-06 | 4.85E-04 |
| ILMN_1815023 | ENSG00000137193 | PIM1 | 0.80 | 9.03E-06 | 2.29E-03 |
| ILMN_2390609 | ENSG00000151150 | ANK3 | 0.79 | 1.07E-05 | 2.59E-03 |
| ILMN_1731418 | ENSG00000135899 | SP110 | 0.79 | 5.96E-10 | 1.12E-06 |
| ILMN_3247723 | ENSG00000048162 | NOP16 | 0.79 | 2.15E-05 | 4.26E-03 |
| ILMN_1677765 | ENSG00000157193 | LRP8 | 0.79 | 1.33E-06 | 4.99E-04 |
| ILMN_1803277 | ENSG00000013364 | MVP | 0.78 | 2.13E-05 | 4.26E-03 |
| ILMN_1749009 | ENSG00000076043 | REXO2 | 0.78 | 7.27E-09 | 7.13E-06 |
| ILMN_1659077 | ENSG00000102245 | CD40LG | -0.77 | 2.67E-04 | 2.83E-02 |
| ILMN_1807106 | ENSG00000134333 | LDHA | 0.77 | 2.08E-08 | 1.65E-05 |
| ILMN_1803824 | ENSG00000188706 | ZDHHC9 | 0.76 | 1.84E-06 | 6.43E-04 |
| ILMN_1659725 | ENSG00000077348 | EXOSC5 | 0.76 | 4.34E-07 | 1.94E-04 |
| ILMN_1767658 | ENSG00000147576 | RRS1 | 0.76 | 1.38E-05 | 3.16E-03 |
| ILMN_1661439 | ENSG00000223654 | FLOT1 | 0.76 | 1.56E-04 | 1.88E-02 |
| ILMN_2336781 | ENSG00000112096 | SOD2 | 0.76 | 2.55E-06 | 8.34E-04 |
| ILMN_2338197 | ENSG00000140471 | LINS | 0.75 | 5.60E-06 | 1.56E-03 |
| ILMN_2329679 | ENSG00000128294 | TPST2 | 0.75 | 1.10E-07 | 7.08E-05 |
| ILMN_3237627 | ENSG00000253882 | FAM115C | 0.75 | 1.09E-04 | 1.43E-02 |
| ILMN_1720771 | ENSG00000135604 | STX11 | 0.75 | 2.78E-04 | 2.91E-02 |
| ILMN_2093343 | ENSG00000145287 | PLAC8 | 0.74 | 4.96E-07 | 2.17E-04 |
| ILMN_1813455 | ENSG00000135899 | SP110 | 0.74 | 7.62E-07 | 3.20E-04 |
| ILMN_1737298 | ENSG00000168906 | MAT2A | 0.74 | 8.80E-06 | 2.27E-03 |
| ILMN_2048011 | ENSG00000111728 | ST8SIA1 | 0.74 | 2.28E-04 | 2.50E-02 |
| ILMN_3305304 | ENSG00000106628 | POLD2 | 0.74 | 7.42E-07 | 3.19E-04 |
| ILMN_3249748 | ENSG00000134333 | LDHA | 0.74 | 9.24E-07 | 3.73E-04 |
| ILMN_3229467 | ENSG00000123179 | EBPL | 0.74 | 4.41E-06 | 1.31E-03 |
| ILMN_1748883 | ENSG00000129355 | CDKN2D | 0.74 | 3.10E-05 | 5.65E-03 |
| ILMN_1783806 | ENSG00000047579 | DTNBP1 | 0.74 | 7.43E-05 | 1.12E-02 |
| ILMN_1695432 | ENSG00000128294 | TPST2 | 0.73 | 6.17E-08 | 4.38E-05 |
| ILMN_2415144 | ENSG00000135899 | SP110 | 0.73 | 7.84E-08 | 5.21E-05 |
| ILMN_1713384 | ENSG00000227164 | TSHZ2 | 0.72 | 9.59E-05 | 1.32E-02 |
| ILMN_1799504 | ENSG00000204979 | MS4A13 | -0.72 | 3.05E-04 | 3.12E-02 |
| ILMN_1833858 |  | AF038185 | 0.72 | 2.90E-06 | 9.06E-04 |
| ILMN_1704055 | ENSG00000048162 | NOP16 | 0.72 | 8.99E-05 | 1.25E-02 |
| ILMN_1693452 | ENSG00000197093 | GAL3ST4 | -0.72 | 8.81E-05 | 1.24E-02 |
| ILMN_1796210 | ENSG00000148840 | PPRC1 | 0.71 | 5.65E-05 | 9.30E-03 |
| ILMN_1812995 | ENSG00000135047 | CTSL1 | -0.71 | 1.18E-04 | 1.52E-02 |
| ILMN_1708004 |  | AY262164 | -0.71 | 9.86E-05 | 1.34E-02 |
| ILMN_2403247 | ENSG00000153551 | CMTM7 | 0.71 | 1.46E-05 | 3.26E-03 |
| ILMN_2255579 | ENSG00000172794 | RAB37 | -0.70 | 9.54E-05 | 1.32E-02 |
| ILMN_1789338 | ENSG00000120896 | SORBS3 | -0.70 | 1.47E-05 | 3.26E-03 |
| ILMN_1661337 | ENSG00000116649 | SRM | 0.69 | 1.72E-05 | 3.59E-03 |
| ILMN_1805922 | ENSG00000123179 | EBPL | 0.69 | 4.71E-06 | 1.38E-03 |
| ILMN_1752478 | ENSG00000162496 | DHRS3 | -0.69 | 4.12E-04 | 3.71E-02 |
| ILMN_1705570 | ENSG00000099284 | H2AFY2 | -0.68 | 1.43E-04 | 1.77E-02 |
| ILMN_1754894 | ENSG00000143110 | C1orf162 | -0.68 | 2.26E-05 | 4.38E-03 |
| ILMN_1698934 | ENSG00000153551 | CMTM7 | 0.67 | 8.28E-05 | 1.22E-02 |
| ILMN_1658437 | ENSG00000183605 | SFXN4 | 0.67 | 2.45E-07 | 1.17E-04 |
| ILMN_1664167 | ENSG00000197498 | RPF2 | 0.67 | 1.28E-04 | 1.63E-02 |
| ILMN_1698019 | ENSG00000100600 | LGMN | 0.67 | 3.64E-05 | 6.41E-03 |
| ILMN_1748093 | ENSG00000079462 | PAFAH1B3 | 0.67 | 2.70E-05 | 5.06E-03 |
| ILMN_1663113 | ENSG00000100304 | TTLL12 | 0.67 | 7.39E-05 | 1.12E-02 |
| ILMN_1710756 | ENSG00000074800 | ENO1 | 0.67 | 5.35E-06 | 1.51E-03 |
| ILMN_1736311 | ENSG00000028277 | POU2F2 | -0.67 | 1.60E-05 | 3.43E-03 |
| ILMN_1670256 | ENSG00000152256 | PDK1 | 0.67 | 4.83E-04 | 4.19E-02 |
| ILMN_2363361 | ENSG00000183605 | SFXN4 | 0.66 | 6.16E-06 | 1.61E-03 |
| ILMN_2199389 | ENSG00000114812 | VIPR1 | -0.66 | 2.20E-04 | 2.45E-02 |
| ILMN_1707748 | ENSG00000198355 | PIM3 | 0.65 | 7.41E-05 | 1.12E-02 |
| ILMN_1807074 | ENSG00000240972 | MIF | 0.65 | 1.37E-07 | 8.18E-05 |
| ILMN_1696601 | ENSG00000231116 | VARS | 0.65 | 4.06E-05 | 7.03E-03 |
| ILMN_1735461 | ENSG00000165732 | DDX21 | 0.65 | 5.73E-04 | 4.80E-02 |
| ILMN_1805750 | ENSG00000142089 | IFITM3 | 0.65 | 3.09E-04 | 3.13E-02 |
| ILMN_1763129 | ENSG00000179958 | DCTPP1 | 0.65 | 2.23E-05 | 4.37E-03 |
| ILMN_2383305 | ENSG00000160818 | GPATCH4 | 0.64 | 8.72E-05 | 1.24E-02 |
| ILMN_1769158 | ENSG00000063241 | ISOC2 | 0.64 | 1.45E-04 | 1.77E-02 |
| ILMN_2415157 | ENSG00000196843 | ARID5A | 0.64 | 5.05E-05 | 8.59E-03 |
| ILMN_2086077 | ENSG00000171223 | JUNB | 0.64 | 3.72E-04 | 3.41E-02 |
| ILMN_2414007 | ENSG00000243678 | NME1 | 0.64 | 5.94E-06 | 1.57E-03 |
| ILMN_1754538 | ENSG00000122378 | C10orf58 | 0.64 | 2.42E-05 | 4.65E-03 |
| ILMN_1701243 | ENSG00000107815 | C10orf2 | 0.63 | 1.31E-05 | 3.06E-03 |
| ILMN_2234873 | ENSG00000011052 | NME1 | 0.63 | 3.10E-04 | 3.13E-02 |
| ILMN_1770641 | ENSG00000146021 | KLHL3 | -0.63 | 1.67E-05 | 3.51E-03 |
| ILMN_1803941 | ENSG00000121749 | TBC1D15 | 0.62 | 2.40E-06 | 7.98E-04 |
| ILMN_1672661 | ENSG00000135899 | SP110 | 0.62 | 7.16E-05 | 1.11E-02 |
| ILMN_1694589 | ENSG00000170915 | PAQR8 | -0.62 | 2.12E-04 | 2.38E-02 |
| ILMN_3238326 | ENSG00000151692 | RNF144A | 0.62 | 5.82E-06 | 1.57E-03 |
| ILMN_1673991 | ENSG00000138363 | ATIC | 0.62 | 2.40E-07 | 1.17E-04 |
| ILMN_1806052 | ENSG00000109103 | UNC119 | -0.62 | 1.74E-04 | 2.04E-02 |
| ILMN_2346836 | ENSG00000176160 | HSF5 | 0.62 | 1.12E-04 | 1.45E-02 |
| ILMN_1724422 | ENSG00000188404 | SELL | 0.62 | 1.48E-04 | 1.80E-02 |
| ILMN_2298511 | ENSG00000183336 | BOLA2 | 0.62 | 1.08E-04 | 1.42E-02 |
| ILMN_1800602 | ENSG00000115271 | GCA | 0.61 | 1.62E-05 | 3.43E-03 |
| ILMN_3240321 | ENSG00000181026 | AEN | 0.61 | 1.44E-05 | 3.26E-03 |
| ILMN_1682799 | ENSG00000138134 | STAMBPL1 | 0.61 | 2.64E-06 | 8.36E-04 |
| ILMN_1801307 | ENSG00000121858 | TNFSF10 | 0.61 | 3.40E-04 | 3.26E-02 |
| ILMN_1803194 | ENSG00000108479 | GALK1 | 0.61 | 6.00E-05 | 9.80E-03 |
| ILMN_1764769 | ENSG00000110002 | VWA5A | 0.60 | 6.05E-05 | 9.80E-03 |
| ILMN_3231390 | ENSG00000145912 | NHP2 | 0.60 | 1.21E-05 | 2.87E-03 |
| ILMN_1710128 | ENSG00000214534 | FAM66B | -0.60 | 5.26E-04 | 4.48E-02 |
| ILMN_1659888 | ENSG00000173457 | PPP1R14B | 0.60 | 2.74E-04 | 2.88E-02 |
| ILMN_2189842 | ENSG00000140988 | SNORA10 | 0.60 | 4.16E-05 | 7.14E-03 |
| ILMN_1770206 | ENSG00000179409 | GEMIN4 | 0.60 | 5.18E-06 | 1.48E-03 |
| ILMN_1695962 | ENSG00000146828 | SLC12A9 | 0.60 | 1.39E-04 | 1.74E-02 |
| ILMN_1765326 | ENSG00000077044 | DGKD | -0.60 | 3.31E-04 | 3.23E-02 |
| ILMN_1673252 | ENSG00000106305 | AIMP2 | 0.59 | 6.81E-05 | 1.07E-02 |
| ILMN_2371700 | ENSG00000213397 | HAUS7 | 0.59 | 2.14E-05 | 4.26E-03 |
| ILMN_2232177 | ENSG00000072110 | ACTN1 | -0.59 | 9.52E-06 | 2.34E-03 |
| ILMN_1653026 | ENSG00000145287 | PLAC8 | 0.59 | 2.88E-05 | 5.29E-03 |
| ILMN_1750401 | ENSG00000178927 | C17orf62 | 0.59 | 4.58E-04 | 4.04E-02 |
| ILMN_1784780 | ENSG00000079432 | CIC | 0.59 | 1.84E-04 | 2.14E-02 |
| ILMN_1767219 | ENSG00000171453 | POLR1C | 0.59 | 7.19E-05 | 1.11E-02 |

***Supplementary Table 3*.** *List of genes differentially expressed in naive CD4+ T-cells 72 hours following exposure to 0.5ng/ml IL-6 and equimolar sIL-6R compared to 72 hours untreated naïve CD4+ T-cells. Differentially expressed genes were identified post multiple test correction by cross-sectional analysis of untreated and IL-6 exposed naïve CD4+ T-cells following 72 hours exposure to 0.5ng/ml IL-6 using moderated paired t-test with fold change over 1.5 and corrected p value cut off of <0.05.* *N=3.*

| Illumina ID | Ensembl ID | Gene | logFC | P.Value | adj.P.Value |
| --- | --- | --- | --- | --- | --- |
| ILMN_2393765 | ENSG00000128322 | IGLL1 | 3.99 | 1.25E-07 | 2.72E-05 |
| ILMN_1652199 | ENSG00000211632 | abParts | 3.46 | 1.11E-08 | 4.07E-06 |
| ILMN_1739508 | ENSG00000241755 | abParts | 3.42 | 2.22E-09 | 1.24E-06 |
| ILMN_2105441 | ENSG00000132465 | IGJ | 2.94 | 6.27E-08 | 1.59E-05 |
| ILMN_1737314 | ENSG00000113916 | BCL6 | 2.75 | 1.60E-16 | 1.10E-12 |
| ILMN_3240375 | ENSG00000242766 | abParts | 2.24 | 8.89E-07 | 1.27E-04 |
| ILMN_2086095 | ENSG00000115738 | ID2 | 2.21 | 2.15E-12 | 3.68E-09 |
| ILMN_1780349 | ENSG00000116690 | PRG4 | 2.13 | 2.14E-17 | 2.21E-13 |
| ILMN_1804601 | ENSG00000211893 | DKFZp686O16217 | 2.10 | 3.78E-07 | 6.59E-05 |
| ILMN_1680274 | ENSG00000239975 | abParts | 2.10 | 8.59E-06 | 7.59E-04 |
| ILMN_1793990 | ENSG00000115738 | ID2 | 1.96 | 2.61E-10 | 2.15E-07 |
| ILMN_1674228 | ENSG00000239571 | abParts | 1.91 | 6.13E-04 | 1.96E-02 |
| ILMN_1710124 | ENSG00000170293 | CMTM8 | 1.85 | 1.66E-15 | 8.54E-12 |
| ILMN_1808811 | ENSG00000064932 | SBNO2 | 1.85 | 1.44E-13 | 2.97E-10 |
| ILMN_1757604 | ENSG00000198467 | TPM2 | -1.80 | 5.13E-12 | 7.54E-09 |
| ILMN_1692938 | ENSG00000135069 | PSAT1 | 1.77 | 5.91E-10 | 4.51E-07 |
| ILMN_1704537 | ENSG00000092621 | PHGDH | 1.72 | 1.00E-10 | 9.36E-08 |
| ILMN_1720373 | ENSG00000103257 | SLC7A5 | 1.70 | 3.79E-09 | 1.86E-06 |
| ILMN_1772521 | ENSG00000120254 | MTHFD1L | 1.68 | 1.24E-14 | 4.24E-11 |
| ILMN_1724533 | ENSG00000154589 | LY96 | 1.67 | 1.74E-14 | 5.12E-11 |
| ILMN_1864900 |  | MIAT | 1.65 | 3.08E-08 | 9.76E-06 |
| ILMN_1833858 |  | AF038185 | 1.64 | 1.10E-14 | 4.24E-11 |
| ILMN_1712431 | ENSG00000179715 | FAM113B | 1.63 | 2.72E-11 | 3.29E-08 |
| ILMN_1767556 | ENSG00000165507 | C10orf10 | 1.61 | 6.11E-11 | 6.62E-08 |
| ILMN_1789196 | ENSG00000198467 | TPM2 | -1.61 | 2.19E-10 | 1.88E-07 |
| ILMN_1677765 | ENSG00000157193 | LRP8 | 1.58 | 1.15E-13 | 2.62E-10 |
| ILMN_1801246 | ENSG00000185885 | IFITM1 | 1.58 | 2.75E-18 | 5.67E-14 |
| ILMN_1741133 | ENSG00000239672 | NME1 | 1.54 | 4.24E-09 | 2.03E-06 |
| ILMN_1812926 | ENSG00000163297 | ANTXR2 | -1.48 | 2.69E-12 | 4.27E-09 |
| ILMN_1662905 | ENSG00000243678 | NME1 | 1.48 | 5.04E-14 | 1.30E-10 |
| ILMN_1732296 | ENSG00000117318 | ID3 | -1.42 | 7.35E-09 | 3.11E-06 |
| ILMN_2405521 | ENSG00000065911 | MTHFD2 | 1.38 | 5.05E-09 | 2.36E-06 |
| ILMN_1676515 | ENSG00000106348 | IMPDH1 | 1.37 | 8.33E-10 | 5.72E-07 |
| ILMN_1781001 | ENSG00000184557 | SOCS3 | 1.36 | 3.74E-05 | 2.33E-03 |
| ILMN_1796013 | ENSG00000183010 | PYCR1 | 1.34 | 1.63E-09 | 9.97E-07 |
| ILMN_1689329 | ENSG00000099194 | SCD | 1.33 | 4.07E-07 | 6.84E-05 |
| ILMN_2184184 | ENSG00000135046 | ANXA1 | -1.31 | 1.50E-10 | 1.34E-07 |
| ILMN_1733443 | ENSG00000173597 | SULT1B1 | 1.31 | 6.92E-10 | 5.09E-07 |
| ILMN_2110908 | ENSG00000136997 | MYC | 1.30 | 1.35E-07 | 2.86E-05 |
| ILMN_1683859 | ENSG00000139514 | SLC7A1 | 1.27 | 8.12E-11 | 8.36E-08 |
| ILMN_1674706 | ENSG00000065911 | MTHFD2 | 1.24 | 4.04E-08 | 1.17E-05 |
| ILMN_1788108 | ENSG00000259040 | TXNDC5 | 1.23 | 8.53E-08 | 2.02E-05 |
| ILMN_1748283 | ENSG00000102096 | PIM2 | 1.23 | 2.05E-08 | 7.00E-06 |
| ILMN_1680618 | ENSG00000136997 | MYC | 1.19 | 1.71E-07 | 3.53E-05 |
| ILMN_1783149 | ENSG00000107736 | CDH23 | -1.19 | 1.32E-06 | 1.74E-04 |
| ILMN_1708004 |  | AY262164 | -1.19 | 1.34E-08 | 4.86E-06 |
| ILMN_1730284 | ENSG00000134765 | DSC1 | -1.17 | 4.30E-08 | 1.20E-05 |
| ILMN_1710075 | ENSG00000182118 | FAM89A | 1.17 | 3.40E-10 | 2.70E-07 |
| ILMN_1807106 | ENSG00000134333 | LDHA | 1.15 | 1.05E-12 | 1.96E-09 |
| ILMN_1682792 | ENSG00000112578 | BYSL | 1.15 | 4.09E-07 | 6.84E-05 |
| ILMN_3305273 | ENSG00000230787 | PSAT1 | 1.15 | 2.47E-05 | 1.71E-03 |
| ILMN_1659077 | ENSG00000102245 | CD40LG | -1.14 | 8.22E-07 | 1.21E-04 |
| ILMN_1731224 | ENSG00000138496 | PARP9 | 1.14 | 2.49E-06 | 2.93E-04 |
| ILMN_1663035 | ENSG00000072310 | SREBF1 | 1.13 | 7.41E-09 | 3.11E-06 |
| ILMN_2414007 | ENSG00000243678 | NME1 | 1.13 | 3.68E-11 | 4.21E-08 |
| ILMN_3249748 | ENSG00000134333 | LDHA | 1.13 | 9.03E-11 | 8.85E-08 |
| ILMN_1721626 | ENSG00000150347 | ARID5B | 1.13 | 1.95E-06 | 2.41E-04 |
| ILMN_2098446 | ENSG00000141682 | PMAIP1 | 1.13 | 3.59E-07 | 6.38E-05 |
| ILMN_1758418 | ENSG00000102524 | TNFSF13B | 1.12 | 4.47E-08 | 1.20E-05 |
| ILMN_2053527 | ENSG00000138496 | PARP9 | 1.09 | 1.17E-06 | 1.59E-04 |
| ILMN_2402640 | ENSG00000134765 | DSC1 | -1.09 | 9.18E-07 | 1.30E-04 |
| ILMN_3202315 | ENSG00000232883 | RPLP0 | 1.09 | 1.33E-11 | 1.71E-08 |
| ILMN_1659888 | ENSG00000173457 | PPP1R14B | 1.08 | 1.88E-08 | 6.58E-06 |
| ILMN_2338197 | ENSG00000140471 | LINS | 1.07 | 6.19E-09 | 2.71E-06 |
| ILMN_2078592 | ENSG00000111863 | C6orf105 | 1.07 | 1.95E-05 | 1.42E-03 |
| ILMN_2234873 | ENSG00000011052 | NME1 | 1.06 | 7.69E-08 | 1.84E-05 |
| ILMN_1800276 | ENSG00000049449 | RCN1 | 1.05 | 5.43E-09 | 2.43E-06 |
| ILMN_1773780 | ENSG00000103254 | FAM173A | 1.05 | 7.22E-08 | 1.75E-05 |
| ILMN_3221432 | ENSG00000116690 | PRG4 | 1.05 | 7.65E-09 | 3.15E-06 |
| ILMN_1657810 | ENSG00000164088 | PPM1M | -1.05 | 2.59E-09 | 1.38E-06 |
| ILMN_2391419 | ENSG00000054654 | SYNE2 | 1.05 | 3.62E-09 | 1.82E-06 |
| ILMN_1714418 | ENSG00000183688 | FAM101B | -1.04 | 7.20E-04 | 2.20E-02 |
| ILMN_1785202 | ENSG00000138378 | STAT4 | -1.04 | 7.25E-10 | 5.15E-07 |
| ILMN_1749009 | ENSG00000076043 | REXO2 | 1.03 | 7.84E-12 | 1.08E-08 |
| ILMN_1748258 |  | LINS1 | 1.03 | 5.01E-08 | 1.32E-05 |
| ILMN_2341724 | ENSG00000164512 | ANKRD55 | -1.03 | 7.24E-06 | 6.71E-04 |
| ILMN_1871233 | ENSG00000211857 | TCRA | -1.03 | 7.80E-06 | 7.11E-04 |
| ILMN_1773760 | ENSG00000128050 | PAICS | 1.02 | 4.40E-08 | 1.20E-05 |
| ILMN_1815023 | ENSG00000137193 | PIM1 | 1.02 | 1.26E-07 | 2.72E-05 |
| ILMN_1652677 | ENSG00000182118 | FAM89A | 1.02 | 2.12E-07 | 4.12E-05 |
| ILMN_2392546 | ENSG00000128050 | PAICS | 1.02 | 9.48E-09 | 3.75E-06 |
| ILMN_1752478 | ENSG00000162496 | DHRS3 | -1.01 | 1.51E-06 | 1.92E-04 |
| ILMN_1779353 | ENSG00000091127 | PUS7 | 1.01 | 2.86E-06 | 3.29E-04 |
| ILMN_1784780 | ENSG00000079432 | CIC | 0.99 | 3.17E-08 | 9.91E-06 |
| ILMN_1683883 | ENSG00000243989 | ACY1 | 0.99 | 2.49E-08 | 8.12E-06 |
| ILMN_1782788 | ENSG00000060138 | CSDA | 0.98 | 6.70E-04 | 2.09E-02 |
| ILMN_2255579 | ENSG00000172794 | RAB37 | -0.98 | 5.09E-07 | 7.99E-05 |
| ILMN_2193233 | ENSG00000170476 | MZB1 | 0.98 | 1.13E-05 | 9.06E-04 |
| ILMN_1730999 | ENSG00000047410 | TPR | 0.98 | 4.35E-07 | 7.17E-05 |
| ILMN_2285817 | ENSG00000182118 | FAM89A | 0.97 | 5.11E-08 | 1.33E-05 |
| ILMN_1770768 | ENSG00000117090 | SLAMF1 | -0.97 | 1.75E-04 | 7.88E-03 |
| ILMN_1705737 | ENSG00000178035 | IMPDH2 | 0.97 | 1.65E-09 | 9.97E-07 |
| ILMN_1653856 | ENSG00000154127 | UBASH3B | 0.97 | 5.23E-09 | 2.39E-06 |
| ILMN_1789095 | ENSG00000204217 | BMPR2 | -0.96 | 1.63E-08 | 5.79E-06 |
| ILMN_1784871 | ENSG00000169710 | FASN | 0.95 | 8.96E-06 | 7.76E-04 |
| ILMN_2066858 | ENSG00000102524 | TNFSF13B | 0.95 | 5.03E-06 | 5.13E-04 |
| ILMN_2045729 | ENSG00000138442 | WDR12 | 0.94 | 3.75E-05 | 2.33E-03 |
| ILMN_3306997 | ENSG00000037897 | METTL1 | 0.94 | 3.58E-06 | 3.94E-04 |
| ILMN_2154115 | ENSG00000125637 | PSD4 | -0.94 | 1.06E-06 | 1.48E-04 |
| ILMN_1673357 | ENSG00000101082 | SLA2 | -0.93 | 4.57E-07 | 7.42E-05 |
| ILMN_1756992 | ENSG00000185499 | MUC1 | 0.93 | 7.18E-08 | 1.75E-05 |
| ILMN_2363361 | ENSG00000183605 | SFXN4 | 0.93 | 1.01E-08 | 3.92E-06 |
| ILMN_1694432 | ENSG00000182809 | CRIP2 | 0.92 | 5.24E-06 | 5.26E-04 |
| ILMN_1766010 | ENSG00000134684 | YARS | 0.91 | 4.35E-07 | 7.17E-05 |
| ILMN_2258816 | ENSG00000164483 | SAMD3 | -0.91 | 3.00E-08 | 9.66E-06 |
| ILMN_1767253 | ENSG00000052749 | RRP12 | 0.91 | 3.91E-05 | 2.40E-03 |
| ILMN_2382657 | ENSG00000123329 | ARHGAP9 | -0.90 | 1.35E-05 | 1.05E-03 |
| ILMN_3272424 | ENSG00000135486 | HNRNPA1 | 0.90 | 2.09E-09 | 1.20E-06 |
| ILMN_1751726 | ENSG00000213553 | RPLP0 | 0.90 | 1.33E-09 | 8.82E-07 |
| ILMN_1815190 | ENSG00000037897 | METTL1 | 0.90 | 2.72E-05 | 1.86E-03 |
| ILMN_1693014 | ENSG00000172216 | CEBPB | 0.90 | 4.97E-07 | 7.99E-05 |
| ILMN_1793410 | ENSG00000172164 | SNTB1 | 0.90 | 1.03E-08 | 3.92E-06 |
| ILMN_1798557 | ENSG00000182230 | FAM153A | -0.90 | 9.12E-07 | 1.30E-04 |
| ILMN_1772951 | ENSG00000070526 | ST6GALNAC1 | 0.90 | 4.47E-06 | 4.65E-04 |
| ILMN_1769926 | ENSG00000113758 | DBN1 | 0.90 | 3.41E-07 | 6.17E-05 |
| ILMN_1694589 | ENSG00000170915 | PAQR8 | -0.89 | 9.47E-07 | 1.33E-04 |
| ILMN_1803941 | ENSG00000121749 | TBC1D15 | 0.89 | 2.05E-09 | 1.20E-06 |
| ILMN_3251341 | ENSG00000167553 | TUBA1C | 0.89 | 4.04E-06 | 4.31E-04 |
| ILMN_2142284 | ENSG00000077713 | SLC25A43 | 0.89 | 3.14E-06 | 3.53E-04 |
| ILMN_1821270 | ENSG00000211848 | TCRA | -0.89 | 1.12E-03 | 3.00E-02 |
| ILMN_1755862 | ENSG00000178921 | PFAS | 0.88 | 1.33E-06 | 1.74E-04 |
| ILMN_1748591 | ENSG00000115758 | ODC1 | 0.88 | 3.55E-05 | 2.24E-03 |
| ILMN_3229467 | ENSG00000123179 | EBPL | 0.88 | 1.91E-07 | 3.80E-05 |
| ILMN_1792681 | ENSG00000110104 | CCDC86 | 0.88 | 1.55E-04 | 7.19E-03 |
| ILMN_1873838 | ENSG00000211599 | IGKC | 0.88 | 1.08E-03 | 2.93E-02 |
| ILMN_1770641 | ENSG00000146021 | KLHL3 | -0.88 | 4.08E-08 | 1.17E-05 |
| ILMN_1710756 | ENSG00000074800 | ENO1 | 0.88 | 3.54E-08 | 1.07E-05 |
| ILMN_1692707 | ENSG00000184924 | C2orf79 | 0.87 | 2.09E-08 | 7.00E-06 |
| ILMN_2298860 | ENSG00000103253 | HAGHL | 0.87 | 4.17E-05 | 2.52E-03 |
| ILMN_1684158 | ENSG00000166123 | GPT2 | 0.87 | 3.87E-06 | 4.19E-04 |
| ILMN_1805922 | ENSG00000123179 | EBPL | 0.87 | 6.19E-08 | 1.59E-05 |
| ILMN_2336609 | ENSG00000137501 | SYTL2 | -0.87 | 2.35E-04 | 9.86E-03 |
| ILMN_1750401 | ENSG00000178927 | C17orf62 | 0.87 | 2.12E-06 | 2.57E-04 |
| ILMN_1682929 | ENSG00000137501 | SYTL2 | -0.86 | 4.62E-05 | 2.76E-03 |
| ILMN_1798947 | ENSG00000164512 | ANKRD55 | -0.86 | 1.36E-04 | 6.38E-03 |
| ILMN_1709237 | ENSG00000120915 | EPHX2 | -0.86 | 2.48E-06 | 2.93E-04 |
| ILMN_1673352 | ENSG00000185201 | IFITM2 | 0.86 | 1.09E-08 | 4.07E-06 |
| ILMN_1806023 | ENSG00000177606 | JUN | 0.86 | 2.03E-03 | 4.54E-02 |
| ILMN_1748093 | ENSG00000079462 | PAFAH1B3 | 0.86 | 4.54E-07 | 7.42E-05 |
| ILMN_1713384 | ENSG00000227164 | TSHZ2 | 0.86 | 8.54E-06 | 7.59E-04 |
| ILMN_3247781 | ENSG00000229450 | AK097701 | 0.86 | 6.31E-07 | 9.41E-05 |
| ILMN_2073289 | ENSG00000170873 | MTSS1 | -0.85 | 4.50E-08 | 1.20E-05 |
| ILMN_1798172 | ENSG00000196497 | IPO4 | 0.85 | 2.75E-05 | 1.87E-03 |
| ILMN_3211463 | ENSG00000135486 | HNRNPA1 | 0.85 | 6.85E-08 | 1.72E-05 |
| ILMN_1676893 | ENSG00000138031 | ADCY3 | 0.85 | 3.72E-07 | 6.55E-05 |
| ILMN_1668822 | ENSG00000156127 | BATF | 0.85 | 8.45E-06 | 7.56E-04 |
| ILMN_1745075 | ENSG00000089157 | RPLP0 | 0.85 | 4.46E-06 | 4.65E-04 |
| ILMN_1770692 | ENSG00000138442 | WDR12 | 0.85 | 6.06E-06 | 5.86E-04 |
| ILMN_1679158 | ENSG00000165591 | FAAH2 | 0.85 | 5.63E-05 | 3.19E-03 |
| ILMN_1787815 | ENSG00000101255 | TRIB3 | 0.85 | 4.23E-06 | 4.50E-04 |
| ILMN_1719649 | ENSG00000196187 | TMEM63A | -0.84 | 1.77E-05 | 1.32E-03 |
| ILMN_1702691 | ENSG00000118503 | TNFAIP3 | -0.84 | 9.98E-05 | 5.06E-03 |
| ILMN_1787345 | ENSG00000255863 | FKBP11 | 0.84 | 2.61E-09 | 1.38E-06 |
| ILMN_1765326 | ENSG00000077044 | DGKD | -0.84 | 2.31E-06 | 2.76E-04 |
| ILMN_1898071 | ENSG00000211884 | TCRA | -0.84 | 3.23E-04 | 1.25E-02 |
| ILMN_1707627 | ENSG00000111669 | TPI1 | 0.84 | 3.51E-07 | 6.28E-05 |
| ILMN_3245879 | ENSG00000248019 | FAM13AOS | -0.83 | 1.30E-06 | 1.73E-04 |
| ILMN_3247723 | ENSG00000048162 | NOP16 | 0.83 | 8.87E-06 | 7.71E-04 |
| ILMN_1668996 | ENSG00000108561 | C1QBP | 0.83 | 2.11E-08 | 7.00E-06 |
| ILMN_1658437 | ENSG00000183605 | SFXN4 | 0.83 | 3.14E-09 | 1.62E-06 |
| ILMN_1700081 | ENSG00000134363 | FST | 0.83 | 2.21E-05 | 1.55E-03 |
| ILMN_1696066 | ENSG00000110619 | CARS | 0.83 | 2.55E-06 | 2.96E-04 |
| ILMN_1800993 | ENSG00000103351 | CLUAP1 | 0.83 | 7.45E-06 | 6.85E-04 |
| ILMN_1673252 | ENSG00000106305 | AIMP2 | 0.83 | 3.01E-07 | 5.53E-05 |
| ILMN_1661439 | ENSG00000223654 | FLOT1 | 0.83 | 4.70E-05 | 2.80E-03 |
| ILMN_1707748 | ENSG00000198355 | PIM3 | 0.83 | 2.17E-06 | 2.61E-04 |
| ILMN_2222234 | ENSG00000123131 | PRDX4 | 0.82 | 8.26E-06 | 7.43E-04 |
| ILMN_1671791 | ENSG00000100889 | PCK2 | 0.82 | 1.09E-06 | 1.50E-04 |
| ILMN_2328986 | ENSG00000072310 | SREBF1 | 0.82 | 8.72E-07 | 1.26E-04 |
| ILMN_3249435 | ENSG00000154127 | UBASH3B | 0.81 | 3.94E-06 | 4.23E-04 |
| ILMN_2367469 | ENSG00000110619 | CARS | 0.81 | 1.74E-06 | 2.17E-04 |
| ILMN_1798706 | ENSG00000169508 | GPR183 | -0.81 | 1.05E-07 | 2.41E-05 |
| ILMN_2128967 | ENSG00000137720 | C11orf1 | 0.81 | 2.55E-07 | 4.81E-05 |
| ILMN_1701930 | ENSG00000114942 | EEF1B2 | 0.81 | 1.15E-07 | 2.61E-05 |
| ILMN_1659206 | ENSG00000131759 | RARA | -0.81 | 3.83E-08 | 1.13E-05 |
| ILMN_2415144 | ENSG00000135899 | SP110 | 0.81 | 9.41E-09 | 3.75E-06 |
| ILMN_3247998 | ENSG00000035720 | STAP1 | -0.81 | 6.04E-06 | 5.86E-04 |
| ILMN_1793201 | ENSG00000103253 | HAGHL | 0.81 | 1.24E-05 | 9.83E-04 |
| ILMN_1763129 | ENSG00000179958 | DCTPP1 | 0.81 | 5.65E-07 | 8.55E-05 |
| ILMN_1746948 | ENSG00000215375 | MYL5 | 0.80 | 1.62E-06 | 2.05E-04 |
| ILMN_1730523 | ENSG00000172366 | FAM195A | 0.80 | 6.64E-06 | 6.31E-04 |
| ILMN_2368530 | ENSG00000008517 | IL32 | 0.80 | 1.05E-04 | 5.25E-03 |
| ILMN_1701243 | ENSG00000107815 | C10orf2 | 0.80 | 1.92E-07 | 3.80E-05 |
| ILMN_1726547 | ENSG00000197442 | MAP3K5 | 0.80 | 5.00E-05 | 2.91E-03 |
| ILMN_1669550 | ENSG00000116670 | MAD2L2 | 0.80 | 3.82E-07 | 6.61E-05 |
| ILMN_1696699 | ENSG00000111879 | FAM184A | 0.80 | 1.10E-04 | 5.37E-03 |
| ILMN_1714741 | ENSG00000254615 | LOC346887 | 0.80 | 5.37E-07 | 8.25E-05 |
| ILMN_1774077 | ENSG00000162645 | GBP2 | 0.80 | 8.59E-06 | 7.59E-04 |
| ILMN_1692271 | ENSG00000136280 | CCM2 | -0.80 | 2.18E-05 | 1.55E-03 |
| ILMN_1693781 | ENSG00000069493 | CLEC2D | -0.79 | 1.50E-04 | 6.96E-03 |
| ILMN_1764769 | ENSG00000110002 | VWA5A | 0.79 | 8.57E-07 | 1.24E-04 |
| ILMN_1760490 | ENSG00000115170 | ACVR1 | -0.79 | 1.93E-06 | 2.39E-04 |
| ILMN_2110252 | ENSG00000107833 | NPM3 | 0.79 | 5.11E-07 | 7.99E-05 |
| ILMN_2410771 | ENSG00000079999 | KEAP1 | 0.79 | 4.06E-07 | 6.84E-05 |
| ILMN_1694268 | ENSG00000144485 | HES6 | 0.79 | 5.35E-05 | 3.04E-03 |
| ILMN_1661359 | ENSG00000204165 | CXorf65 | -0.79 | 5.62E-06 | 5.53E-04 |
| ILMN_1651574 | ENSG00000165272 | AQP3 | -0.79 | 2.58E-04 | 1.05E-02 |
| ILMN_1783285 | ENSG00000171793 | CTPS | 0.78 | 5.83E-06 | 5.72E-04 |
| ILMN_2355042 | ENSG00000103351 | CLUAP1 | 0.78 | 4.09E-07 | 6.84E-05 |
| ILMN_1654518 | ENSG00000077454 | SAP25 | -0.78 | 1.79E-04 | 8.05E-03 |
| ILMN_1788531 | ENSG00000137078 | SIT1 | -0.78 | 2.12E-05 | 1.53E-03 |
| ILMN_1757370 | ENSG00000166311 | SMPD1 | -0.78 | 8.69E-06 | 7.65E-04 |
| ILMN_2327346 | ENSG00000130511 | SSBP4 | 0.78 | 1.37E-06 | 1.78E-04 |
| ILMN_1696601 | ENSG00000231116 | VARS | 0.78 | 2.54E-06 | 2.96E-04 |
| ILMN_1743205 | ENSG00000064687 | ABCA7 | -0.78 | 2.15E-04 | 9.19E-03 |
| ILMN_1789338 | ENSG00000120896 | SORBS3 | -0.77 | 2.90E-06 | 3.30E-04 |
| ILMN_1792538 | ENSG00000173762 | CD7 | 0.77 | 3.91E-06 | 4.22E-04 |
| ILMN_1715947 | ENSG00000220157 | HNRNPA1 | 0.77 | 9.93E-04 | 2.77E-02 |
| ILMN_3226807 | ENSG00000145912 | NHP2 | 0.77 | 2.31E-07 | 4.44E-05 |
| ILMN_1803988 | ENSG00000143384 | MCL1 | 0.77 | 4.17E-08 | 1.18E-05 |
| ILMN_1741032 | ENSG00000138641 | HERC3 | -0.77 | 1.47E-04 | 6.81E-03 |
| ILMN_1702199 | ENSG00000183662 | FAM19A1 | -0.77 | 3.91E-04 | 1.42E-02 |
| ILMN_1720623 | ENSG00000164674 | SYTL3 | -0.77 | 1.06E-05 | 8.79E-04 |
| ILMN_1754538 | ENSG00000122378 | C10orf58 | 0.77 | 1.12E-06 | 1.53E-04 |
| ILMN_1742431 |  | AY358241 | -0.77 | 6.68E-04 | 2.09E-02 |
| ILMN_1703441 | ENSG00000142684 | ZNF593 | 0.77 | 6.72E-05 | 3.72E-03 |
| ILMN_1803277 | ENSG00000013364 | MVP | 0.77 | 2.95E-05 | 1.98E-03 |
| ILMN_1677997 | ENSG00000103495 | MAZ | 0.76 | 4.47E-06 | 4.65E-04 |
| ILMN_1766657 | ENSG00000148175 | STOM | 0.76 | 6.39E-06 | 6.15E-04 |
| ILMN_1661346 | ENSG00000135486 | HNRNPA1 | 0.76 | 1.55E-07 | 3.27E-05 |
| ILMN_1731418 | ENSG00000135899 | SP110 | 0.76 | 1.61E-09 | 9.97E-07 |
| ILMN_2188333 | ENSG00000110848 | CD69 | -0.76 | 6.17E-05 | 3.46E-03 |
| ILMN_1748836 | ENSG00000010361 | FUZ | 0.76 | 6.92E-08 | 1.72E-05 |
| ILMN_1805902 | ENSG00000139610 | CELA1 | -0.75 | 2.11E-06 | 2.57E-04 |
| ILMN_1760556 | ENSG00000117616 | BC041843 | -0.75 | 1.19E-03 | 3.14E-02 |
| ILMN_3307940 | ENSG00000177337 | FLJ35776 | 0.75 | 4.25E-05 | 2.56E-03 |
| ILMN_3235176 | ENSG00000186854 | C2orf89 | -0.75 | 2.96E-06 | 3.35E-04 |
| ILMN_1873034 | ENSG00000229164 | TCRA | -0.74 | 2.34E-04 | 9.83E-03 |
| ILMN_1675878 | ENSG00000244119 | LOC285359 | -0.74 | 1.88E-05 | 1.37E-03 |
| ILMN_2278819 | ENSG00000205268 | PDE7A | -0.74 | 1.22E-03 | 3.17E-02 |
| ILMN_1697363 | ENSG00000101220 | C20orf27 | 0.74 | 1.26E-04 | 6.00E-03 |
| ILMN_2103720 | ENSG00000137547 | MRPL15 | 0.74 | 5.53E-07 | 8.44E-05 |
| ILMN_1691466 | ENSG00000176105 | YES1 | -0.74 | 3.19E-05 | 2.09E-03 |
| ILMN_2410038 | ENSG00000166801 | FAM111A | -0.74 | 1.64E-06 | 2.06E-04 |
| ILMN_2070896 | ENSG00000204217 | BMPR2 | -0.74 | 9.35E-05 | 4.84E-03 |
| ILMN_1805827 | ENSG00000180817 | PPA1 | 0.74 | 1.05E-05 | 8.69E-04 |
| ILMN_1784709 | ENSG00000113552 | GNPDA1 | 0.74 | 1.90E-05 | 1.38E-03 |
| ILMN_1733956 | ENSG00000196305 | IARS | 0.74 | 1.47E-06 | 1.89E-04 |
| ILMN_1727617 | ENSG00000088930 | XRN2 | 0.74 | 2.01E-06 | 2.47E-04 |
| ILMN_1811258 | ENSG00000104856 | RELB | 0.73 | 7.87E-05 | 4.22E-03 |
| ILMN_2168217 | ENSG00000169508 | GPR183 | -0.73 | 3.42E-06 | 3.81E-04 |
| ILMN_2359287 | ENSG00000091409 | ITGA6 | 0.73 | 5.80E-05 | 3.27E-03 |
| ILMN_1776047 | ENSG00000253564 | HSP90AB3P | 0.73 | 7.65E-06 | 7.00E-04 |
| ILMN_1674402 | ENSG00000165071 | TMEM71 | -0.73 | 2.99E-07 | 5.53E-05 |
| ILMN_2249920 | ENSG00000010810 | FYN | -0.73 | 1.08E-05 | 8.87E-04 |
| ILMN_2410772 | ENSG00000079999 | KEAP1 | 0.73 | 6.47E-06 | 6.19E-04 |
| ILMN_2413537 | ENSG00000136929 | HEMGN | -0.73 | 2.62E-04 | 1.07E-02 |
| ILMN_2257015 | ENSG00000231268 | AGER | -0.72 | 8.31E-05 | 4.43E-03 |
| ILMN_1748124 | ENSG00000157514 | TSC22D3 | -0.72 | 9.45E-04 | 2.69E-02 |
| ILMN_3227811 | ENSG00000229887 | HNRNPA1 | 0.72 | 1.14E-05 | 9.12E-04 |
| ILMN_1710962 | ENSG00000109084 | TMEM97 | 0.72 | 3.71E-04 | 1.36E-02 |
| ILMN_2096719 | ENSG00000198873 | GRK5 | -0.72 | 6.46E-07 | 9.57E-05 |
| ILMN_1651628 | ENSG00000138190 | EXOC6 | 0.72 | 1.18E-07 | 2.64E-05 |
| ILMN_1659725 | ENSG00000077348 | EXOSC5 | 0.72 | 1.27E-06 | 1.70E-04 |
| ILMN_3240838 | ENSG00000169100 | SLC25A6 | 0.72 | 8.84E-06 | 7.71E-04 |
| ILMN_1663916 | ENSG00000123329 | ARHGAP9 | -0.72 | 1.18E-06 | 1.59E-04 |
| ILMN_1668345 | ENSG00000184232 | OAF | 0.72 | 9.20E-06 | 7.91E-04 |
| ILMN_3304072 | ENSG00000116690 | PRG4 | 0.71 | 1.35E-05 | 1.05E-03 |
| ILMN_1652185 | ENSG00000077238 | IL4R | -0.71 | 2.15E-05 | 1.54E-03 |
| ILMN_2286870 | ENSG00000141551 | CSNK1D | 0.71 | 9.95E-06 | 8.40E-04 |
| ILMN_2199389 | ENSG00000114812 | VIPR1 | -0.71 | 8.54E-05 | 4.50E-03 |
| ILMN_1810729 | ENSG00000122042 | UBL3 | -0.71 | 2.67E-07 | 5.00E-05 |
| ILMN_1689725 | ENSG00000137818 | RPLP1 | 0.71 | 4.73E-06 | 4.89E-04 |
| ILMN_1807972 | ENSG00000135596 | MICAL1 | -0.71 | 3.10E-05 | 2.05E-03 |
| ILMN_1687958 | ENSG00000177542 | SLC25A22 | 0.71 | 1.06E-04 | 5.25E-03 |
| ILMN_1811468 | ENSG00000177508 | IRX3 | 0.70 | 1.51E-03 | 3.66E-02 |
| ILMN_2067682 |  | LOC401431 | -0.70 | 3.22E-05 | 2.10E-03 |
| ILMN_1784333 | ENSG00000138593 | SECISBP2L | -0.70 | 1.08E-06 | 1.50E-04 |
| ILMN_1795933 | ENSG00000147457 | CHMP7 | -0.70 | 3.45E-05 | 2.19E-03 |
| ILMN_2369580 | ENSG00000103148 | C16orf35 | 0.70 | 9.71E-06 | 8.23E-04 |
| ILMN_1661264 | ENSG00000182199 | SHMT2 | 0.70 | 2.06E-07 | 4.04E-05 |
| ILMN_2215545 | ENSG00000184220 | C3orf26 | 0.70 | 1.75E-04 | 7.88E-03 |
| ILMN_1719205 | ENSG00000105202 | FBL | 0.70 | 9.78E-08 | 2.29E-05 |
| ILMN_1724789 |  | CD59 | 0.70 | 5.39E-06 | 5.34E-04 |
| ILMN_1659158 | ENSG00000164483 | SAMD3 | -0.70 | 3.92E-05 | 2.40E-03 |
| ILMN_1774427 | ENSG00000012822 | CALCOCO1 | -0.70 | 3.55E-06 | 3.93E-04 |
| ILMN_1805973 | ENSG00000183150 | GPR19 | 0.70 | 7.46E-06 | 6.85E-04 |
| ILMN_1725366 | ENSG00000083807 | SLC27A5 | 0.69 | 1.37E-05 | 1.06E-03 |
| ILMN_2070300 | ENSG00000224979 | LSM2 | 0.69 | 1.27E-07 | 2.72E-05 |
| ILMN_1774974 | ENSG00000103351 | CLUAP1 | 0.69 | 2.03E-04 | 8.79E-03 |
| ILMN_2352303 | ENSG00000101265 | RASSF2 | -0.69 | 1.60E-04 | 7.35E-03 |
| ILMN_1696360 | ENSG00000164733 | CTSB | -0.69 | 2.20E-04 | 9.31E-03 |
| ILMN_1764770 | ENSG00000154814 | OXNAD1 | -0.69 | 6.92E-04 | 2.14E-02 |
| ILMN_1763852 | ENSG00000076555 | ACACB | -0.69 | 5.66E-05 | 3.20E-03 |
| ILMN_1718672 | ENSG00000145912 | NHP2 | 0.69 | 1.26E-07 | 2.72E-05 |
| ILMN_1716547 | ENSG00000124357 | NAGK | -0.69 | 3.19E-05 | 2.09E-03 |
| ILMN_2203896 | ENSG00000101665 | SMAD7 | -0.69 | 4.27E-04 | 1.51E-02 |
| ILMN_3236694 | ENSG00000182021 | LOC100133920 | 0.69 | 1.46E-05 | 1.11E-03 |
| ILMN_1786024 | ENSG00000100413 | POLR3H | 0.68 | 5.26E-06 | 5.26E-04 |
| ILMN_1712616 | ENSG00000157343 | C6orf81 | -0.68 | 1.32E-05 | 1.04E-03 |
| ILMN_2059886 | ENSG00000075234 | TTC38 | 0.68 | 3.62E-06 | 3.97E-04 |
| ILMN_1659845 | ENSG00000166398 | KIAA0355 | -0.68 | 3.36E-07 | 6.12E-05 |
| ILMN_1670101 | ENSG00000069493 | CLEC2D | -0.68 | 7.59E-04 | 2.28E-02 |
| ILMN_1669070 | ENSG00000027001 | MIPEP | 0.68 | 1.63E-04 | 7.47E-03 |
| ILMN_1852151 |  | BX110351 | -0.68 | 1.96E-04 | 8.59E-03 |
| ILMN_1712075 | ENSG00000182253 | KIAA0353 | 0.68 | 2.47E-04 | 1.02E-02 |
| ILMN_3205656 | ENSG00000235595 | GAPDH | 0.68 | 3.73E-05 | 2.32E-03 |
| ILMN_1651610 | ENSG00000164056 | SPRY1 | -0.68 | 1.34E-03 | 3.36E-02 |
| ILMN_2365544 | ENSG00000145912 | NHP2 | 0.68 | 5.12E-07 | 7.99E-05 |
| ILMN_1768127 | ENSG00000117395 | EBNA1BP2 | 0.68 | 1.32E-04 | 6.23E-03 |
| ILMN_2324056 | ENSG00000163938 | GNL3 | 0.67 | 4.08E-04 | 1.46E-02 |
| ILMN_1754894 | ENSG00000143110 | C1orf162 | -0.67 | 2.48E-05 | 1.71E-03 |
| ILMN_1737611 | ENSG00000139190 | VAMP1 | -0.67 | 5.00E-07 | 7.99E-05 |
| ILMN_1747759 | ENSG00000109046 | WSB1 | -0.67 | 1.37E-03 | 3.41E-02 |
| ILMN_2097185 | ENSG00000110060 | PUS3 | 0.67 | 1.08E-03 | 2.93E-02 |
| ILMN_1785324 | ENSG00000100714 | MTHFD1 | 0.67 | 2.62E-04 | 1.06E-02 |
| ILMN_1663113 | ENSG00000100304 | TTLL12 | 0.67 | 7.72E-05 | 4.16E-03 |
| ILMN_1714433 | ENSG00000175130 | MARCKSL1 | -0.67 | 5.08E-06 | 5.15E-04 |
| ILMN_1656232 | ENSG00000204677 | FAM153A | -0.67 | 2.78E-04 | 1.12E-02 |
| ILMN_1740265 | ENSG00000097021 | ACOT7 | 0.67 | 2.96E-04 | 1.17E-02 |
| ILMN_2381476 | ENSG00000198513 | ATL1 | -0.66 | 1.04E-04 | 5.22E-03 |
| ILMN_1705570 | ENSG00000099284 | H2AFY2 | -0.66 | 1.89E-04 | 8.35E-03 |
| ILMN_1736752 | ENSG00000165644 | COMTD1 | 0.66 | 9.85E-04 | 2.76E-02 |
| ILMN_1683313 | ENSG00000008513 | ST3GAL1 | -0.66 | 1.67E-04 | 7.62E-03 |
| ILMN_1807074 | ENSG00000240972 | MIF | 0.66 | 1.01E-07 | 2.34E-05 |
| ILMN_1767658 | ENSG00000147576 | RRS1 | 0.66 | 8.97E-05 | 4.69E-03 |
| ILMN_1740170 | ENSG00000250479 | CHCHD10 | 0.66 | 8.09E-06 | 7.31E-04 |
| ILMN_2380801 | ENSG00000010810 | FYN | -0.66 | 2.48E-06 | 2.93E-04 |
| ILMN_1807798 | ENSG00000135390 | ATP5G2 | -0.66 | 1.07E-03 | 2.92E-02 |
| ILMN_1655611 | ENSG00000182463 | TSHZ2 | 0.66 | 2.13E-04 | 9.11E-03 |
| ILMN_1743397 | ENSG00000184886 | PIGW | 0.66 | 7.00E-06 | 6.58E-04 |
| ILMN_1792660 | ENSG00000118200 | CAMSAP1L1 | -0.66 | 1.74E-05 | 1.31E-03 |
| ILMN_3243190 |  | EMR4P | -0.66 | 2.81E-04 | 1.13E-02 |
| ILMN_3283772 | ENSG00000111640 | GAPDH | 0.66 | 1.05E-04 | 5.25E-03 |
| ILMN_2072296 | ENSG00000123975 | CKS2 | 0.66 | 5.92E-04 | 1.92E-02 |
| ILMN_1680774 | ENSG00000170233 | LOC728392 | -0.66 | 1.42E-03 | 3.49E-02 |
| ILMN_1689001 | ENSG00000135446 | CDK4 | 0.66 | 3.16E-06 | 3.53E-04 |
| ILMN_2058251 | ENSG00000026025 | VIM | -0.66 | 1.56E-03 | 3.73E-02 |
| ILMN_2048591 | ENSG00000173114 | LRRN3 | -0.66 | 1.02E-05 | 8.60E-04 |
| ILMN_1728047 | ENSG00000117448 | AKR1A1 | 0.65 | 3.23E-08 | 9.93E-06 |
| ILMN_2347234 | ENSG00000126457 | PRMT1 | 0.65 | 1.42E-05 | 1.09E-03 |
| ILMN_1673711 | ENSG00000096384 | HSP90AB1 | 0.65 | 1.87E-05 | 1.37E-03 |
| ILMN_2290628 |  | IL16 | -0.65 | 1.94E-04 | 8.51E-03 |
| ILMN_1664912 | ENSG00000137070 | IL11RA | -0.65 | 7.40E-04 | 2.24E-02 |
| ILMN_1655935 | ENSG00000121281 | ADCY7 | -0.65 | 7.07E-06 | 6.62E-04 |
| ILMN_2208903 | ENSG00000169442 | CD52 | -0.65 | 6.65E-06 | 6.31E-04 |
| ILMN_1740487 | ENSG00000153551 | CMTM7 | 0.65 | 6.51E-04 | 2.05E-02 |
| ILMN_3305304 | ENSG00000106628 | POLD2 | 0.65 | 6.85E-06 | 6.47E-04 |
| ILMN_2328094 | ENSG00000165617 | DACT1 | -0.65 | 3.89E-05 | 2.40E-03 |
| ILMN_2342033 | ENSG00000158769 | F11R | -0.65 | 2.53E-04 | 1.04E-02 |
| ILMN_1724422 | ENSG00000188404 | SELL | 0.65 | 7.71E-05 | 4.16E-03 |
| ILMN_1662578 | ENSG00000106392 | C1GALT1 | -0.65 | 8.54E-05 | 4.50E-03 |
| ILMN_1772686 | ENSG00000127084 | FGD3 | -0.65 | 1.94E-04 | 8.51E-03 |
| ILMN_2403247 | ENSG00000153551 | CMTM7 | 0.65 | 5.22E-05 | 3.00E-03 |
| ILMN_1786612 | ENSG00000248988 | PSME2 | 0.65 | 1.27E-04 | 6.05E-03 |
| ILMN_1813490 | ENSG00000105255 | FSD1 | 0.65 | 2.46E-04 | 1.02E-02 |
| ILMN_1657862 | ENSG00000101444 | AHCY | 0.65 | 1.83E-07 | 3.73E-05 |
| ILMN_3247639 | ENSG00000246223 | C14orf64 | -0.64 | 9.48E-05 | 4.89E-03 |
| ILMN_1693430 | ENSG00000243678 | NME1 | 0.64 | 4.26E-05 | 2.56E-03 |
| ILMN_1695491 | ENSG00000156795 | WDYHV1 | 0.64 | 3.95E-05 | 2.41E-03 |
| ILMN_2395711 | ENSG00000141480 | ARRB2 | -0.64 | 1.12E-03 | 3.00E-02 |
| ILMN_2264011 | ENSG00000154016 | GRAP | -0.64 | 2.39E-04 | 9.98E-03 |
| ILMN_1780298 | ENSG00000118894 | FAM86A | 0.64 | 1.87E-04 | 8.30E-03 |
| ILMN_1763452 | ENSG00000185862 | EVI2B | -0.64 | 2.47E-07 | 4.71E-05 |
| ILMN_1771084 | ENSG00000005187 | ACSM3 | 0.64 | 2.88E-05 | 1.94E-03 |
| ILMN_1699603 | ENSG00000183048 | MRPL12 | 0.64 | 9.20E-05 | 4.78E-03 |
| ILMN_1695962 | ENSG00000146828 | SLC12A9 | 0.64 | 5.34E-05 | 3.04E-03 |
| ILMN_2358626 | ENSG00000156110 | ADK | 0.64 | 1.71E-07 | 3.53E-05 |
| ILMN_1670256 | ENSG00000152256 | PDK1 | 0.64 | 7.37E-04 | 2.23E-02 |
| ILMN_1684114 |  | LOC286016 | 0.64 | 3.63E-04 | 1.34E-02 |
| ILMN_2043816 | ENSG00000136950 | ARPC5L | 0.64 | 2.84E-05 | 1.92E-03 |
| ILMN_3231390 | ENSG00000145912 | NHP2 | 0.64 | 4.90E-06 | 5.02E-04 |
| ILMN_1695576 | ENSG00000143314 | MRPL24 | 0.64 | 3.82E-08 | 1.13E-05 |
| ILMN_1729170 | ENSG00000143167 | GPA33 | 0.64 | 1.88E-05 | 1.37E-03 |
| ILMN_1777881 | ENSG00000048140 | TSPAN17 | 0.64 | 3.25E-05 | 2.12E-03 |
| ILMN_1880342 | ENSG00000211801 | TCRA | -0.64 | 1.72E-03 | 4.00E-02 |
| ILMN_1674009 | ENSG00000007350 | TKTL1 | -0.64 | 8.99E-04 | 2.58E-02 |
| ILMN_1787762 | ENSG00000119285 | HEATR1 | 0.63 | 1.75E-05 | 1.31E-03 |
| ILMN_1736597 | ENSG00000163931 | TKT | 0.63 | 7.06E-05 | 3.87E-03 |
| ILMN_3255903 |  |  | 0.63 | 1.51E-05 | 1.15E-03 |
| ILMN_1663080 | ENSG00000106003 | LFNG | -0.63 | 5.19E-05 | 2.99E-03 |
| ILMN_2301624 | ENSG00000127603 | MACF1 | -0.63 | 6.50E-04 | 2.05E-02 |
| ILMN_1668649 | ENSG00000136449 | MYCBPAP | -0.63 | 3.08E-04 | 1.20E-02 |
| ILMN_3240586 | ENSG00000179598 | PLD6 | 0.63 | 2.97E-05 | 1.99E-03 |
| ILMN_1765109 | ENSG00000215788 | TNFRSF25 | -0.63 | 4.31E-06 | 4.56E-04 |
| ILMN_2111739 | ENSG00000140400 | MAN2C1 | -0.63 | 5.15E-05 | 2.98E-03 |
| ILMN_2315979 | ENSG00000256261 | LBH | -0.63 | 6.13E-07 | 9.21E-05 |
| ILMN_1800787 | ENSG00000131378 | RFTN1 | 0.62 | 1.86E-07 | 3.76E-05 |
| ILMN_1782538 | ENSG00000026025 | VIM | -0.62 | 1.03E-03 | 2.85E-02 |
| ILMN_1752502 | ENSG00000156510 | HKDC1 | -0.62 | 9.78E-04 | 2.75E-02 |
| ILMN_1695763 | ENSG00000065485 | PDIA5 | 0.62 | 3.15E-05 | 2.08E-03 |
| ILMN_1710514 | ENSG00000069399 | BCL3 | 0.62 | 1.71E-03 | 3.99E-02 |
| ILMN_1681136 | ENSG00000140481 | CCDC33 | -0.62 | 1.07E-03 | 2.92E-02 |
| ILMN_1698934 | ENSG00000153551 | CMTM7 | 0.62 | 2.28E-04 | 9.61E-03 |
| ILMN_1705201 | ENSG00000176692 | FOXC2 | -0.62 | 6.24E-04 | 1.98E-02 |
| ILMN_3289508 |  | CR604709 | -0.62 | 1.35E-05 | 1.05E-03 |
| ILMN_1790771 | ENSG00000211777 | TRV3J34 | -0.62 | 5.33E-04 | 1.78E-02 |
| ILMN_1695079 | ENSG00000181896 | ZNF101 | 0.62 | 2.05E-05 | 1.48E-03 |
| ILMN_1746686 | ENSG00000171453 | POLR1C | 0.61 | 5.24E-06 | 5.26E-04 |
| ILMN_2055523 | ENSG00000147408 | ChGn | -0.61 | 3.33E-05 | 2.14E-03 |
| ILMN_1813455 | ENSG00000135899 | SP110 | 0.61 | 1.86E-05 | 1.37E-03 |
| ILMN_1671854 |  | DKFZp666C094 | -0.61 | 2.96E-04 | 1.17E-02 |
| ILMN_1664265 | ENSG00000146904 | EPHA1 | -0.61 | 5.33E-04 | 1.78E-02 |
| ILMN_1860638 | ENSG00000008513 | ST3GAL1 | -0.61 | 1.20E-03 | 3.15E-02 |
| ILMN_2183409 | ENSG00000073060 | UBC | 0.61 | 1.09E-04 | 5.37E-03 |
| ILMN_1704055 | ENSG00000048162 | NOP16 | 0.61 | 6.00E-04 | 1.94E-02 |
| ILMN_1805766 | ENSG00000184271 | POU6F1 | -0.61 | 2.14E-03 | 4.71E-02 |
| ILMN_1748651 | ENSG00000108294 | PSMB3 | 0.61 | 2.72E-05 | 1.86E-03 |
| ILMN_2206344 | ENSG00000166507 | NDST2 | 0.61 | 1.75E-04 | 7.88E-03 |
| ILMN_1664167 | ENSG00000197498 | RPF2 | 0.61 | 4.18E-04 | 1.49E-02 |
| ILMN_2346997 | ENSG00000112210 | RAB23 | -0.61 | 9.18E-05 | 4.78E-03 |
| ILMN_1668984 | ENSG00000182557 | SPNS3 | -0.61 | 7.76E-05 | 4.17E-03 |
| ILMN_1811702 | ENSG00000030582 | GRN | -0.61 | 3.94E-04 | 1.43E-02 |
| ILMN_1737418 | ENSG00000164398 | ACSL6 | 0.61 | 5.89E-04 | 1.92E-02 |
| ILMN_1753241 | ENSG00000101400 | SNTA1 | 0.61 | 1.17E-04 | 5.63E-03 |
| ILMN_3177354 |  | LOC100128842 | 0.60 | 1.41E-03 | 3.46E-02 |
| ILMN_2054392 | ENSG00000137168 | PPIL1 | 0.60 | 2.04E-04 | 8.82E-03 |
| ILMN_2334989 | ENSG00000163468 | CCT3 | 0.60 | 1.09E-05 | 8.91E-04 |
| ILMN_1757317 | ENSG00000133706 | LARS | 0.60 | 4.80E-06 | 4.94E-04 |
| ILMN_2187487 | ENSG00000008869 | HEATR5B | -0.60 | 3.32E-05 | 2.14E-03 |
| ILMN_1702171 | ENSG00000153395 | LPCAT1 | 0.60 | 1.04E-04 | 5.22E-03 |
| ILMN_2347298 | ENSG00000184205 | TSPYL2 | -0.60 | 1.79E-03 | 4.13E-02 |
| ILMN_1664802 | ENSG00000109046 | WSB1 | -0.60 | 6.93E-05 | 3.81E-03 |
| ILMN_2375003 | ENSG00000233404 | MAP4K4 | -0.60 | 9.56E-06 | 8.14E-04 |
| ILMN_1761733 | ENSG00000242092 | HLA-DMB | -0.60 | 1.24E-03 | 3.22E-02 |
| ILMN_1805916 | ENSG00000184117 | NIPSNAP1 | 0.60 | 1.08E-04 | 5.32E-03 |
| ILMN_1693108 | ENSG00000175792 | RUVBL1 | 0.60 | 7.17E-06 | 6.68E-04 |
| ILMN_1692754 | ENSG00000062716 | VMP1 | 0.60 | 2.30E-03 | 4.93E-02 |
| ILMN_1737462 | ENSG00000164830 | OXR1 | 0.60 | 1.10E-05 | 8.95E-04 |
| ILMN_1777519 | ENSG00000139626 | ITGB7 | -0.60 | 3.40E-05 | 2.18E-03 |
| ILMN_1712400 | ENSG00000124570 | SERPINB6 | 0.60 | 3.74E-04 | 1.37E-02 |
| ILMN_1745806 | ENSG00000133027 | PEMT | 0.60 | 4.85E-05 | 2.85E-03 |
| ILMN_1751656 | ENSG00000172059 | KLF11 | 0.60 | 6.00E-04 | 1.94E-02 |
| ILMN_1761010 | ENSG00000114054 | PCCB | 0.60 | 1.12E-05 | 9.06E-04 |
| ILMN_1798256 | ENSG00000183696 | UPP1 | 0.59 | 3.43E-04 | 1.28E-02 |
| ILMN_1673991 | ENSG00000138363 | ATIC | 0.59 | 5.27E-07 | 8.17E-05 |
| ILMN_1651254 | ENSG00000145012 | LPP | -0.59 | 1.25E-05 | 9.83E-04 |
| ILMN_1783806 | ENSG00000047579 | DTNBP1 | 0.59 | 9.13E-04 | 2.61E-02 |
| ILMN_2405628 | ENSG00000184428 | TOP1MT | 0.59 | 4.90E-05 | 2.88E-03 |
| ILMN_1731742 | ENSG00000159958 | TNFRSF13C | 0.59 | 4.49E-04 | 1.57E-02 |
| ILMN_2336781 | ENSG00000112096 | SOD2 | 0.59 | 9.52E-05 | 4.90E-03 |
| ILMN_1791211 | ENSG00000147443 | DOK2 | -0.59 | 5.12E-05 | 2.97E-03 |
| ILMN_1728724 | ENSG00000177663 | IL17RA | -0.59 | 1.82E-05 | 1.36E-03 |
| ILMN_1760062 | ENSG00000137965 | IFI44 | -0.59 | 1.28E-03 | 3.27E-02 |
| ILMN_1737580 | ENSG00000100418 | PPPDE2 | 0.59 | 3.65E-05 | 2.29E-03 |
| ILMN_1676091 | ENSG00000215492 | HNRNPA1 | 0.59 | 2.36E-05 | 1.64E-03 |
| ILMN_1664859 | ENSG00000111728 | ST8SIA1 | 0.59 | 1.35E-05 | 1.05E-03 |
| ILMN_1728298 | ENSG00000188322 | SBK1 | -0.59 | 9.84E-04 | 2.76E-02 |
| ILMN_1778575 | ENSG00000154764 | WNT7A | -0.59 | 9.41E-04 | 2.68E-02 |
| ILMN_2344373 | ENSG00000013364 | MVP | 0.59 | 1.69E-04 | 7.67E-03 |
| ILMN_2075189 | ENSG00000110660 | SLC35F2 | 0.59 | 2.28E-03 | 4.89E-02 |
| ILMN_1772359 | ENSG00000162511 | LAPTM5 | -0.59 | 1.05E-05 | 8.69E-04 |
| ILMN_2130180 | ENSG00000240370 | RPL13P5 | 0.59 | 1.54E-05 | 1.17E-03 |
| ILMN_1710434 | ENSG00000175463 | TBC1D10C | -0.59 | 4.43E-04 | 1.55E-02 |
| ILMN_1675541 | ENSG00000232472 | EEF1B2 | 0.59 | 2.09E-03 | 4.64E-02 |
| ILMN_1726410 | ENSG00000198931 | APRT | 0.59 | 8.54E-07 | 1.24E-04 |

***Supplementary Table 4*.** *List of genes differentially expressed in memory CD4+ T-cells 72 hours following exposure to 0.5ng/ml IL-6 and equimolar sIL-6R compared to 72 hours untreated memory CD4+ T-cells. Differentially expressed genes were identified post multiple test correction by cross-sectional analysis of untreated and IL-6 exposed memory CD4+ T-cells following 72 hours exposure to 0.5ng/ml IL-6 using moderated paired t-test with fold change over 1.5 and corrected p value cut off of <0.05.* *N=3.*

| Illumina ID | Ensembl ID | Gene | logFC | P.Value | adj.P.Value |
| --- | --- | --- | --- | --- | --- |
| ILMN_1737314 | ENSG00000113916 | BCL6 | 1.84 | 1.37E-11 | 4.70E-08 |
| ILMN_1780349 | ENSG00000116690 | PRG4 | 1.65 | 3.99E-14 | 4.11E-10 |
| ILMN_1724533 | ENSG00000154589 | LY96 | 1.56 | 1.19E-13 | 8.18E-10 |
| ILMN_1710124 | ENSG00000170293 | CMTM8 | 1.50 | 6.18E-13 | 3.18E-09 |
| ILMN_1757604 | ENSG00000198467 | TPM2 | -1.45 | 1.01E-09 | 2.08E-06 |
| ILMN_1704537 | ENSG00000092621 | PHGDH | 1.40 | 1.15E-08 | 1.58E-05 |
| ILMN_1801246 | ENSG00000185885 | IFITM1 | 1.38 | 1.68E-16 | 3.45E-12 |
| ILMN_1808811 | ENSG00000064932 | SBNO2 | 1.36 | 4.09E-10 | 1.05E-06 |
| ILMN_1720373 | ENSG00000103257 | SLC7A5 | 1.36 | 3.81E-07 | 2.18E-04 |
| ILMN_1756992 | ENSG00000185499 | MUC1 | 1.35 | 1.32E-11 | 4.70E-08 |
| ILMN_1789196 | ENSG00000198467 | TPM2 | -1.31 | 2.43E-08 | 2.31E-05 |
| ILMN_1692938 | ENSG00000135069 | PSAT1 | 1.30 | 3.92E-07 | 2.18E-04 |
| ILMN_2405521 | ENSG00000065911 | MTHFD2 | 1.27 | 3.06E-08 | 2.74E-05 |
| ILMN_1772521 | ENSG00000120254 | MTHFD1L | 1.26 | 3.24E-11 | 9.53E-08 |
| ILMN_1731224 | ENSG00000138496 | PARP9 | 1.25 | 4.62E-07 | 2.44E-04 |
| ILMN_1748283 | ENSG00000102096 | PIM2 | 1.22 | 2.46E-08 | 2.31E-05 |
| ILMN_2086095 | ENSG00000115738 | ID2 | 1.21 | 1.44E-06 | 6.32E-04 |
| ILMN_1680618 | ENSG00000136997 | MYC | 1.17 | 2.54E-07 | 1.58E-04 |
| ILMN_1793990 | ENSG00000115738 | ID2 | 1.17 | 8.60E-06 | 2.43E-03 |
| ILMN_2098446 | ENSG00000141682 | PMAIP1 | 1.16 | 1.96E-07 | 1.32E-04 |
| ILMN_2110908 | ENSG00000136997 | MYC | 1.14 | 1.41E-06 | 6.32E-04 |
| ILMN_1708004 |  | AY262164 | -1.13 | 3.80E-08 | 3.13E-05 |
| ILMN_1677765 | ENSG00000157193 | LRP8 | 1.12 | 9.38E-10 | 2.08E-06 |
| ILMN_2078592 | ENSG00000111863 | C6orf105 | 1.11 | 1.14E-05 | 3.17E-03 |
| ILMN_1674706 | ENSG00000065911 | MTHFD2 | 1.11 | 4.17E-07 | 2.26E-04 |
| ILMN_1781001 | ENSG00000184557 | SOCS3 | 1.10 | 5.37E-04 | 4.87E-02 |
| ILMN_1741133 | ENSG00000239672 | NME1 | 1.09 | 3.82E-06 | 1.38E-03 |
| ILMN_1689329 | ENSG00000099194 | SCD | 1.08 | 1.41E-05 | 3.76E-03 |
| ILMN_2302757 | ENSG00000090920 | FCGBP | -1.04 | 1.17E-04 | 1.84E-02 |
| ILMN_2285817 | ENSG00000182118 | FAM89A | 1.04 | 1.31E-08 | 1.65E-05 |
| ILMN_1864900 |  | MIAT | 1.02 | 1.10E-04 | 1.76E-02 |
| ILMN_3236694 | ENSG00000182021 | LOC100133920 | 1.00 | 1.52E-08 | 1.65E-05 |
| ILMN_1718984 | ENSG00000090920 | FCGBP | -1.00 | 1.39E-05 | 3.76E-03 |
| ILMN_1676515 | ENSG00000106348 | IMPDH1 | 0.98 | 8.93E-07 | 4.49E-04 |
| ILMN_1736311 | ENSG00000028277 | POU2F2 | -0.98 | 1.49E-08 | 1.65E-05 |
| ILMN_2053527 | ENSG00000138496 | PARP9 | 0.97 | 7.77E-06 | 2.43E-03 |
| ILMN_1692754 | ENSG00000062716 | VMP1 | 0.96 | 6.83E-06 | 2.20E-03 |
| ILMN_1710075 | ENSG00000182118 | FAM89A | 0.96 | 3.20E-08 | 2.75E-05 |
| ILMN_1772951 | ENSG00000070526 | ST6GALNAC1 | 0.95 | 1.82E-06 | 7.66E-04 |
| ILMN_3249748 | ENSG00000134333 | LDHA | 0.93 | 9.43E-09 | 1.39E-05 |
| ILMN_2144573 | ENSG00000117151 | CTBS | 0.92 | 1.16E-04 | 1.84E-02 |
| ILMN_1664859 | ENSG00000111728 | ST8SIA1 | 0.92 | 3.36E-09 | 6.29E-06 |
| ILMN_1721626 | ENSG00000150347 | ARID5B | 0.91 | 5.27E-05 | 1.04E-02 |
| ILMN_1730999 | ENSG00000047410 | TPR | 0.91 | 1.70E-06 | 7.30E-04 |
| ILMN_1664265 | ENSG00000146904 | EPHA1 | -0.90 | 2.72E-06 | 1.07E-03 |
| ILMN_2392546 | ENSG00000128050 | PAICS | 0.90 | 1.27E-07 | 9.37E-05 |
| ILMN_1662905 | ENSG00000243678 | NME1 | 0.89 | 1.50E-08 | 1.65E-05 |
| ILMN_1783149 | ENSG00000107736 | CDH23 | -0.89 | 1.10E-04 | 1.76E-02 |
| ILMN_1765326 | ENSG00000077044 | DGKD | -0.87 | 1.30E-06 | 6.32E-04 |
| ILMN_1679158 | ENSG00000165591 | FAAH2 | 0.87 | 4.03E-05 | 8.73E-03 |
| ILMN_1796013 | ENSG00000183010 | PYCR1 | 0.87 | 8.54E-06 | 2.43E-03 |
| ILMN_1752478 | ENSG00000162496 | DHRS3 | -0.86 | 2.33E-05 | 5.65E-03 |
| ILMN_1653856 | ENSG00000154127 | UBASH3B | 0.85 | 7.57E-08 | 5.77E-05 |
| ILMN_1803941 | ENSG00000121749 | TBC1D15 | 0.85 | 6.28E-09 | 9.94E-06 |
| ILMN_1719695 | ENSG00000144802 | NFKBIZ | 0.84 | 3.45E-04 | 3.71E-02 |
| ILMN_1705737 | ENSG00000178035 | IMPDH2 | 0.83 | 4.80E-08 | 3.80E-05 |
| ILMN_1652677 | ENSG00000182118 | FAM89A | 0.83 | 8.24E-06 | 2.43E-03 |
| ILMN_1807106 | ENSG00000134333 | LDHA | 0.82 | 4.51E-09 | 7.75E-06 |
| ILMN_1773760 | ENSG00000128050 | PAICS | 0.82 | 2.76E-06 | 1.07E-03 |
| ILMN_1860638 | ENSG00000008513 | ST3GAL1 | -0.81 | 4.54E-05 | 9.36E-03 |
| ILMN_1683859 | ENSG00000139514 | SLC7A1 | 0.80 | 1.43E-06 | 6.32E-04 |
| ILMN_1691693 | ENSG00000160856 | FCRH3 | 0.80 | 3.17E-05 | 7.15E-03 |
| ILMN_1714445 | ENSG00000196517 | SLC6A9 | 0.80 | 1.37E-06 | 6.32E-04 |
| ILMN_1670256 | ENSG00000152256 | PDK1 | 0.79 | 5.79E-05 | 1.12E-02 |
| ILMN_2111739 | ENSG00000140400 | MAN2C1 | -0.79 | 1.39E-06 | 6.32E-04 |
| ILMN_1682792 | ENSG00000112578 | BYSL | 0.79 | 1.49E-04 | 2.21E-02 |
| ILMN_2199313 | ENSG00000107281 | NPDC1 | 0.78 | 5.30E-04 | 4.83E-02 |
| ILMN_1770641 | ENSG00000146021 | KLHL3 | -0.78 | 3.91E-07 | 2.18E-04 |
| ILMN_1712431 | ENSG00000179715 | FAM113B | 0.78 | 5.69E-05 | 1.12E-02 |
| ILMN_1695079 | ENSG00000181896 | ZNF101 | 0.78 | 3.82E-07 | 2.18E-04 |
| ILMN_1693014 | ENSG00000172216 | CEBPB | 0.78 | 6.23E-06 | 2.04E-03 |
| ILMN_1672661 | ENSG00000135899 | SP110 | 0.78 | 2.63E-06 | 1.06E-03 |
| ILMN_3229467 | ENSG00000123179 | EBPL | 0.77 | 2.03E-06 | 8.36E-04 |
| ILMN_1732296 | ENSG00000117318 | ID3 | -0.77 | 2.20E-04 | 2.84E-02 |
| ILMN_2338197 | ENSG00000140471 | LINS | 0.76 | 4.65E-06 | 1.62E-03 |
| ILMN_3244154 |  | MIR3651 | 0.76 | 2.68E-05 | 6.42E-03 |
| ILMN_3221432 | ENSG00000116690 | PRG4 | 0.76 | 3.79E-06 | 1.38E-03 |
| ILMN_2368530 | ENSG00000008517 | IL32 | 0.75 | 2.30E-04 | 2.88E-02 |
| ILMN_1758418 | ENSG00000102524 | TNFSF13B | 0.75 | 4.50E-05 | 9.36E-03 |
| ILMN_1749009 | ENSG00000076043 | REXO2 | 0.75 | 1.75E-08 | 1.80E-05 |
| ILMN_1685122 | ENSG00000049089 | COL9A2 | -0.74 | 1.48E-04 | 2.21E-02 |
| ILMN_1714433 | ENSG00000175130 | MARCKSL1 | -0.74 | 7.65E-07 | 3.94E-04 |
| ILMN_1684158 | ENSG00000166123 | GPT2 | 0.74 | 4.50E-05 | 9.36E-03 |
| ILMN_1805973 | ENSG00000183150 | GPR19 | 0.73 | 3.23E-06 | 1.21E-03 |
| ILMN_2067682 |  | LOC401431 | -0.73 | 1.85E-05 | 4.63E-03 |
| ILMN_2199389 | ENSG00000114812 | VIPR1 | -0.73 | 6.38E-05 | 1.20E-02 |
| ILMN_1673357 | ENSG00000101082 | SLA2 | -0.72 | 3.00E-05 | 6.89E-03 |
| ILMN_1771026 | ENSG00000106105 | GARS | 0.72 | 2.62E-04 | 3.08E-02 |
| ILMN_1726547 | ENSG00000197442 | MAP3K5 | 0.72 | 1.99E-04 | 2.67E-02 |
| ILMN_1663035 | ENSG00000072310 | SREBF1 | 0.72 | 2.99E-05 | 6.89E-03 |
| ILMN_1707720 | ENSG00000105281 | SLC1A5 | 0.72 | 1.70E-04 | 2.40E-02 |
| ILMN_1802646 | ENSG00000106123 | EPHB6 | -0.72 | 4.53E-04 | 4.56E-02 |
| ILMN_1803988 | ENSG00000143384 | MCL1 | 0.71 | 1.99E-07 | 1.32E-04 |
| ILMN_1694589 | ENSG00000170915 | PAQR8 | -0.71 | 4.02E-05 | 8.73E-03 |
| ILMN_2371911 | ENSG00000185499 | MUC1 | 0.70 | 4.36E-04 | 4.47E-02 |
| ILMN_1787815 | ENSG00000101255 | TRIB3 | 0.70 | 6.59E-05 | 1.22E-02 |
| ILMN_1800993 | ENSG00000103351 | CLUAP1 | 0.70 | 8.50E-05 | 1.45E-02 |
| ILMN_1652906 | ENSG00000148288 | GBGT1 | 0.70 | 4.82E-06 | 1.65E-03 |
| ILMN_2415144 | ENSG00000135899 | SP110 | 0.69 | 2.07E-07 | 1.33E-04 |
| ILMN_1800276 | ENSG00000049449 | RCN1 | 0.69 | 1.32E-05 | 3.64E-03 |
| ILMN_1775708 | ENSG00000059804 | SLC2A3 | 0.69 | 4.89E-04 | 4.63E-02 |
| ILMN_1767658 | ENSG00000147576 | RRS1 | 0.68 | 5.81E-05 | 1.12E-02 |
| ILMN_2282077 | ENSG00000197530 | MIB2 | -0.68 | 1.36E-04 | 2.09E-02 |
| ILMN_1791211 | ENSG00000147443 | DOK2 | -0.67 | 8.60E-06 | 2.43E-03 |
| ILMN_2391419 | ENSG00000054654 | SYNE2 | 0.67 | 1.75E-05 | 4.44E-03 |
| ILMN_1779558 | ENSG00000183087 | GAS6 | -0.67 | 4.39E-05 | 9.33E-03 |
| ILMN_1677997 | ENSG00000103495 | MAZ | 0.67 | 3.66E-05 | 8.10E-03 |
| ILMN_1733956 | ENSG00000196305 | IARS | 0.66 | 8.27E-06 | 2.43E-03 |
| ILMN_1764769 | ENSG00000110002 | VWA5A | 0.66 | 1.66E-05 | 4.27E-03 |
| ILMN_1805922 | ENSG00000123179 | EBPL | 0.66 | 8.50E-06 | 2.43E-03 |
| ILMN_1767556 | ENSG00000165507 | C10orf10 | 0.66 | 5.46E-04 | 4.91E-02 |
| ILMN_1694432 | ENSG00000182809 | CRIP2 | 0.66 | 4.95E-04 | 4.64E-02 |
| ILMN_1748258 |  | LINS1 | 0.65 | 1.07E-04 | 1.73E-02 |
| ILMN_1784780 | ENSG00000079432 | CIC | 0.65 | 5.02E-05 | 1.01E-02 |
| ILMN_1773567 | ENSG00000130702 | LAMA5 | -0.65 | 2.70E-04 | 3.13E-02 |
| ILMN_1789338 | ENSG00000120896 | SORBS3 | -0.64 | 4.97E-05 | 1.01E-02 |
| ILMN_1745075 | ENSG00000089157 | RPLP0 | 0.64 | 2.40E-04 | 2.96E-02 |
| ILMN_1784749 | ENSG00000183087 | GAS6 | -0.64 | 6.99E-05 | 1.26E-02 |
| ILMN_1673352 | ENSG00000185201 | IFITM2 | 0.64 | 3.24E-06 | 1.21E-03 |
| ILMN_1657554 | ENSG00000184205 | TSPYL2 | -0.64 | 2.77E-04 | 3.15E-02 |
| ILMN_2414007 | ENSG00000243678 | NME1 | 0.64 | 6.12E-06 | 2.03E-03 |
| ILMN_1655961 |  | NAG8 | 0.64 | 1.86E-04 | 2.52E-02 |
| ILMN_1736178 | ENSG00000106624 | AEBP1 | -0.64 | 2.58E-04 | 3.08E-02 |
| ILMN_2403247 | ENSG00000153551 | CMTM7 | 0.63 | 6.76E-05 | 1.23E-02 |
| ILMN_2342033 | ENSG00000158769 | F11R | -0.63 | 3.23E-04 | 3.54E-02 |
| ILMN_1657810 | ENSG00000164088 | PPM1M | -0.63 | 3.01E-05 | 6.89E-03 |
| ILMN_1711608 | ENSG00000145687 | SSBP2 | -0.63 | 8.29E-05 | 1.44E-02 |
| ILMN_2195482 | ENSG00000167535 | CACNB3 | -0.63 | 7.42E-05 | 1.33E-02 |
| ILMN_1664167 | ENSG00000197498 | RPF2 | 0.63 | 2.77E-04 | 3.15E-02 |
| ILMN_1695962 | ENSG00000146828 | SLC12A9 | 0.63 | 6.69E-05 | 1.23E-02 |
| ILMN_3249435 | ENSG00000154127 | UBASH3B | 0.63 | 1.61E-04 | 2.34E-02 |
| ILMN_1784333 | ENSG00000138593 | SECISBP2L | -0.63 | 7.25E-06 | 2.30E-03 |
| ILMN_1698934 | ENSG00000153551 | CMTM7 | 0.63 | 2.05E-04 | 2.71E-02 |
| ILMN_2226271 | ENSG00000248099 | INSL3 | 0.63 | 3.14E-04 | 3.46E-02 |
| ILMN_2234873 | ENSG00000011052 | NME1 | 0.63 | 3.07E-04 | 3.40E-02 |
| ILMN_1783798 | ENSG00000141013 | GAS8 | 0.62 | 1.99E-04 | 2.67E-02 |
| ILMN_1661359 | ENSG00000204165 | CXorf65 | -0.62 | 1.71E-04 | 2.40E-02 |
| ILMN_1671791 | ENSG00000100889 | PCK2 | 0.62 | 8.33E-05 | 1.44E-02 |
| ILMN_1731418 | ENSG00000135899 | SP110 | 0.61 | 1.45E-07 | 1.03E-04 |
| ILMN_1707748 | ENSG00000198355 | PIM3 | 0.61 | 1.75E-04 | 2.42E-02 |
| ILMN_1798557 | ENSG00000182230 | FAM153A | -0.61 | 2.67E-04 | 3.10E-02 |
| ILMN_1655611 | ENSG00000182463 | TSHZ2 | 0.61 | 5.30E-04 | 4.83E-02 |
| ILMN_1757317 | ENSG00000133706 | LARS | 0.61 | 4.11E-06 | 1.46E-03 |
| ILMN_1807972 | ENSG00000135596 | MICAL1 | -0.61 | 2.36E-04 | 2.92E-02 |
| ILMN_2352009 | ENSG00000072778 | ACADVL | -0.61 | 1.85E-04 | 2.52E-02 |
| ILMN_2367469 | ENSG00000110619 | CARS | 0.60 | 1.52E-04 | 2.22E-02 |
| ILMN_1792538 | ENSG00000173762 | CD7 | 0.60 | 1.46E-04 | 2.21E-02 |
| ILMN_2227195 | ENSG00000139537 | CCDC65 | -0.60 | 2.65E-04 | 3.10E-02 |
| ILMN_1815023 | ENSG00000137193 | PIM1 | 0.60 | 4.69E-04 | 4.61E-02 |
| ILMN_1754538 | ENSG00000122378 | C10orf58 | 0.60 | 6.22E-05 | 1.19E-02 |
| ILMN_1730054 | ENSG00000184674 | GSTT1 | -0.59 | 2.89E-04 | 3.25E-02 |
| ILMN_2388975 | ENSG00000100422 | CERK | -0.59 | 3.19E-05 | 7.15E-03 |
| ILMN_1739241 | ENSG00000128965 | CHAC1 | 0.59 | 8.80E-05 | 1.49E-02 |

***Supplementary Table 5*.** *List of genes differentially expressed in naïve CD4+ T-cells following 72 hours exposure to 0.5ng/ml IL-6 and equimolar sIL-6R and subsequent 4 hours TCR-stimulation compared to untreated naïve CD4+ T-cells following 4 hours TCR stimulation. Differentially expressed genes were identified post multiple test correction by cross-sectional analysis of untreated and IL-6 exposed naïve CD4+ T-cells following 72 hours exposure to 0.5ng/ml IL-6 and 4 hours TCR stimulation using moderated paired t-test with fold change over 1.5 and corrected p value cut off of <0.05.* *N=3.*

| Illumina ID | Ensembl ID | Gene | logFC | P.Value | adj.P.Value |
| --- | --- | --- | --- | --- | --- |
| ILMN_2393765 | ENSG00000128322 | IGLL1 | 3.84 | 2.63E-07 | 1.55E-04 |
| ILMN_1652199 | ENSG00000211632 | abParts | 3.12 | 9.54E-08 | 7.86E-05 |
| ILMN_1739508 | ENSG00000241755 | abParts | 2.42 | 2.26E-06 | 6.47E-04 |
| ILMN_2105441 | ENSG00000132465 | IGJ | 2.24 | 8.36E-06 | 1.81E-03 |
| ILMN_3240375 | ENSG00000242766 | abParts | 1.90 | 1.30E-05 | 2.45E-03 |
| ILMN_1679826 | ENSG00000077984 | CST7 | 1.84 | 5.02E-09 | 8.45E-06 |
| ILMN_1680274 | ENSG00000239975 | abParts | 1.81 | 7.18E-05 | 7.59E-03 |
| ILMN_1804601 | ENSG00000211893 | DKFZp686O16217 | 1.78 | 6.32E-06 | 1.51E-03 |
| ILMN_1864900 |  | MIAT | 1.70 | 1.66E-08 | 2.14E-05 |
| ILMN_1789196 | ENSG00000198467 | TPM2 | -1.68 | 8.04E-11 | 4.14E-07 |
| ILMN_1757604 | ENSG00000198467 | TPM2 | -1.64 | 5.76E-11 | 3.96E-07 |
| ILMN_1684158 | ENSG00000166123 | GPT2 | 1.43 | 1.37E-10 | 4.39E-07 |
| ILMN_2342579 | ENSG00000168685 | IL7R | -1.36 | 5.32E-04 | 2.56E-02 |
| ILMN_2186137 | ENSG00000166592 | RRAD | 1.32 | 1.49E-10 | 4.39E-07 |
| ILMN_1787815 | ENSG00000101255 | TRIB3 | 1.27 | 1.23E-09 | 2.81E-06 |
| ILMN_2319000 | ENSG00000007264 | MATK | 1.16 | 1.96E-06 | 5.86E-04 |
| ILMN_1659888 | ENSG00000173457 | PPP1R14B | 1.10 | 1.27E-08 | 1.74E-05 |
| ILMN_1662905 | ENSG00000243678 | NME1 | 1.08 | 2.21E-10 | 5.69E-07 |
| ILMN_1788108 | ENSG00000259040 | TXNDC5 | 1.07 | 1.17E-06 | 4.29E-04 |
| ILMN_1711120 | ENSG00000198576 | ARC | 1.05 | 1.06E-07 | 8.39E-05 |
| ILMN_2394250 | ENSG00000107679 | PLEKHA1 | -1.05 | 6.24E-04 | 2.79E-02 |
| ILMN_3305273 | ENSG00000230787 | PSAT1 | 1.03 | 1.03E-04 | 9.82E-03 |
| ILMN_1695034 | ENSG00000218574 | HNRNPA1L2 | 1.03 | 1.72E-04 | 1.33E-02 |
| ILMN_1686811 | ENSG00000174495 | PPIA | 1.03 | 8.73E-07 | 3.59E-04 |
| ILMN_2227573 | ENSG00000148834 | GSTO1 | -1.02 | 1.34E-12 | 2.75E-08 |
| ILMN_1704537 | ENSG00000092621 | PHGDH | 1.00 | 6.80E-06 | 1.57E-03 |
| ILMN_3292224 | ENSG00000237285 | HNRNPA1 | 0.99 | 7.83E-04 | 3.27E-02 |
| ILMN_1663068 | ENSG00000248321 | C1orf228 | -0.98 | 1.07E-10 | 4.39E-07 |
| ILMN_2374352 | ENSG00000003249 | DBNDD1 | 0.98 | 1.31E-04 | 1.15E-02 |
| ILMN_1772869 | ENSG00000101230 | ISM1 | -0.98 | 5.13E-08 | 4.80E-05 |
| ILMN_2111187 | ENSG00000170522 | ELOVL6 | 0.97 | 3.40E-07 | 1.86E-04 |
| ILMN_1753111 | ENSG00000105835 | NAMPT | 0.97 | 4.46E-05 | 5.50E-03 |
| ILMN_3243291 | ENSG00000237285 | HNRNPA1 | 0.97 | 1.17E-03 | 4.28E-02 |
| ILMN_1798557 | ENSG00000182230 | FAM153A | -0.96 | 2.42E-07 | 1.52E-04 |
| ILMN_2363591 | ENSG00000137575 | SDCBP | 0.95 | 5.27E-04 | 2.55E-02 |
| ILMN_1756992 | ENSG00000185499 | MUC1 | 0.95 | 4.94E-08 | 4.80E-05 |
| ILMN_1689004 | ENSG00000006327 | TNFRSF12A | 0.95 | 3.66E-04 | 2.08E-02 |
| ILMN_1658399 | ENSG00000139187 | KLRG1 | -0.94 | 1.66E-05 | 2.88E-03 |
| ILMN_1688780 | ENSG00000196154 | S100A4 | 0.94 | 1.90E-06 | 5.83E-04 |
| ILMN_1724533 | ENSG00000154589 | LY96 | 0.93 | 3.63E-08 | 4.16E-05 |
| ILMN_2099528 | ENSG00000186265 | BTLA | -0.93 | 9.68E-05 | 9.32E-03 |
| ILMN_1766169 | ENSG00000060982 | BCAT1 | 0.93 | 3.17E-05 | 4.38E-03 |
| ILMN_1715947 | ENSG00000220157 | HNRNPA1 | 0.92 | 1.31E-04 | 1.15E-02 |
| ILMN_1786612 | ENSG00000248988 | PSME2 | 0.92 | 5.14E-07 | 2.58E-04 |
| ILMN_1711030 | ENSG00000178814 | OPLAH | 0.92 | 4.28E-06 | 1.10E-03 |
| ILMN_1735093 | ENSG00000111602 | TIMELESS | -0.91 | 4.11E-08 | 4.46E-05 |
| ILMN_1772359 | ENSG00000162511 | LAPTM5 | -0.90 | 2.80E-09 | 5.24E-06 |
| ILMN_1737580 | ENSG00000100418 | PPPDE2 | 0.90 | 2.23E-08 | 2.70E-05 |
| ILMN_1778536 | ENSG00000186265 | BTLA | -0.90 | 2.75E-04 | 1.76E-02 |
| ILMN_1757186 | ENSG00000213203 | GIMAP5 | -0.89 | 2.00E-05 | 3.22E-03 |
| ILMN_1801246 | ENSG00000185885 | IFITM1 | 0.89 | 3.16E-11 | 3.25E-07 |
| ILMN_1686664 | ENSG00000125148 | MT2A | 0.89 | 3.42E-05 | 4.51E-03 |
| ILMN_1821270 | ENSG00000211848 | TCRA | -0.89 | 1.13E-03 | 4.18E-02 |
| ILMN_2391419 | ENSG00000054654 | SYNE2 | 0.89 | 1.21E-07 | 9.25E-05 |
| ILMN_2336609 | ENSG00000137501 | SYTL2 | -0.89 | 1.87E-04 | 1.40E-02 |
| ILMN_1741727 | ENSG00000115828 | QPCT | -0.88 | 1.74E-05 | 2.97E-03 |
| ILMN_1677765 | ENSG00000157193 | LRP8 | 0.87 | 1.94E-07 | 1.38E-04 |
| ILMN_2367883 | ENSG00000164949 | GEM | 0.87 | 5.85E-05 | 6.65E-03 |
| ILMN_1713384 | ENSG00000227164 | TSHZ2 | 0.87 | 6.65E-06 | 1.56E-03 |
| ILMN_1787750 | ENSG00000091972 | CD200 | -0.87 | 1.44E-03 | 4.85E-02 |
| ILMN_3235176 | ENSG00000186854 | C2orf89 | -0.86 | 2.09E-07 | 1.44E-04 |
| ILMN_3298716 | ENSG00000175592 | FOSL1 | 0.86 | 4.85E-05 | 5.77E-03 |
| ILMN_1742431 |  | AY358241 | -0.86 | 1.85E-04 | 1.40E-02 |
| ILMN_1691611 | ENSG00000237285 | HNRNPA1L2 | 0.86 | 1.46E-03 | 4.87E-02 |
| ILMN_1776653 | ENSG00000047634 | SCML1 | -0.86 | 3.43E-07 | 1.86E-04 |
| ILMN_1798705 | ENSG00000112237 | CCNC | 0.86 | 8.21E-04 | 3.38E-02 |
| ILMN_2168217 | ENSG00000169508 | GPR183 | -0.85 | 2.27E-07 | 1.51E-04 |
| ILMN_1819384 |  | VTRNA1-3 | 0.85 | 1.59E-05 | 2.79E-03 |
| ILMN_1724704 | ENSG00000068400 | GRIPAP1 | -0.85 | 2.06E-05 | 3.28E-03 |
| ILMN_1661366 | ENSG00000171314 | PGAM1 | 0.85 | 9.53E-04 | 3.70E-02 |
| ILMN_1677092 | ENSG00000164949 | GEM | 0.85 | 1.01E-05 | 2.06E-03 |
| ILMN_1751726 | ENSG00000213553 | RPLP0 | 0.85 | 5.33E-09 | 8.45E-06 |
| ILMN_1808196 | ENSG00000148834 | GSTO1 | -0.84 | 2.24E-09 | 4.62E-06 |
| ILMN_1687887 | ENSG00000013275 | PSMC4 | 0.83 | 4.43E-05 | 5.50E-03 |
| ILMN_1800276 | ENSG00000049449 | RCN1 | 0.83 | 6.10E-07 | 2.99E-04 |
| ILMN_2109708 | ENSG00000025708 | TYMP | 0.83 | 7.75E-07 | 3.57E-04 |
| ILMN_1693269 | ENSG00000167414 | GNG8 | -0.83 | 9.65E-04 | 3.74E-02 |
| ILMN_1784749 | ENSG00000183087 | GAS6 | -0.82 | 1.42E-06 | 4.94E-04 |
| ILMN_2337655 | ENSG00000140105 | WARS | 0.82 | 2.24E-05 | 3.45E-03 |
| ILMN_1691156 | ENSG00000205362 | MT1A | 0.82 | 1.05E-04 | 9.82E-03 |
| ILMN_1781085 | ENSG00000035720 | STAP1 | -0.81 | 1.94E-06 | 5.86E-04 |
| ILMN_1787064 | ENSG00000132466 | ANKRD17 | -0.80 | 4.14E-04 | 2.25E-02 |
| ILMN_1727271 | ENSG00000140105 | WARS | 0.80 | 7.34E-06 | 1.66E-03 |
| ILMN_1773780 | ENSG00000103254 | FAM173A | 0.80 | 9.72E-06 | 2.02E-03 |
| ILMN_1656145 | ENSG00000120053 | GOT1 | 0.80 | 9.38E-08 | 7.86E-05 |
| ILMN_1794528 | ENSG00000142609 | KIAA1751 | -0.80 | 1.43E-04 | 1.19E-02 |
| ILMN_1684306 | ENSG00000196154 | S100A4 | 0.80 | 4.82E-05 | 5.77E-03 |
| ILMN_1659158 | ENSG00000164483 | SAMD3 | -0.80 | 5.18E-06 | 1.26E-03 |
| ILMN_1780924 | ENSG00000149150 | SLC43A1 | 0.80 | 1.07E-04 | 9.95E-03 |
| ILMN_2258816 | ENSG00000164483 | SAMD3 | -0.79 | 4.15E-07 | 2.19E-04 |
| ILMN_1725312 | ENSG00000145715 | RASA1 | -0.79 | 9.58E-09 | 1.41E-05 |
| ILMN_1662618 | ENSG00000161011 | SQSTM1 | 0.79 | 2.87E-05 | 4.11E-03 |
| ILMN_1653871 | ENSG00000105835 | NAMPT | 0.79 | 2.31E-04 | 1.56E-02 |
| ILMN_1776105 | ENSG00000146733 | PSPH | 0.79 | 1.58E-05 | 2.79E-03 |
| ILMN_1772521 | ENSG00000120254 | MTHFD1L | 0.78 | 1.06E-06 | 3.97E-04 |
| ILMN_1757370 | ENSG00000166311 | SMPD1 | -0.78 | 9.18E-06 | 1.93E-03 |
| ILMN_1661599 | ENSG00000168209 | DDIT4 | 0.78 | 1.04E-03 | 3.93E-02 |
| ILMN_1655611 | ENSG00000182463 | TSHZ2 | 0.78 | 2.55E-05 | 3.76E-03 |
| ILMN_2193233 | ENSG00000170476 | MZB1 | 0.77 | 2.72E-04 | 1.75E-02 |
| ILMN_1687978 | ENSG00000139289 | PHLDA1 | 0.77 | 7.36E-05 | 7.69E-03 |
| ILMN_1720745 | ENSG00000219582 | HNRNPA1 | 0.77 | 7.82E-04 | 3.27E-02 |
| ILMN_3245559 | ENSG00000235423 | CDK2AP1 | 0.77 | 2.62E-07 | 1.55E-04 |
| ILMN_1831563 | ENSG00000225948 | CR749724 | -0.77 | 2.84E-06 | 7.90E-04 |
| ILMN_1794707 | ENSG00000142102 | ATHL1 | -0.77 | 1.29E-05 | 2.45E-03 |
| ILMN_1665601 | ENSG00000176946 | THAP4 | 0.77 | 2.84E-07 | 1.63E-04 |
| ILMN_1769810 | ENSG00000144746 | ARL6IP5 | -0.77 | 7.78E-06 | 1.72E-03 |
| ILMN_1656131 |  | FLJ22536 | 0.77 | 2.09E-05 | 3.31E-03 |
| ILMN_1739640 | ENSG00000166341 | DCHS1 | -0.77 | 1.35E-04 | 1.16E-02 |
| ILMN_1738816 | ENSG00000150907 | FOXO1 | -0.76 | 8.57E-07 | 3.59E-04 |
| ILMN_2393296 | ENSG00000178146 | GK | 0.76 | 3.52E-04 | 2.07E-02 |
| ILMN_1858272 |  | AI218425 | 0.75 | 3.37E-05 | 4.48E-03 |
| ILMN_1771841 | ENSG00000175592 | FOSL1 | 0.75 | 2.30E-04 | 1.56E-02 |
| ILMN_1745075 | ENSG00000089157 | RPLP0 | 0.75 | 2.93E-05 | 4.17E-03 |
| ILMN_3228822 | ENSG00000166881 | TMEM194A | -0.75 | 5.02E-07 | 2.58E-04 |
| ILMN_3247998 | ENSG00000035720 | STAP1 | -0.75 | 1.86E-05 | 3.06E-03 |
| ILMN_2345292 | ENSG00000221857 | FXYD1 | 0.75 | 2.15E-05 | 3.36E-03 |
| ILMN_2374865 | ENSG00000162772 | ATF3 | 0.75 | 4.89E-04 | 2.44E-02 |
| ILMN_1775111 | ENSG00000197157 | SND1 | 0.75 | 1.27E-06 | 4.52E-04 |
| ILMN_1737517 | ENSG00000162244 | RPL29 | 0.75 | 2.44E-06 | 6.88E-04 |
| ILMN_1776838 | ENSG00000255168 | NBPF1 | -0.74 | 3.06E-04 | 1.89E-02 |
| ILMN_1661264 | ENSG00000182199 | SHMT2 | 0.74 | 7.44E-08 | 6.66E-05 |
| ILMN_1732296 | ENSG00000117318 | ID3 | -0.74 | 3.62E-04 | 2.08E-02 |
| ILMN_1805652 | ENSG00000175470 | PPP2R2D | 0.74 | 2.43E-04 | 1.62E-02 |
| ILMN_1676893 | ENSG00000138031 | ADCY3 | 0.74 | 4.66E-06 | 1.18E-03 |
| ILMN_1674402 | ENSG00000165071 | TMEM71 | -0.74 | 2.43E-07 | 1.52E-04 |
| ILMN_1726913 | ENSG00000168214 | RBPJ | 0.74 | 2.54E-04 | 1.67E-02 |
| ILMN_2410038 | ENSG00000166801 | FAM111A | -0.74 | 1.78E-06 | 5.54E-04 |
| ILMN_2203896 | ENSG00000101665 | SMAD7 | -0.73 | 1.96E-04 | 1.44E-02 |
| ILMN_1798706 | ENSG00000169508 | GPR183 | -0.73 | 8.73E-07 | 3.59E-04 |
| ILMN_2347949 | ENSG00000160211 | G6PD | 0.73 | 6.32E-05 | 7.00E-03 |
| ILMN_1740319 | ENSG00000119632 | IFI27L2 | -0.73 | 1.58E-06 | 5.34E-04 |
| ILMN_1804339 | ENSG00000008118 | CAMK1G | 0.73 | 1.50E-03 | 4.95E-02 |
| ILMN_3203126 |  | LOC100131662 | -0.72 | 2.75E-04 | 1.76E-02 |
| ILMN_2403852 | ENSG00000108669 | CYTH1 | -0.72 | 7.17E-05 | 7.59E-03 |
| ILMN_2371911 | ENSG00000185499 | MUC1 | 0.72 | 3.60E-04 | 2.07E-02 |
| ILMN_2121408 | ENSG00000113070 | HBEGF | -0.72 | 6.65E-04 | 2.91E-02 |
| ILMN_1725471 | ENSG00000178146 | GK | 0.71 | 8.18E-06 | 1.79E-03 |
| ILMN_2044226 | ENSG00000138814 | PPP3CA | -0.71 | 2.29E-04 | 1.56E-02 |
| ILMN_1661717 | ENSG00000198176 | TFDP1 | 0.71 | 2.24E-05 | 3.45E-03 |
| ILMN_1730523 | ENSG00000172366 | FAM195A | 0.71 | 4.36E-05 | 5.48E-03 |
| ILMN_1812070 | ENSG00000085563 | ABCB1 | 0.71 | 2.39E-04 | 1.61E-02 |
| ILMN_3256693 |  | AK130189 | 0.70 | 4.39E-04 | 2.29E-02 |
| ILMN_1716272 | ENSG00000163376 | KBTBD8 | 0.70 | 4.95E-04 | 2.46E-02 |
| ILMN_3306440 | ENSG00000166881 | TMEM194A | -0.70 | 1.67E-06 | 5.39E-04 |
| ILMN_2109371 | ENSG00000175868 | CALCB | 0.70 | 6.52E-05 | 7.09E-03 |
| ILMN_1723962 | ENSG00000079257 | LXN | -0.70 | 7.40E-07 | 3.55E-04 |
| ILMN_1787345 | ENSG00000255863 | FKBP11 | 0.70 | 1.48E-07 | 1.09E-04 |
| ILMN_2072296 | ENSG00000123975 | CKS2 | 0.70 | 3.16E-04 | 1.94E-02 |
| ILMN_3302456 | ENSG00000243175 | RPSA | 0.70 | 1.54E-04 | 1.25E-02 |
| ILMN_1722634 | ENSG00000104805 | NUCB1 | 0.70 | 2.92E-06 | 7.97E-04 |
| ILMN_1699695 | ENSG00000146072 | TNFRSF21 | 0.69 | 6.66E-05 | 7.18E-03 |
| ILMN_1779558 | ENSG00000183087 | GAS6 | -0.69 | 2.50E-05 | 3.71E-03 |
| ILMN_1660907 | ENSG00000132334 | PTPRE | -0.69 | 4.23E-04 | 2.28E-02 |
| ILMN_2328094 | ENSG00000165617 | DACT1 | -0.69 | 1.57E-05 | 2.79E-03 |
| ILMN_3248511 | ENSG00000154319 | FAM167A | 0.69 | 3.09E-04 | 1.90E-02 |
| ILMN_1677997 | ENSG00000103495 | MAZ | 0.69 | 2.27E-05 | 3.46E-03 |
| ILMN_2331636 | ENSG00000132142 | ACACA | 0.69 | 8.74E-07 | 3.59E-04 |
| ILMN_3271412 | ENSG00000249031 | SUMO2 | 0.69 | 1.78E-04 | 1.36E-02 |
| ILMN_1776464 | ENSG00000102699 | PARP4 | -0.69 | 2.14E-06 | 6.20E-04 |
| ILMN_2405521 | ENSG00000065911 | MTHFD2 | 0.68 | 5.20E-04 | 2.54E-02 |
| ILMN_1708004 |  | AY262164 | -0.68 | 1.56E-04 | 1.25E-02 |
| ILMN_1682929 | ENSG00000137501 | SYTL2 | -0.68 | 7.92E-04 | 3.30E-02 |
| ILMN_1794165 | ENSG00000142657 | PGD | 0.68 | 6.97E-05 | 7.47E-03 |
| ILMN_2289775 | ENSG00000143575 | HAX1 | 0.68 | 3.19E-04 | 1.95E-02 |
| ILMN_1745806 | ENSG00000133027 | PEMT | 0.68 | 6.57E-06 | 1.56E-03 |
| ILMN_1751598 | ENSG00000130766 | SESN2 | 0.68 | 6.05E-04 | 2.75E-02 |
| ILMN_1707627 | ENSG00000111669 | TPI1 | 0.68 | 1.31E-05 | 2.45E-03 |
| ILMN_2111739 | ENSG00000140400 | MAN2C1 | -0.68 | 1.68E-05 | 2.88E-03 |
| ILMN_1685810 | ENSG00000072736 | NFATC3 | -0.67 | 1.19E-06 | 4.29E-04 |
| ILMN_2360028 | ENSG00000072736 | NFATC3 | -0.67 | 1.17E-05 | 2.34E-03 |
| ILMN_3287493 | ENSG00000226961 | PPIA | 0.67 | 2.94E-06 | 7.97E-04 |
| ILMN_1792660 | ENSG00000118200 | CAMSAP1L1 | -0.67 | 1.35E-05 | 2.50E-03 |
| ILMN_1723486 | ENSG00000159399 | HK2 | 0.67 | 1.90E-04 | 1.41E-02 |
| ILMN_1794594 | ENSG00000068831 | RASGRP2 | -0.67 | 1.49E-03 | 4.93E-02 |
| ILMN_1662839 | ENSG00000107679 | PLEKHA1 | -0.67 | 5.12E-06 | 1.26E-03 |
| ILMN_1811433 | ENSG00000161016 | RPL8 | 0.67 | 2.24E-04 | 1.54E-02 |
| ILMN_1690646 | ENSG00000182568 | SATB1 | -0.67 | 1.30E-03 | 4.51E-02 |
| ILMN_1674706 | ENSG00000065911 | MTHFD2 | 0.67 | 6.55E-04 | 2.87E-02 |
| ILMN_1797172 | ENSG00000012061 | ERCC1 | 0.66 | 3.43E-04 | 2.04E-02 |
| ILMN_1718766 | ENSG00000198417 | MT1F | 0.66 | 1.04E-04 | 9.82E-03 |
| ILMN_1676765 | ENSG00000213442 | RPL18A | 0.66 | 4.67E-08 | 4.80E-05 |
| ILMN_1669321 | ENSG00000007264 | MATK | 0.66 | 1.07E-03 | 4.00E-02 |
| ILMN_1721868 | ENSG00000182481 | KPNA2 | 0.66 | 1.13E-03 | 4.18E-02 |
| ILMN_1705737 | ENSG00000178035 | IMPDH2 | 0.66 | 3.53E-06 | 9.31E-04 |
| ILMN_1720501 | ENSG00000154258 | ABCA9 | 0.66 | 1.82E-05 | 3.04E-03 |
| ILMN_1709880 | ENSG00000089157 | RPLP0 | 0.66 | 7.94E-07 | 3.57E-04 |
| ILMN_1725417 | ENSG00000184613 | NELL2 | -0.66 | 1.34E-04 | 1.16E-02 |
| ILMN_3276019 | ENSG00000243422 | RPL23AP7 | 0.66 | 3.05E-06 | 8.16E-04 |
| ILMN_2335813 | ENSG00000131979 | GCH1 | 0.65 | 3.60E-04 | 2.07E-02 |
| ILMN_1776260 | ENSG00000255854 | PPIAL4A | 0.65 | 4.36E-05 | 5.48E-03 |
| ILMN_3237698 | ENSG00000214719 | DKFZp667M2411 | -0.65 | 6.07E-05 | 6.79E-03 |
| ILMN_2246956 | ENSG00000171791 | BCL2 | -0.65 | 1.76E-04 | 1.35E-02 |
| ILMN_2199439 | ENSG00000104267 | CA2 | 0.65 | 7.72E-06 | 1.72E-03 |
| ILMN_1747023 | ENSG00000160013 | PTGIR | -0.65 | 4.36E-04 | 2.29E-02 |
| ILMN_2058468 | ENSG00000112182 | BACH2 | -0.65 | 5.18E-06 | 1.26E-03 |
| ILMN_2383058 | ENSG00000089012 | SIRPG | -0.65 | 1.24E-05 | 2.39E-03 |
| ILMN_1752478 | ENSG00000162496 | DHRS3 | -0.65 | 7.76E-04 | 3.25E-02 |
| ILMN_2360054 | ENSG00000204580 | DDR1 | 0.65 | 4.81E-05 | 5.77E-03 |
| ILMN_2383611 | ENSG00000132334 | PTPRE | -0.65 | 2.49E-04 | 1.65E-02 |
| ILMN_1791248 | ENSG00000042062 | FAM65C | 0.64 | 4.06E-04 | 2.22E-02 |
| ILMN_3210578 |  |  | 0.64 | 1.12E-04 | 1.03E-02 |
| ILMN_2360415 | ENSG00000171867 | PRNP | 0.64 | 1.24E-03 | 4.42E-02 |
| ILMN_1661500 | ENSG00000121578 | B4GALT4 | -0.64 | 8.86E-06 | 1.90E-03 |
| ILMN_1812926 | ENSG00000163297 | ANTXR2 | -0.64 | 6.54E-05 | 7.09E-03 |
| ILMN_1824898 |  | MECP2 | -0.64 | 1.38E-03 | 4.73E-02 |
| ILMN_1659936 | ENSG00000087074 | PPP1R15A | 0.64 | 1.93E-04 | 1.43E-02 |
| ILMN_1723978 | ENSG00000100097 | LGALS1 | -0.64 | 5.27E-04 | 2.55E-02 |
| ILMN_1756326 | ENSG00000123975 | CKS2 | 0.64 | 4.09E-04 | 2.24E-02 |
| ILMN_3202315 | ENSG00000232883 | RPLP0 | 0.63 | 1.66E-06 | 5.39E-04 |
| ILMN_2153787 | ENSG00000205784 | ARRDC5 | -0.63 | 2.75E-05 | 3.97E-03 |
| ILMN_1682428 | ENSG00000162639 | HENMT1 | 0.63 | 7.98E-07 | 3.57E-04 |
| ILMN_2093427 | ENSG00000138606 | SHF | -0.63 | 8.37E-04 | 3.43E-02 |
| ILMN_1714108 | ENSG00000164938 | TP53INP1 | 0.63 | 5.01E-04 | 2.48E-02 |
| ILMN_1813338 | ENSG00000089692 | LAG3 | 0.63 | 7.18E-04 | 3.07E-02 |
| ILMN_1703244 | ENSG00000140941 | MAP1LC3B | 0.63 | 3.66E-06 | 9.55E-04 |
| ILMN_2364022 | ENSG00000141526 | SLC16A3 | 0.63 | 8.41E-04 | 3.44E-02 |
| ILMN_1675168 | ENSG00000205318 |  | 0.63 | 1.26E-03 | 4.44E-02 |
| ILMN_1771051 | ENSG00000162244 | RPL29 | 0.63 | 2.86E-04 | 1.81E-02 |
| ILMN_3284584 | ENSG00000230734 | RPL10 | 0.63 | 9.69E-07 | 3.70E-04 |
| ILMN_1808041 | ENSG00000198755 | RPL10A | 0.63 | 1.39E-04 | 1.18E-02 |
| ILMN_1788778 | ENSG00000138758 | SEPT11 | -0.63 | 5.85E-05 | 6.65E-03 |
| ILMN_1653856 | ENSG00000154127 | UBASH3B | -0.62 | 1.83E-05 | 3.04E-03 |
| ILMN_1705213 | ENSG00000135926 | TMBIM1 | -0.62 | 2.01E-04 | 1.46E-02 |
| ILMN_1773991 |  |  | 0.62 | 4.64E-04 | 2.36E-02 |
| ILMN_2340908 | ENSG00000047634 | SCML1 | -0.62 | 2.41E-04 | 1.61E-02 |
| ILMN_1767006 | ENSG00000230669 | PSMB8 | 0.62 | 2.10E-04 | 1.49E-02 |
| ILMN_1691501 |  | C15orf5 | -0.62 | 7.35E-05 | 7.69E-03 |
| ILMN_1656232 | ENSG00000204677 | FAM153A | -0.62 | 6.26E-04 | 2.79E-02 |
| ILMN_3229467 | ENSG00000123179 | EBPL | 0.62 | 6.15E-05 | 6.84E-03 |
| ILMN_1709237 | ENSG00000120915 | EPHX2 | -0.62 | 2.72E-04 | 1.75E-02 |
| ILMN_1735495 | ENSG00000204634 | TBC1D8 | -0.62 | 9.09E-04 | 3.57E-02 |
| ILMN_1772123 | ENSG00000132142 | ACACA | 0.62 | 8.44E-05 | 8.40E-03 |
| ILMN_1769926 | ENSG00000113758 | DBN1 | 0.62 | 1.24E-04 | 1.12E-02 |
| ILMN_1811470 | ENSG00000100558 | PLEK2 | -0.62 | 5.48E-04 | 2.59E-02 |
| ILMN_1745112 | ENSG00000167106 | FAM102A | -0.62 | 6.46E-05 | 7.09E-03 |
| ILMN_1750722 | ENSG00000171863 | RPS7 | 0.61 | 4.46E-04 | 2.31E-02 |
| ILMN_1691341 | ENSG00000168685 | IL7R | -0.61 | 5.18E-05 | 6.06E-03 |
| ILMN_1757497 | ENSG00000128564 | VGF | 0.61 | 5.78E-04 | 2.68E-02 |
| ILMN_1676515 | ENSG00000106348 | IMPDH1 | 0.61 | 6.75E-04 | 2.94E-02 |
| ILMN_1750792 | ENSG00000103043 | VAC14 | -0.61 | 1.27E-03 | 4.45E-02 |
| ILMN_1800976 | ENSG00000072736 | NFATC3 | -0.61 | 3.28E-05 | 4.47E-03 |
| ILMN_2184184 | ENSG00000135046 | ANXA1 | -0.61 | 2.00E-04 | 1.46E-02 |
| ILMN_1719756 | ENSG00000115085 | ZAP70 | -0.61 | 1.27E-04 | 1.13E-02 |
| ILMN_2382829 | ENSG00000167815 | PRDX2 | 0.61 | 1.61E-04 | 1.28E-02 |
| ILMN_1666706 | ENSG00000235919 | LOC645676 | 0.61 | 4.24E-04 | 2.28E-02 |
| ILMN_1810107 | ENSG00000184560 | C17orf74 | 0.60 | 6.70E-04 | 2.92E-02 |
| ILMN_3282768 | ENSG00000173457 | PPP1R14B | 0.60 | 7.12E-04 | 3.05E-02 |
| ILMN_2414007 | ENSG00000243678 | NME1 | 0.60 | 1.54E-05 | 2.79E-03 |
| ILMN_2054019 | ENSG00000187608 | ISG15 | 0.60 | 3.08E-05 | 4.34E-03 |
| ILMN_1658143 | ENSG00000133119 | RFC3 | 0.60 | 1.34E-04 | 1.16E-02 |
| ILMN_2093775 | ENSG00000124203 | ZNF831 | -0.60 | 2.15E-04 | 1.51E-02 |
| ILMN_1710756 | ENSG00000074800 | ENO1 | 0.60 | 3.10E-05 | 4.35E-03 |
| ILMN_1671791 | ENSG00000100889 | PCK2 | 0.59 | 1.39E-04 | 1.18E-02 |
| ILMN_3301824 | ENSG00000198860 | TSEN15 | 0.59 | 3.44E-05 | 4.51E-03 |
| ILMN_1772466 | ENSG00000125731 | SH2D3A | -0.59 | 5.38E-04 | 2.56E-02 |
| ILMN_1756784 | ENSG00000107130 | NCS1 | 0.59 | 3.06E-04 | 1.89E-02 |
| ILMN_1692177 | ENSG00000102804 | TSC22D1 | 0.59 | 6.08E-04 | 2.76E-02 |
| ILMN_1800993 | ENSG00000103351 | CLUAP1 | 0.59 | 6.51E-04 | 2.86E-02 |
| ILMN_3307921 | ENSG00000161031 | PGLYRP2 | -0.59 | 5.64E-04 | 2.62E-02 |
| ILMN_2181748 | ENSG00000230457 | PA2G4P4 | -0.59 | 8.59E-04 | 3.48E-02 |
| ILMN_1701930 | ENSG00000114942 | EEF1B2 | 0.59 | 2.74E-05 | 3.97E-03 |
| ILMN_3236259 | ENSG00000255854 | PPIAL4A | 0.59 | 3.32E-05 | 4.48E-03 |
| ILMN_1753468 | ENSG00000135404 | CD63 | 0.59 | 8.53E-04 | 3.47E-02 |
| ILMN_1676091 | ENSG00000215492 | HNRNPA1 | 0.59 | 2.66E-05 | 3.89E-03 |
| ILMN_1653278 | ENSG00000176945 | MUC20 | -0.59 | 1.15E-03 | 4.23E-02 |
| ILMN_1774823 | ENSG00000109475 | RPL34 | 0.59 | 2.89E-04 | 1.81E-02 |
| ILMN_1812191 | ENSG00000111678 | C12orf57 | -0.59 | 9.55E-07 | 3.70E-04 |

***Supplementary Table 6*.** *List of genes differentially expressed in memory CD4+ T-cells following 72 hours exposure to 0.5ng/ml IL-6 and equimolar sIL-6R and subsequent 4 hours TCR-stimulation compared to untreated memory CD4+ T-cells following 4 hours TCR stimulation. Differentially expressed genes were identified post multiple test correction by cross-sectional analysis of untreated and IL-6 exposed memory CD4+ T-cells following 6 hours exposure to 0.5ng/ml IL-6 and 4 hours TCR stimulation using moderated paired t-test with fold change over 1.5 and corrected p value cut off of <0.05.* *N=3.*

| Illumina ID | Ensembl ID | Gene | logFC | P.Value | adj.P.Value |
| --- | --- | --- | --- | --- | --- |
| ILMN_1757604 | ENSG00000198467 | TPM2 | -1.43 | 1.43E-09 | 1.47E-05 |
| ILMN_1704537 | ENSG00000092621 | PHGDH | 1.19 | 2.98E-07 | 6.48E-04 |
| ILMN_1789196 | ENSG00000198467 | TPM2 | -1.18 | 1.91E-07 | 4.92E-04 |
| ILMN_1724533 | ENSG00000154589 | LY96 | 1.06 | 2.47E-09 | 1.70E-05 |
| ILMN_1787815 | ENSG00000101255 | TRIB3 | 1.04 | 9.64E-08 | 3.31E-04 |
| ILMN_1684158 | ENSG00000166123 | GPT2 | 0.97 | 6.25E-07 | 1.17E-03 |
| ILMN_1772521 | ENSG00000120254 | MTHFD1L | 0.96 | 1.99E-08 | 1.02E-04 |
| ILMN_1708004 |  | AY262164 | -0.86 | 5.97E-06 | 6.83E-03 |
| ILMN_1662905 | ENSG00000243678 | NME1 | 0.82 | 9.10E-08 | 3.31E-04 |
| ILMN_1756992 | ENSG00000185499 | MUC1 | 0.81 | 1.06E-06 | 1.83E-03 |
| ILMN_1705737 | ENSG00000178035 | IMPDH2 | 0.79 | 1.31E-07 | 3.87E-04 |
| ILMN_1677765 | ENSG00000157193 | LRP8 | 0.78 | 1.54E-06 | 2.43E-03 |
| ILMN_1725417 | ENSG00000184613 | NELL2 | -0.77 | 1.48E-05 | 1.27E-02 |
| ILMN_1801246 | ENSG00000185885 | IFITM1 | 0.77 | 1.33E-09 | 1.47E-05 |
| ILMN_2390609 | ENSG00000151150 | ANK3 | 0.76 | 1.84E-05 | 1.40E-02 |
| ILMN_1798557 | ENSG00000182230 | FAM153A | -0.75 | 1.61E-05 | 1.28E-02 |
| ILMN_2331636 | ENSG00000132142 | ACACA | 0.73 | 3.15E-07 | 6.48E-04 |
| ILMN_1778575 | ENSG00000154764 | WNT7A | -0.70 | 1.26E-04 | 4.89E-02 |
| ILMN_3236694 | ENSG00000182021 | LOC100133920 | 0.70 | 1.13E-05 | 1.06E-02 |
| ILMN_1655611 | ENSG00000182463 | TSHZ2 | 0.69 | 1.18E-04 | 4.85E-02 |
| ILMN_1701930 | ENSG00000114942 | EEF1B2 | 0.67 | 3.92E-06 | 5.77E-03 |
| ILMN_1727855 | ENSG00000133027 | PEMT | 0.66 | 7.78E-05 | 4.01E-02 |
| ILMN_1710216 | ENSG00000169857 | AVEN | 0.66 | 1.11E-05 | 1.06E-02 |
| ILMN_2112811 | ENSG00000241343 | RPL36A | 0.65 | 2.09E-05 | 1.54E-02 |
| ILMN_1793410 | ENSG00000172164 | SNTB1 | 0.65 | 5.37E-06 | 6.83E-03 |
| ILMN_1737580 | ENSG00000100418 | PPPDE2 | 0.65 | 1.04E-05 | 1.06E-02 |
| ILMN_1659888 | ENSG00000173457 | PPP1R14B | 0.64 | 1.27E-04 | 4.89E-02 |
| ILMN_1720266 | ENSG00000138658 | C4orf21 | -0.64 | 2.18E-05 | 1.55E-02 |
| ILMN_3235176 | ENSG00000186854 | C2orf89 | -0.64 | 3.52E-05 | 2.41E-02 |
| ILMN_1671791 | ENSG00000100889 | PCK2 | 0.61 | 1.00E-04 | 4.70E-02 |
| ILMN_1714445 | ENSG00000196517 | SLC6A9 | 0.60 | 1.14E-04 | 4.85E-02 |
| ILMN_3202315 | ENSG00000232883 | RPLP0 | 0.60 | 4.48E-06 | 6.15E-03 |
| ILMN_1770641 | ENSG00000146021 | KLHL3 | -0.59 | 3.64E-05 | 2.42E-02 |
| ILMN_1689725 | ENSG00000137818 | RPLP1 | 0.59 | 8.13E-05 | 4.01E-02 |
